# Tubular tRF‑1:30‑Gln‑CTG‑4 attenuates tubulointerstitial fibrosis by mitigating FBXO7-mediated mitophagy defect in diabetic kidney disease

**DOI:** 10.1038/s41419-026-09112-2

**Published:** 2026-07-18

**Authors:** Jia-ling Ji, Yun-yang Qiao, Jia-yue Sun, Ying Huo, Hui-min Shi, Gao-ting Qu, Shan-wen Li, Chan Huang, Yu Feng, Pei Zhang, Ling Ding, Zuo-lin Li, Ai-qing Zhang

**Affiliations:** 1 Department of Pediatrics, The Fourth Affiliated Hospital of Nanjing Medical University, Nanjing, Jiangsu China; 2 Department of Pediatric Nephrology, The Second Affiliated Hospital of Nanjing Medical University, Nanjing, Jiangsu China; 3 Department of Nephrology and Immunology, Children’s Hospital of Soochow University, Suzhou, Jiangsu China; 4 Department of Rheumatology and Immunology, The Fourth Affiliated Hospital of Soochow University (Suzhou Dushu Lake Hospital), Suzhou, Jiangsu China; 5 Institute of Nephrology, Zhong Da Hospital, Southeast University School of Medicine, Nanjing, Jiangsu China

**Keywords:** End-stage renal disease, Mitophagy

## Abstract

Tubulointerstitial fibrosis (TIF) is a crucial pathological feature and inevitable consequence of progressive diabetic kidney disease (DKD). Transfer RNA-derived fragments (tRFs), a novel class of small non-coding RNA by cleavage of tRNAs, have recently attracted attention due to their functional significance in diverse biological processes. However, the role of tRFs in TIF is largely elusive. In the present study, we found that a tRF, tRF‑1:30‑Gln‑CTG‑4, was markedly decreased in proximal renal tubules and highly correlated with TIF in DKD mice. The overexpression of tRF‑1:30‑Gln‑CTG‑4 revealed a therapeutic effect against TIF. Further, RNA pull-down and RNA-immunoprecipitation assay were performed to identify that FBXO7, an adaptor protein in the SCF E3 ligase complex, was the targeted protein of tRF‑1:30‑Gln‑CTG‑4. Mass spectrometry and co-immunoprecipitation experiments revealed that PINK1, a master mitophagy-regulating protein, acted as the substrate of FBXO7 in tubular epithelial cells. Mechanistically, elevated FBXO7 promoted mitophagy defect through mediating PINK1 ubiquitylation and proteasomal degradation. The overexpression of tRF‑1:30‑Gln‑CTG‑4 alleviated defective mitophagy. Taken together, this study not only represents a novel insight into the pathogenesis of TIF but also provides a promising therapeutic targeting for the delaying the progression of DKD.

## Introduction

Diabetic kidney disease (DKD), a predominant and severe microvascular complication of diabetes mellitus, has become the leading cause of end-stage renal disease [1]. Tubulointerstitial fibrosis (TIF), characterized by abnormal accumulation of extracellular matrix (ECM), is a crucial pathological feature and inevitable consequence of progressive DKD [2, 3]. However, the underlying mechanism of TIF development in DKD remains incompletely understood. Elucidating the pathogenesis of TIF will provide a critical foundation for targeted therapeutic strategies.

Renal tubular epithelial cells (TECs), the most abundant cell type in the kidney, are central to regeneration versus renal fibrosis following injury. Accordingly, in response to injury, TECs undergo changes and function as inflammatory and fibrogenic cells, with the consequent production of various bioactive molecules that drive progression of kidney diseases [4–6]. Compelling evidence indicates that injured TEC is a key instigator of TIF in the setting of DKD. However, the exact mechanisms underpinning injured tubular cells-dependent initiation and progression of TIF require elucidation.

Transfer RNA (tRNA)-derived fragments (tRFs) are an emerging category of conserved small non-coding RNAs that derived from cleavage of mature tRNAs or tRNA precursors exposed to stress or injury [7]. A growing body of evidence indicated that tRFs play crucial roles in diverse molecular and biological processes, including cell apoptosis, immune response, and RNA synthesis [8–10]. Intriguingly, our previous study showed that abnormal expression of tRFs was associated with podocyte differentiation and injury [11, 12]. Critically, serum-derived tRFs exhibiting differential expression in DKD patients represented promising circulating biomarkers, supporting their utility in non-invasive disease monitoring [13]. Recently, our group also indicated that tRF3-IleAAT, a tRF produced by nucleases at tRNA-specific sites, was involved in the ECM synthesis during the development of glomerulosclerosis [14]. Thus, whether specific tRF expressed in TECs mediates TIF is a fundamental question for further study.

Recently, we identified the tRF‑1:30‑Gln‑CTG‑4 as a master regulator in primary mouse TECs-mediated ECM production through high-throughput sequencing [15]. However, the potential role of tRF‑1:30‑Gln‑CTG‑4 in DKD remains unknown. In the present study, we comprehensively delineated the pathological role and underlying mechanism of tRF‑1:30‑Gln‑CTG‑4 in TIF in the setting of DKD. Interestingly, we found that tubular tRF‑1:30‑Gln‑CTG‑4 expression was significantly downregulated in DKD mice and was strongly associated with TIF. The overexpression of tRF‑1:30‑Gln‑CTG‑4 markedly alleviated the decline of renal function and delayed TIF progression in DKD. Mechanistically, we demonstrated that tRF‑1:30‑Gln‑CTG‑4 acted as a chaperone-like regulator that sequestered FBXO7, disrupting its E3 ligase activity toward PINK1, a master regulator for mitophagy homeostasis. Taken together, these findings not only represent novel insights into the pathogenesis of TIF but also provide a promising therapeutic target for the delaying the progression of DKD.

## Materials and methods

### Cell culture

The human proximal TEC line HK-2 was purchased from the American Type Culture Collection (CRL-2190, RRID: CVCL_0302). They were recently authenticated using STR profiling and tested for mycoplasma contamination to ensure validity and purity. Cells were cultured in DMEM/F12 medium supplemented with 10% fetal bovine serum and 1% penicillin-streptomycin (Gibco; Thermo Fisher Scientific, Inc.) in a humidified atmosphere of 5% CO_2_ and 21% O_2_ at 37 °C. Cells were treated with high glucose (HG, 30 mM) or normal glucose (NG, 5.5 mM). D-Mannitol (5.5 mM glucose + 24.5 mM mannitol) was chosen as a control for osmolality. After 48 h of treatment, the cells were harvested for subsequent experiments.

### tRF‑1:30‑Gln‑CTG‑4 mimic transfection

HK-2 cells were seeded in 12‑well plates and grown to ~70% confluence (2 × 10^5^ cells per well) at 37 °C. The tRF‑1:30‑Gln‑CTG‑4 mimic (5’‑GGTTCCATGGTGTAATGGTGAGCACTCTGG‑3’; Guangzhou RiboBio Co., Ltd.) or negative control (tRF-NC mimic; 5’‑UUAUAGUCGUGGGAGCAGGGAUCGGCUUCU‑3’) were transfected into cells at a concentration of 30 nM with RiboFECT™ CP Transfection Reagent (Guangzhou RiboBio Co., Ltd.) for 6 h at 37 °C according to the manufacturer’s instructions. Then, the medium was replaced with complete DMEM and cells were stimulated with HG (30 mM) at 37 °C for 48 h.

### DKD animal model and adeno-associated virus (AAV) treatment

The study protocols were reviewed and approved by the Institutional Animal Care and Use Committee of Nanjing Medical University. Experiments were conducted using 7-week-old male C57BL/6J mice (20–22 g) treated with streptozotocin (STZ, Sigma-Aldrich, USA) as a DKD model and their age-matched male mice as a control (GemPharmatech Co., Ltd., Nanjing, China). After one-week adaptive feed, all mice were randomly divided into a control group and an experimental group. The experimental group mice were intraperitoneally injected with 50 mg/kg/d of STZ for 5 consecutive days. The control group received the same dose of citrate buffer injection. After STZ treatment for 4 weeks, DKD mice received a single injection by tail vein with AAV-tRF-NC, AAV-tRF‑1:30‑Gln‑CTG‑4, AAV-NC, or AAV-FBXO7 RNAi (1 × 10^12^v.g.) for 8 weeks according to the experimental aims (*n* = 7 per group). All mice were euthanized by intraperitoneal injection of barbital sodium (100 mg/kg) at 20 weeks of age. Blood, 24 h urine samples and renal parenchyma specimens were collected for further experiments.

### Quantitative real-time polymerase chain reaction (qRT‒PCR) analysis

Total RNA was extracted using TRIzol (Invitrogen; Thermo Fisher Scientific, Inc.). RNA concentration and purity were detected using a NanoDrop instrument (Thermo Fisher Scientific, Inc.). The level of mRNA was quantified with HiScript III RT SuperMix and ChamQ^™^ SYBR^®^ qPCR Master Mix (Vazyme Biotech Co., Ltd.) using the StepOne Real‑Time PCR System (Applied Biosystems; Thermo Fisher Scientific, Inc.) according to the manufacturer’s instructions. β-actin was used as an internal control for normalization. For tRFs, PCR amplification was performed using the Bulge‑Loop™ miRNA RT‑qPCR Primer Sets (one RT primer and a pair of PCR primers for each set), which were designed specifically for each tRF by Guangzhou RiboBio Co., Ltd. After adding forward primer and universal reverse primer, the reaction mixtures were incubated at 95 °C for 10 min, followed by 95 °C for 2 s, 60 °C for 20 s and 70 °C for 10 s, for 40 PCR cycles in the StepOne Real‑Time PCR System (Applied Biosystems; Thermo Fisher Scientific, Inc.). U6 small nuclear RNA was used as the internal reference. All tRF primers were designed specifically by Guangzhou RiboBio Co., Ltd. and all mRNA primers were obtained from Generay Biotech Co. Ltd. The mRNA and tRF forward primer sequences were listed in Supplementary Tables I and II. The relative expression levels were normalized to endogenous controls and were expressed as 2^−ΔΔCt^ [16].

### Western blot

Cultured cells and kidney tissues were harvested and lysed in RIPA lysis buffer and protein levels were detected by Western blot using established protocols [17]. Primary antibodies against Collagen I (Col I, 1:1000, ab138492, Abcam, UK, RRID: AB_2861258), α-SMA (1:1000, AF1032, Affinity Biosciences, MICH, USA, RRID: AB_2835329), Fibronectin (FN, 1:1000, ab2413, Abcam, RRID: AB_2262874), β-tubulin (1:1000, AF7011, Affinity Biosciences, RRID: AB_2827688), FBXO7 (1:1000, 10696-1-AP, Proteintech, USA, RRID: AB_2246820), PINK1 (1:1000, 23274-1-AP, Proteintech, RRID: AB_2879244), LC3 (1:1000, #12741, Cell Signaling Technology, RRID: AB_2617131), DRP1 (1:1000, ab184247, Abcam, RRID: AB_2895215), P62 (1:1000, ab109012, Abcam, RRID: AB_2810880), and TOMM20 (1:1000, sc-17764, Santa Cruz, CA, USA, RRID: AB_628381) were used. The gel area was analyzed using ImageJ software.

### Kidney histology and quantification

The method was performed as described in a previous study [18]. Four-micrometer-thick sections of paraffin-embedded renal tissue were used for Masson’s trichrome, periodic acid–Schiff (PAS), and Sirius red staining. Interstitial fibrosis was evaluated by assessing at least ten random tissue sections per mouse under Masson’s trichrome and Sirius red staining. Fibrosis, indicated by blue or red staining, was scored by three experienced observers who were blinded to the experimental conditions, and the average fibrosis scores were reported. For the tubular injury score, ten random tissue sections per mouse were assessed using PAS staining and scored semiquantitatively as follows: 0, no damage; 1, <25%; 2, 25–50%; 3, 50–75%; 4, >75%. The scores were averaged for each mouse.

### Immunofluorescence staining and Immunohistochemistry

Four-micrometer-thick paraffin-embedded renal tissue sections were prepared, and a streptavidin peroxidase detection system (Maixin, Fuzhou, China) was used for Immunofluorescence staining and Immunohistochemistry assays. All experimental steps followed the manufacturer’s protocol. Tissue sections were incubated with primary antibodies against Col I, FN, FBXO7, PINK1, LC3B (ab192890, Abcam, UK, RRID: AB_2827794) and P62. The quantitative process followed the blind method principle, and was conducted by three experienced researchers who were unaware of the group information, using ImageJ software to quantify signal intensity or the percentage of positive area. Immunostained samples were visualized under a fluorescence microscope.

### Fluorescence in situ hybridization (FISH)

FISH kit (GenePharma, Shanghai, China) was used to determine the location of tRF‑1:30‑Gln‑CTG‑4 in HK-2 cells and kidney tissues. Cy3-labeled probes of tRF‑1:30‑Gln‑CTG‑4 were obtained from GenePharma. Four-micrometer-thick paraffin-embedded tissue sections were digested with proteinase K and then incubated with blocking buffer for 30 min at 37 °C. Further, the tissue sections were hybridized with Cy3-labeled DNA probe sets at 37 °C overnight. FISH images were observed under a fluorescence microscope.

### Isolation of cytoplasmic and nuclear

HK-2 cells were separated into cytoplasmic and nuclear fractions according to the instructions of the Nuclear and Cytoplasmic Protein Extraction Kit (Beyotime Biotechnology, Shanghai, China). RNA was then extracted separately from each fraction using a TRIzol reagent. U6 was used as the internal reference for the nuclear fraction, and GAPDH was used as an internal reference for the cytoplasmic fraction.

### RNA pull-down

The pull-down analysis (Thermo Fisher Scientific, CA) was performed according to the manufacturer’s instructions. To form the secondary structure of RNA, 3 µg of biotin-labeled RNAs were added to the structure buffer, heated at 95 °C for 2 min, and placed on ice for 3 min. Magnetic beads were washed using 500 µL washing buffer three times, then added to biotin-labeled RNAs overnight. The next day, the mixture was added to the cell lysate with RNase inhibitor and incubated for 1 h. The mixture of beads and RNA was washed with washing buffer and boiled in SDS buffer. Then, the proteins were detected by Western blot.

### RNA immunoprecipitation (RIP)

RIP assay was performed using the RNA Immunoprecipitation Kit (Bes5101, BersinBio, Guangzhou, China). Anti-FBXO7 or anti-IgG was combined with magnetic beads and incubated with total RNA lysate of HK-2 cells. Subsequently, the protein-binding RNA was extracted and purified, and the expression level of tRF‑1:30‑Gln‑CTG‑4 was detected by qRT-PCR.

### Protein half-life assay

To determine the protein half-life of FBXO7, HK-2 cells were treated with protein synthesis inhibitor cycloheximide (CHX, 50 μg/mL) for the indicated time periods (0, 2, 4, and 6 hours), followed by immunoblotting analysis.

### Immunoprecipitation (IP) assay

IP assay was performed separately using anti-FBXO7 antibody or anti-PINK1 antibody. Each antibody was independently combined with Protein A/G PLUS-Agarose beads (Santa Cruz Biotechnology, TX, USA) and then incubated with HK-2 cell lysates.

### Transmission electron microscopy (TEM)

Collected kidney tissues were immersed in 2.5% glutaraldehyde. Sample handling and detection were performed by electron microscopic core lab at Nanjing Medical University.

### Mitochondrial ROS (MitoSOX)

Mitochondrial ROS production in HK-2 cells was measured using MitoSOX Kit (M36008, Invitrogen). Briefly, cells were harvested and incubated with 10 μM MitoSOX at 37 °C for 20 min. The fluorescence signals of mitochondrial ROS were measured using Image J software.

### Mitochondrial membrane potential (JC-1)

JC-1 in HK-2 cells was determined using JC-1 Kit (40706ES, Yeasen Biotechnology) according to the manufacturer’s protocol. The red-to-green fluorescence ratio was used to assess changes in JC-1. For fluorescence evaluation, the red and green fluorescence signals were transformed into average grayscale intensity values, which were subsequently analyzed using Image J software.

### Correlation analysis

To evaluate the potential correlation between tRF‑1:30‑Gln‑CTG‑4 and TIF, Pearson’s correlation analysis was applied. According to the classification for Pearson’s correlation coefficient (*r*), “*r* = 0” indicated “no correlation”; “*r* < 0.3” represented “poor correlation”; “0.3 ≤ *r* < 0.5” was treated as “moderate correlation”; “0.5 ≤ *r* < 0.7” was regarded as “good correlation”; “*r* ≥ 0.7” indicated “strong correlation” and “*r* = 1” represented “perfect correlation”.

### Statistical analysis

Data are expressed as the mean ± SEM. Statistical analyses were performed using SPSS 22.0. When two groups were compared, an unpaired Student’s *t* test was used. When three or more groups were compared, one-way ANOVA followed by Bonferroni’s correction was employed to analyze the differences. Statistical significance was set at *p* < 0.05.

## Results

### Downregulation of tRF‑1:30‑Gln‑CTG‑4 is associated with TIF in DKD

**Fig. 1:**
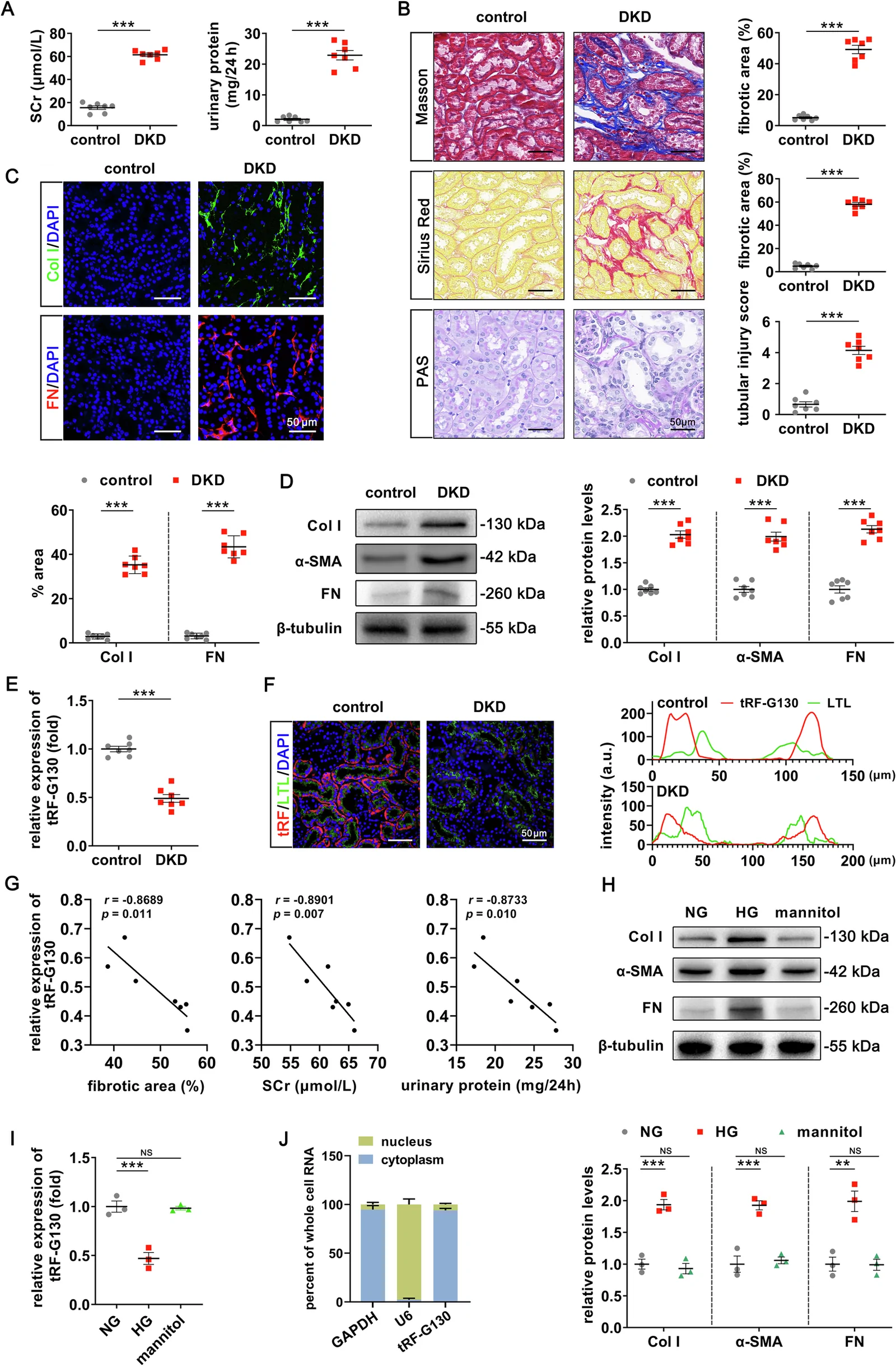
Downregulation of tRF‑1:30‑Gln‑CTG‑4 is associated with TIF in DKD. **A** The SCr and 24 h urinary protein in mice with DKD at 20 weeks of age. **B** Representative images of Masson, Sirius Red and PAS staining of the renal cortex. Scale bars, 50 μm. **C** Immunofluorescence staining analysis of Col I and FN expression in kidney tissues. Scale bars, 50 µm. **D** Western blot analysis of Col I, α-SMA and FN expression in kidney tissues. **E** qRT‒PCR analysis of tRF‑1:30‑Gln‑CTG‑4 expression in kidney tissues. **F** FISH analysis of tRF‑1:30‑Gln‑CTG‑4 in kidney tissues. Scale bars, 50 µm. **G** Correlation analysis between tRF‑1:30‑Gln‑CTG‑4 and TIF in mice with DKD. **H** Western blot analysis of Col I, α-SMA and FN expression in HG-treated HK-2 cells. Mannitol was used as the third group for balancing osmolality. **I** qRT‒PCR analysis of tRF‑1:30‑Gln‑CTG‑4 expression in HG-treated HK-2 cells. Mannitol was used as the third group for balancing osmolality. **J** Separation of nuclear and cytoplasmic fractions analysis of tRF‑1:30‑Gln‑CTG‑4 in HK-2 cells. *n* = 7 per group in vivo; *n* = 3 per group in vitro. ***p < 0.001; NS no significant. tRF‑1:30‑Gln‑CTG‑4 is abbreviated to tRF-G130.

The expression of tRF‑1:30‑Gln‑CTG‑4 in DKD mice was explored. First, STZ-induced DKD mouse model was successfully established, as evidenced by markedly elevated SCr and 24 h urinary protein (Fig. 1A) and renal histopathological changes (Fig. 1B). Intriguingly, significant tubular injury and interstitial fibrosis were observed in the DKD group (Fig. 1B). As shown in Fig. 1C, D and Supplementary Fig. S1A, the expression levels of the fibrotic markers Col I, α-SMA and FN were markedly increased in DKD mice as indicated by qRT-PCR, Western blot and Immunofluorescence staining analysis, highlighting TIF as a hallmark of DKD. Subsequently, qRT-PCR result revealed that compared with the control group, the tRF‑1:30‑Gln‑CTG‑4 expression was significantly decreased in DKD mice (Fig. 1E). Concomitantly, the tRF‑1:30‑Gln‑CTG‑4 expression was predominantly located in the lotus tetragonolobus lectin (LTL^+^) proximal tubules, as indicated by the FISH analysis (Fig. 1F). Interestingly, using Pearson’s correlation analysis, we found that there was a strong negative correlation between tRF‑1:30‑Gln‑CTG‑4 and both renal function and TIF (Fig. 1G), implying a potential link between tubular tRF‑1:30‑Gln‑CTG‑4 expression and renal TIF in DKD.

Moreover, the expression of tRF‑1:30‑Gln‑CTG‑4 in tubular cells was studied in vitro. HK-2 cells were exposed to HG to mimic diabetic conditions. Mannitol was used as the third group for balancing osmolality. As expected, the expression of tRF‑1:30‑Gln‑CTG‑4 was markedly decreased in HG-treated HK-2 cells, accompanied by increased expression of Col I, α-SMA and FN (Fig. 1H–I and Supplementary Fig. S1B). Consistent with the results in vivo, tRF‑1:30‑Gln‑CTG‑4 was predominantly expressed in the cytoplasm of HK-2 cells, a pattern that was recapitulated by separation of nuclear and cytoplasmic fractions (Fig. 1J). Together, these results indicate a significant decrease in tRF‑1:30‑Gln‑CTG‑4 expression in TECs, suggesting a potential involvement of tRF‑1:30‑Gln‑CTG‑4 in the pathogenesis of TIF under DKD conditions.

### tRF‑1:30‑Gln‑CTG‑4 overexpression ameliorates TIF in DKD

**Fig. 2:**
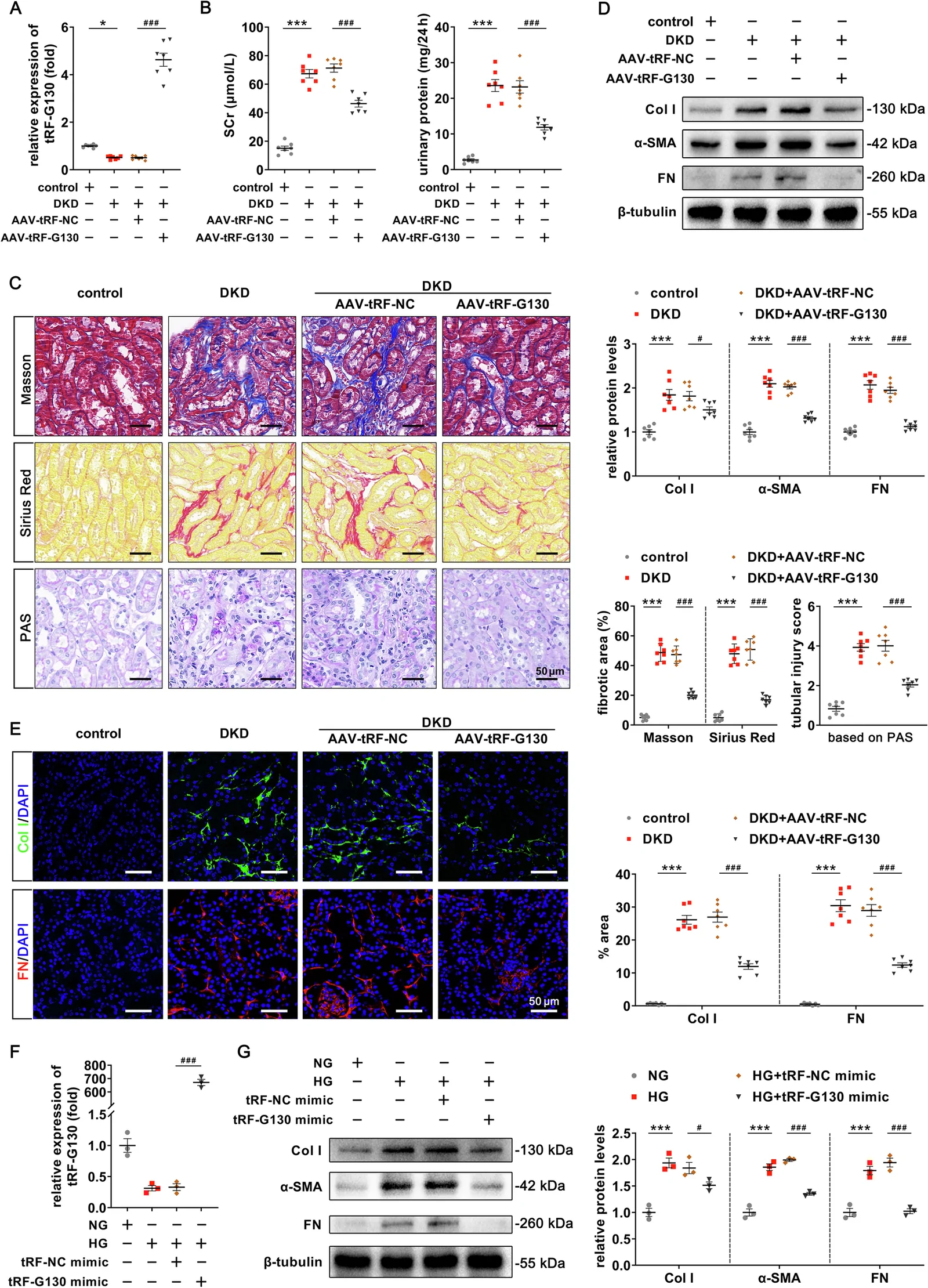
tRF‑1:30‑Gln‑CTG‑4 overexpression ameliorates TIF in DKD. **A** qRT‒PCR analysis of tRF‑1:30‑Gln‑CTG‑4 expression in kidney tissues. **B** The SCr and 24 h urinary protein in mice with DKD treated with AAV-tRF‑1:30‑Gln‑CTG‑4 at 20 weeks of age. **C** Representative images of Masson, Sirius Red and PAS staining and quantification of tubular injury and TIF. Scale bars, 50 μm. **D** Western blot analysis of Col I, α-SMA and FN expression in kidney tissues. **E** Immunofluorescence staining analysis of Col I and FN expression in kidney tissues. Scale bars, 50 μm. **F** qRT‒PCR analysis of tRF‑1:30‑Gln‑CTG‑4 expression in HK-2 cells. **G** Western blot analysis of Col I, α-SMA and FN expression in HK-2 cells. *n* = 7 per group in vivo; *n* = 3 per group in vitro. *p < 0.05, ***p < 0.001, ^#^*p* < 0.05, ^###^*p* < 0.001.

To further investigate the role of tRF‑1:30‑Gln‑CTG‑4 in TIF, the DKD mice at 12 weeks of age were intravenously administered AAV9 overexpressing tRF‑1:30‑Gln‑CTG‑4 (Supplementary Fig. S1C, D). The successful overexpression of tRF‑1:30‑Gln‑CTG‑4 in renal cortex was confirmed (Fig. 2A). Interestingly, compared with scrambled negative control-injected mice, the markedly elevated SCr and 24 h urinary protein were diminished in DKD mice treated with AAV-tRF‑1:30‑Gln‑CTG‑4 (Fig. 2B). The tubular injury and TIF were also ameliorated after tRF‑1:30‑Gln‑CTG‑4 overexpression, as evidenced by the Masson, Sirius Red and PAS staining (Fig. 2C). Consistently, qRT-PCR and Western blot analyses revealed that expressions of TIF-related proteins were significantly reversed in kidney with tRF‑1:30‑Gln‑CTG‑4 overexpression (Fig. 2D and Supplementary Fig. S1E). Immunofluorescence staining analysis further showed that tRF‑1:30‑Gln‑CTG‑4 overexpression could reduce Col I and FN accumulation in the renal interstitium (Fig. 2E). Therefore, tRF‑1:30‑Gln‑CTG‑4 plays a crucial role in attenuating TIF in DKD mice.

Furthermore, to directly investigate the effects of tRF‑1:30‑Gln‑CTG‑4 on TECs-mediated fibrogenesis under HG condition, we subjected HK-2 cells to HG and tRF‑1:30‑Gln‑CTG‑4 overexpression in vitro. The overexpression efficiency of tRF‑1:30‑Gln‑CTG‑4 in HK-2 cells was verified by qRT-PCR assay (Fig. 2F). Interestingly, qRT-PCR and Western blot analyses validated that tRF‑1:30‑Gln‑CTG‑4 overexpression could dramatically reduce the synthesis of Col I, α-SMA and FN (Fig. 2G and Supplementary Fig. S1F). These data collectively revealed that tRF‑1:30‑Gln‑CTG‑4 overexpression attenuates TIF in DKD.

### Downregulation of tRF‑1:30‑Gln‑CTG‑4 promotes TIF by elevating FBXO7 protein in DKD

**Fig. 3:**
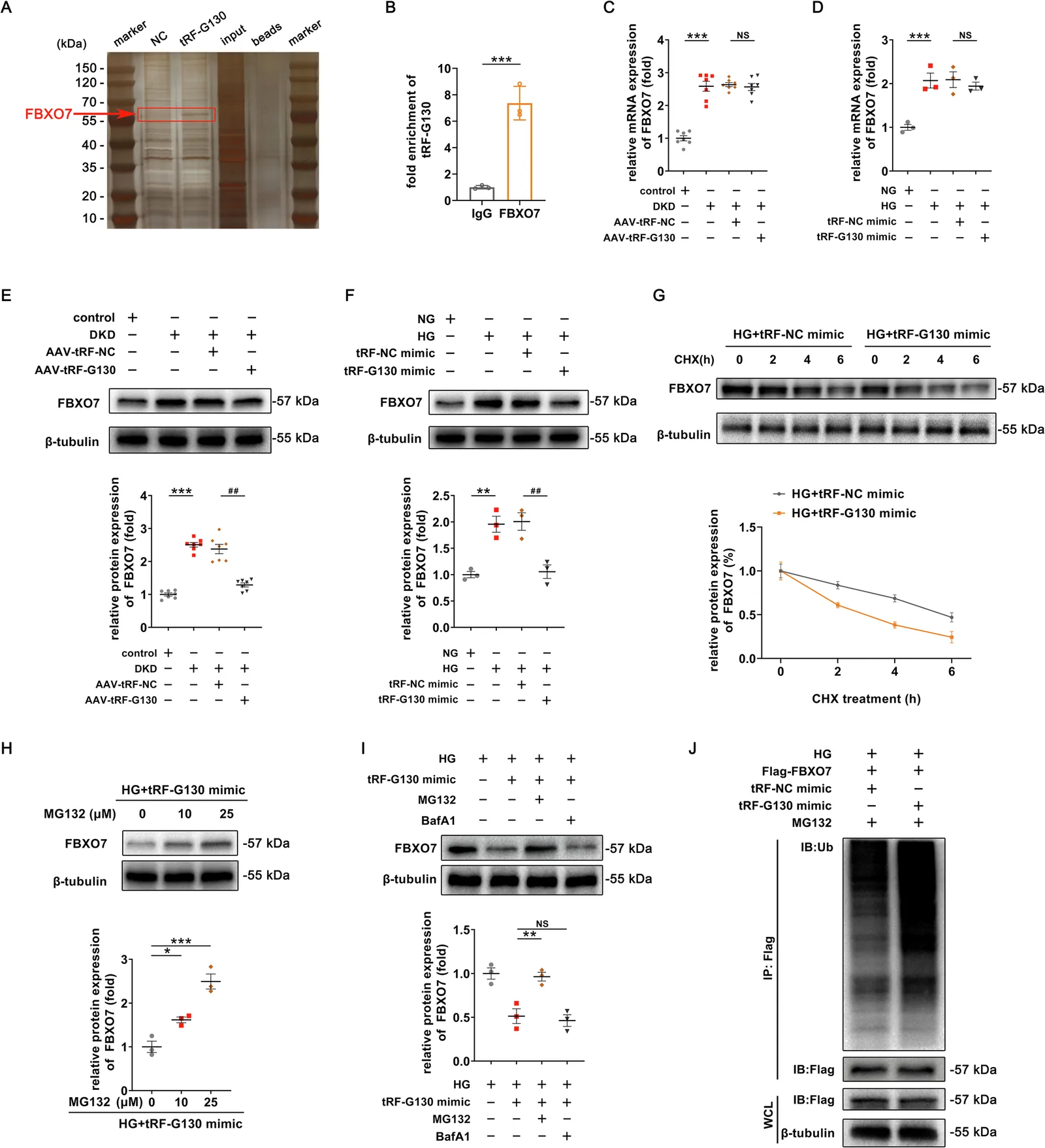
Downregulation of tRF‑1:30‑Gln‑CTG‑4 promotes TIF by elevating FBXO7 protein in DKD. **A** Pull-down assay was performed to detect the differential proteins obtained in HK-2 cells. **B** RIP assay was performed to detect the interaction of tRF‑1:30‑Gln‑CTG‑4 and FBXO7 in HK-2 cells. **C**, **E** qRT‒PCR and Western blot analyses of FBXO7 in DKD mice. **D**, **F** qRT‒PCR and Western blot analyses of FBXO7 in HK-2 cells. **G** Immunoblotting analysis of FBXO7 in HG-treated HK-2 cells with tRF‑1:30‑Gln‑CTG‑4 overexpression, treated with or without CHX for the indicated times. **H** Immunoblotting analysis of FBXO7 in tRF‑1:30‑Gln‑CTG‑4-overexpressing cells treated with or without MG132 (0, 10, 25 μM) for 6 h. **I** Immunoblotting analysis of FBXO7 in tRF‑1:30‑Gln‑CTG‑4-overexpressing cells treated with or without BafA1. **J** tRF‑1:30‑Gln‑CTG‑4 overexpression significantly promoted the ubiquitination of FBXO7. *n* = 7 per group in vivo; *n* = 3 per group in vitro. **p < 0.01, ^##^*p* < 0.01, ***p < 0.001, NS no significant.

Then, the exact mechanism by which tRF‑1:30‑Gln‑CTG‑4 regulated TIF was explored. Given that tRFs could exert their biological functions by interacting with proteins in the cytoplasm [7], we hypothesized that tRF‑1:30‑Gln‑CTG‑4 may play a crucial role in TIF by interacting with specific target proteins. Thus, RNA pull-down assay was performed. Interestingly, we found that compared with the antisense probe group, there was a distinct band in the 55–70 kDa position in the biotinylated tRF‑1:30‑Gln‑CTG‑4 probe group (Fig. 3A). Subsequently, LC-MS/MS analysis revealed that a total of 205 specific binding proteins were identified in the tRF‑1:30‑Gln‑CTG‑4 probe group (Supplementary material). Among these proteins, FBXO7, with a molecular weight of 57 kDa, particularly caught our attention due to its highest matching score. Further, the interaction of tRF‑1:30‑Gln‑CTG‑4 and FBXO7 was validated in HK-2 cells using RIP and Western blot analyses (Fig. 3B and Supplementary Fig. S2A), indicating that tRF‑1:30‑Gln‑CTG‑4 specifically bound to FBXO7.

Subsequently, the effect of tRF‑1:30‑Gln‑CTG‑4 overexpression on FBXO7 was studied in vivo and in vitro. Intriguingly, FBXO7 expression was found to be significantly up-regulated in DKD mice (Fig. 3C, E). Compared to the control group, overexpression of tRF‑1:30‑Gln‑CTG‑4 markedly reduced FBXO7 expression at the protein level in the DKD + AAV-tRF‑1:30‑Gln‑CTG‑4 group, not at transcriptional level (Fig. 3C, E). In parallel, the expression of FBXO7 in the HG-treated HK-2 cells followed a similar trajectory (Fig. 3D, F). Thus, we speculated that ubiquitin-proteasome degradation might be involved in the regulation of FBXO7 protein level by tRF‑1:30‑Gln‑CTG‑4. To verify the hypothesis, CHX was used to evaluate the protein stability of FBXO7. The result showed that tRF‑1:30‑Gln‑CTG‑4 overexpression significantly shortened the half-life of FBXO7 protein after CHX treatment (Fig. 3G). Subsequently, immunoblotting analysis revealed that the inhibitory effect of tRF‑1:30‑Gln‑CTG‑4 overexpression on FBXO7 protein level was markedly abolished by MG132 treatment (Fig. 3H). However, BafA1 intervention did not reverse the elimination of FBXO7 by tRF‑1:30‑Gln‑CTG‑4 overexpression (Fig. 3I). Ubiquitination experiment confirmed that tRF‑1:30‑Gln‑CTG‑4 overexpression could promote FBXO7 ubiquitin proteasome degradation (Fig. 3J).

Together, above results confirm that tRF‑1:30‑Gln‑CTG‑4 overexpression downregulates FBXO7 protein level probably due to ubiquitin-proteasome degradation. These data indicate that tRF‑1:30‑Gln‑CTG‑4 plays a vital role in the TIF by directly binding to FBXO7 protein under the condition of DKD.

### Inhibition of FBXO7 ameliorates TIF in DKD

**Fig. 4:**
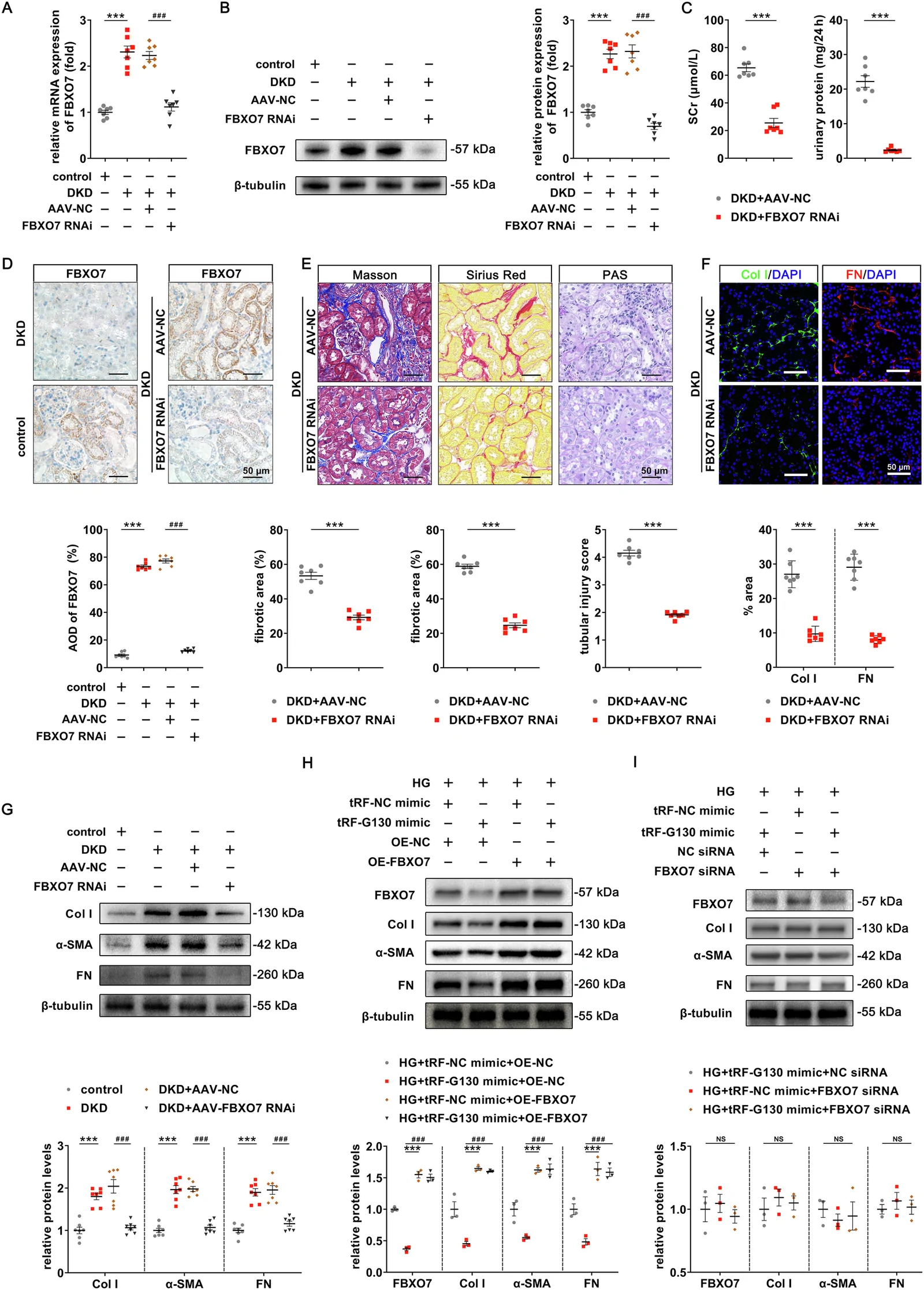
Inhibition of FBXO7 ameliorates TIF in DKD. **A**, **B** qRT‒PCR and Western blot analysis of FBXO7 expression in kidney tissues. **C** The SCr and 24 h urinary protein in mice with DKD treated with AAV-FBXO7 RNAi at 20 weeks of age. **D** Immunohistochemistry analysis of FBXO7 expression in kidney tissues. Scale bars, 50 μm. **E** Representative images of Masson, Sirius Red and PAS staining and quantification of tubular injury and TIF. Scale bars, 50 μm. **F** Immunofluorescence staining analysis of Col I and FN expression in kidney tissues. Scale bars, 50 µm. **G**–**I** Western blot analysis of FBXO7, Col I, α-SMA and FN expression in kidney tissues. *n* = 7 per group in vivo. ***p < 0.001, ^###^*p* < 0.001, NS no significant.

To determine the role of FBXO7 in TIF, AAV-FBXO7 RNAi or AAV-NC was administered via the tail vein of DKD mice (Supplementary Fig. S2B, C). As expected, FBXO7 expression was significantly decreased in the DKD + AAV-FBXO7 RNAi group, as evidenced by the results of qRT-PCR, Western blot and Immunohistochemistry analyses (Fig. 4A, B, D). Interestingly, inhibition of FBXO7 effectively reversed the elevated SCr and 24 h urinary protein levels in DKD mice (Fig. 4C). In addition, tubule injury and TIF were markedly diminished in the kidney of the AAV-FBXO7 RNAi-injected mice with DKD, as evidenced by Masson, Sirius Red and PAS staining (Fig. 4E). Moreover, Immunofluorescence staining revealed that the downregulation of FBXO7 significantly reduced Col I and FN expression (Fig. 4F). Meanwhile, knockdown of FBXO7 similarly reversed the mRNA and protein expression of Col I, α-SMA and FN (Fig. 4G and Supplementary Fig. S2D).

Next, FBXO7 siRNA was transfected in HG-treated HK-2 cells to directly explore the role of FBXO7 in tubular cells. As shown in Supplementary Fig. S2E, F, the knockdown efficiency of FBXO7 was verified by qRT-PCR and Western blot. Consistent with the results in vivo, FBXO7 downregulation reduced the expression of Col I, α-SMA and FN in HG + FBXO7 siRNA group compared with that in HG + NC siRNA group (Supplementary Fig. S2E, 2F). Subsequently, FBXO7 protein was restored in HG + tRF‑1:30‑Gln‑CTG‑4 mimic + OE-FBXO7 group. The result showed that Col I, α-SMA and FN expression levels were markedly increased compared with HG + tRF‑1:30‑Gln‑CTG‑4 mimic + OE-NC group (Fig. 4H). there was no significant difference in the expression of FBXO7, Col I, α-SMA, and FN between cells transfected with FBXO7 siRNA or tRF‑1:30‑Gln‑CTG‑4 mimic alone and those co-transfected with both FBXO7 siRNA and tRF‑1:30‑Gln‑CTG‑4 mimic (Fig. 4I).

Together, these data revealed that inhibition of FBXO7 dramatically ameliorates the TIF in DKD.

### FBXO7 enhances TIF by ubiquitylation of PINK1

**Fig. 5:**
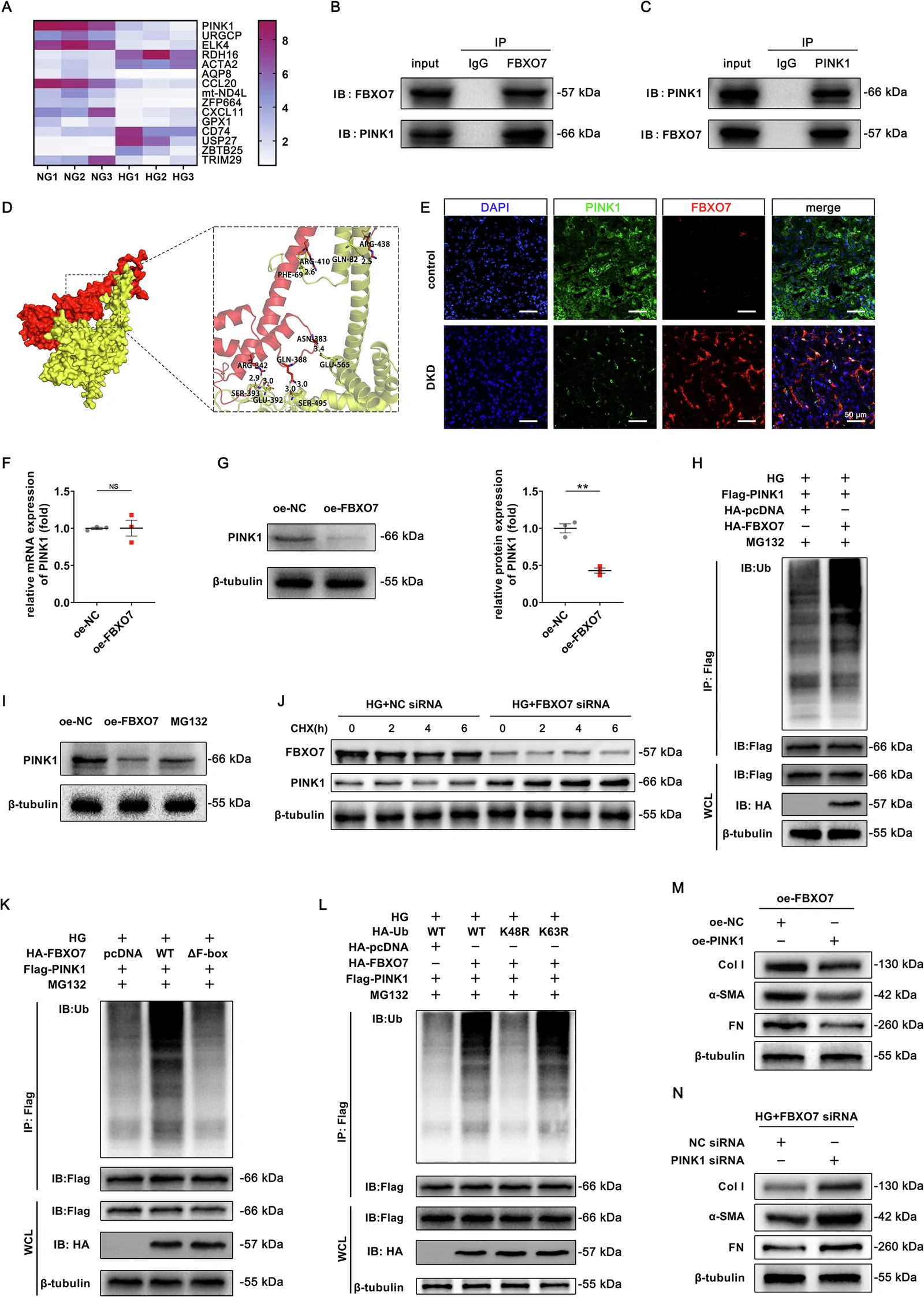
FBXO7 enhances TIF by ubiquitylation of PINK1. **A** The hierarchical clustering heatmap shows differentially expressed proteins interacting FBXO7 among groups determined by IP-MS. **B** IP of protein isolated from HK-2 cells with an antibody against FBXO7 followed by IB analyses with a PINK1 antibody showed the interaction of FBXO7 and PINK1 in cells exposed to HG. Rabbit IgG served as the control (*n* = 3). **C** IP of protein isolated from HK-2 cells with an antibody against PINK1 followed by IB analyses with a FBXO7 antibody showed the interaction of FBXO7 and PINK1 in cells exposed to HG. Rabbit IgG served as the control (*n* = 3). **D** The binding site and mode of FBXO7 (red) - PINK1 (yellow) complex. **E** The expression of FBXO7 (red) and PINK1 (green) was determined by double staining in kidney sections from mice with DKD. (*n* = 7; scale bar, 50 μm). **F**, **G** qRT‒PCR and Western blot analysis of PINK1 in HK-2 cells after FBXO7 overexpression. **H** FBXO7 overexpression significantly promoted the ubiquitination of PINK1. **I** Western blot analysis of PINK1 expression in HK-2 cells pretreated with MG132 after FBXO7 overexpression. **J** The half-life of PINK1 was detected in HK-2 cells treated with FBXO7 siRNA and CHX at the indicated time points. **K** PINK1, WT or ΔF-box mutant of FBXO7 were co-expressed in HG-treated HK-2 cells. **L** The lysine residues to arginine (K48R, K63R) were mutated and performed in vitro ubiquitination assay. **M** Western blot analysis of Col I, α-SMA and FN expression after FBXO7 overexpression and PINK1 overexpression (*n* = 3). **N** Western blot analysis of Col I, α-SMA and FN expression after FBXO7 and PINK1 knockdown in HG-treated HK-2 cells (*n* = 3). **p < 0.01, NS no significant.

Then, the exact mechanism by which FBXO7 promoted TIF in DKD was further investigated. Previous studies reported that FBXO7, belonging to the F-box proteins, plays a crucial role in recognizing and recruiting specific substrates as a ubiquitin E3 ligase for ubiquitylation and subsequent degradation [19]. Therefore, to identify the potential substrate of FBXO7 in TECs, we performed IP assay combined with LC-MS/MS using HK-2 cells treated with HG. Among the top 15 FBXO7-binding proteins identified by the quantitative LC-MS/MS analysis, PINK1 emerged as the top candidate due to its highest matching score (Fig. 5A). We hypothesized that FBXO7 may be involved in the TIF in DKD through ubiquitinating PINK1.

As shown in Fig. 5B, when HK-2 cells lysates were immunoprecipitated with anti-FBXO7 antibody, PINK1 was detected but was not found in the precipitates by normal control IgG. Meanwhile, when HK-2 cells lysates were immunoprecipitated with anti-PINK1 antibody, FBXO7 was also detected (Fig. 5C). Besides, the structure of FBXO7 and PINK1 was analyzed, and their interaction sites were identified by molecular docking (Fig. 5D). Subsequently, the co-localization of FBXO7 and PINK1 was further confirmed in DKD mice (Fig. 5E), indicating that FBXO7 interacts with PINK1 in DKD mice. Furthermore, we found that the overexpression of FBXO7 significantly reduced PINK1 protein expression without affecting the mRNA level in HK-2 cells (Fig. 5F, G). Moreover, we explored the potential role of FBXO7 in PINK1. Interestingly, ubiquitylation assay revealed that FBXO7 enhanced PINK1 ubiquitylation (Fig. 5H–J). Subsequently, we constructed wild-type (WT) and ΔF-box mutant (enzymatically dead mutant) of FBXO7 in vitro. Then, we co-expressed PINK1, WT or ΔF-box mutant of FBXO7 in HG-treated HK-2 cells. The result demonstrated that FBXO7 WT, but not its enzymatically dead ΔF-box mutant, prominently promoted the ubiquitination of PINK1 (Fig. 5K). Seo JJ et al. reported that the regulation of PINK1 by F-box family protein, FBW7β, was mediated by K48 chain mediated proteasomal degradation [20]. In addition, UXT-V2 was polyubiquitinated by FBXO7 with K48 and K63 ubiquitin chain linkages in vitro and in vivo. We speculate that FBXO7 facilitates the K48 or K63-linked polyubiquitination of PINK1, targeting it for degradation. Then we mutated the lysine residues to arginine (K48R, K63R) and performed in vitro ubiquitination assay. As shown in Fig. 5L, obvious increase in PINK1 ubiquitination could be observed in K63R mutant of PINK1, but not in K48R mutant. To sum up, FBXO7 mediates PINK1 ubiquitination at lysine 48 in HK-2 cells.

Finally, the role of FBXO7/PINK1 axis in TIF was investigated. As shown in Fig. 5M and Supplementary Fig. S3A, B, the expression levels of Col I, α-SMA and FN were increased in HK-2 cells with FBXO7 overexpression, while this trend was reversed by PINK1 overexpression, as indicated by qRT-PCR and Western blot analysis. Meanwhile, the expression of Col I, α-SMA and FN were markedly decreased in HG + FBXO7 siRNA group, whereas the opposite results were observed after PINK1 knockdown (Fig. 5N and Supplementary Fig. S3A, B). These results demonstrated that the ubiquitination modification of PINK1 regulated by FBXO7 plays a key role in TIF under the condition of DKD.

### FBXO7-mediated ubiquitination of PINK1 inhibited mitophagy

**Fig. 6:**
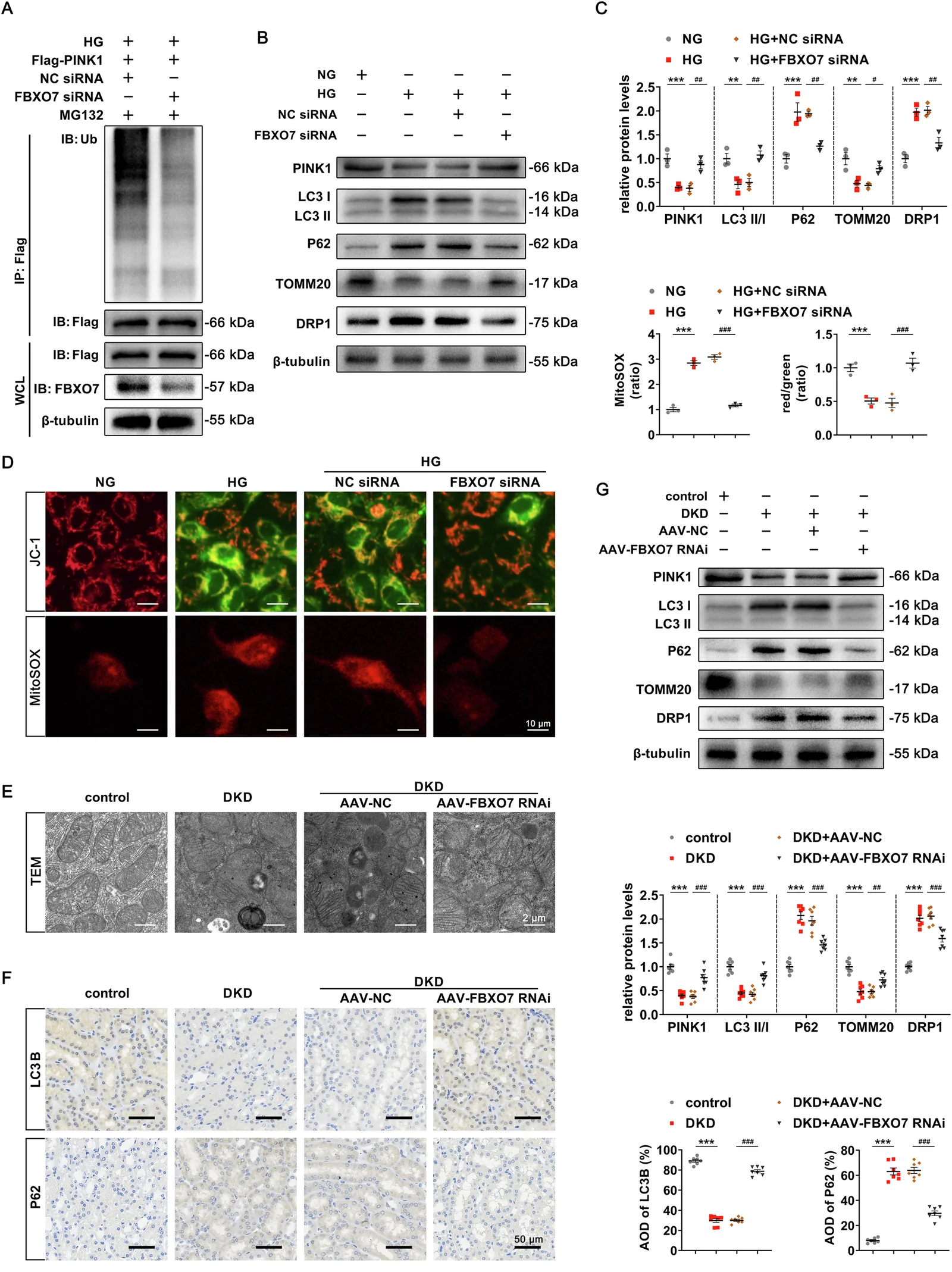
FBXO7-mediated ubiquitination of PINK1 inhibited mitophagy. **A** Knockdown of FBXO7 significantly inhibited the ubiquitination of PINK1. **B**, **C** Western blot and qRT‒PCR analysis of PINK1, LC3 II/I, P62, TOMM20 and DRP1 in HK-2 cells after knockdown of FBXO7. **D** JC-1 probe was used to evaluate mitochondrial potential. Red-to-green fluorescence ratio was used to quantify mitochondrial potential. MitoSOX Red staining represented mitochondrial ROS levels in HK-2 cells. Scale bar, 10 μm. The fluorescence signals of mitochondrial ROS were measured using Image J software. **E** Representative TEM images of mitophagosomes in kidney tissues from DKD mice administrated with AAV-FBXO7 RNAi. Scale bar, 2 μm. **F** Immunohistochemical staining analysis of LC3B and P62 after AAV-FBXO7 RNAi treatment in DKD mice. Scale bar, 50 μm. **G** Western blot analysis of PINK1, LC3 II/I, P62, TOMM20 and DRP1 in DKD mice treated with AAV-FBXO7 RNAi. *n* = 7 per group in vivo; *n* = 3 per group in vitro. **p < 0.01, ***p < 0.001, ^#^*p* < 0.05, ^###^*p* < 0.001, NS no significant.

Given the role of PINK1 in mitophagy [21], FBXO7-mediated ubiquitination of PINK1 may be involved in TIF by inhibiting mitophagy. As shown in Fig. 6A, knockdown of FBXO7 in HG-treated HK-2 cells attenuated its ability to promote ubiquitination-dependent degradation of PINK1. Indeed, downregulation of PINK1 in HG-treated HK-2 cells was significantly reversed by FBXO7 knockdown, accompanied by an increase in the expression of LC3 II/I and TOMM20, and a decrease in the expression of P62 and DRP1 (Fig. 6B, C and Supplementary Fig. S3C). Furthermore, MitoSOX and JC-1 staining results showed that FBXO7 knockdown maintained mitochondrial membrane potential, but decreased mitochondrial ROS levels in HG-treated HK-2 cells (Fig. 6D).

In vivo, TEM analysis showed that there were obvious damaged mitochondria in TECs from DKD mice, as indicated by mitochondrial swelling, disappeared cristae and rupture of the mitochondrial membrane, which were attenuated by FBXO7 knockdown in DKD mice (Fig. 6E). Moreover, integrated analyses by qRT-PCR, Western blot and Immunohistochemistry staining revealed that PINK1, LC3 II/I and TOMM20 expression were markedly downregulated, whereas P62 and DRP1 were upregulated in DKD mice. These alterations were reversed upon FBXO7 knockdown (Fig. 6F, G and Supplementary Fig. S3C). Therefore, these results reveal that FBXO7-mediated ubiquitination of PINK1 inhibits PINK1-mediated mitophagy.

### Overexpression of tRF‑1:30‑Gln‑CTG‑4 alleviated disturbed mitophagy

**Fig. 7:**
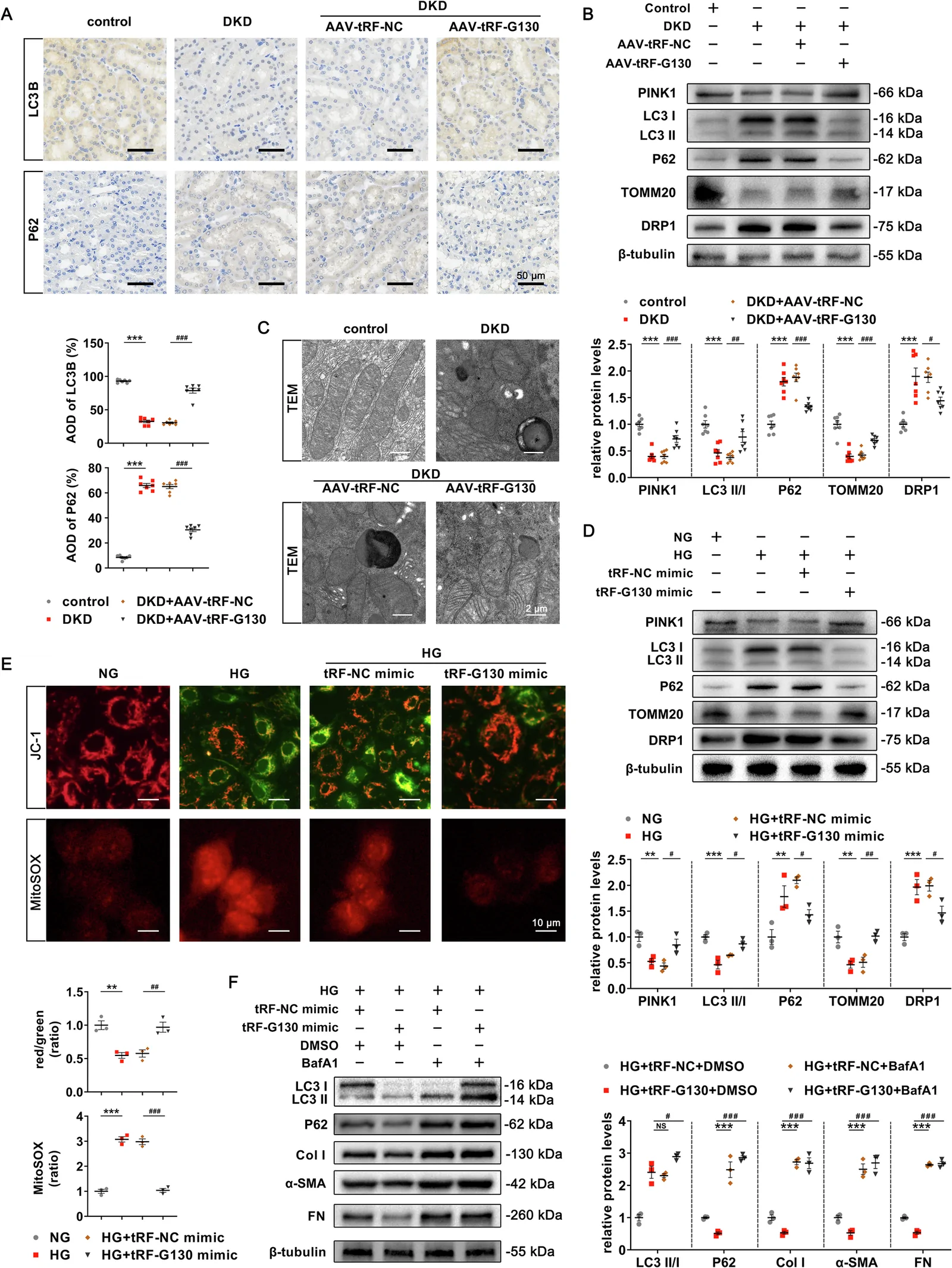
Overexpression of tRF‑1:30‑Gln‑CTG‑4 alleviated disturbed mitophagy. **A** Immunohistochemical staining analysis of LC3B and P62 after AAV-tRF-G130 treatment in DKD mice. Scale bar, 50 μm. **B** Western blot analysis of PINK1, LC3 II/I, P62, TOMM20 and DRP1 in DKD mice treated with AAV-tRF-G130. **C** Representative TEM images of mitophagosomes in kidney tissues from DKD mice administrated with AAV-tRF-G130. Scale bar, 2 μm. **D** Western blot analysis of PINK1, LC3 II/I, P62, TOMM20 and DRP1 in HK-2 cells. **E** JC-1 probe was used to evaluate mitochondrial potential. Red-to-green fluorescence ratio was used to quantify mitochondrial potential. MitoSOX Red staining represented mitochondrial ROS levels in HK-2 cells. Scale bar, 10 μm. The fluorescence signals of mitochondrial ROS were measured using Image J software. **F** Western blot analysis of PINK1, LC3 II/I, P62, TOMM20 and DRP1 in HK-2 cells treated with tRF-G130 mimic after BafA1 treatment. *n* = 7 per group in vivo; *n* = 3 per group in vitro. **p < 0.01, ***p < 0.001, ^###^*p* < 0.001, NS no significant. tRF‑1:30‑Gln‑CTG‑4 is abbreviated to tRF-G130.

Finally, to investigate the functional effects of tRF‑1:30‑Gln‑CTG‑4 on tubular mitophagy in vivo, AAV9 injection via the tail vein was performed to overexpress tRF‑1:30‑Gln‑CTG‑4 in DKD mice. tRF‑1:30‑Gln‑CTG‑4 overexpression increased the expression of PINK1, LC3 II/I and TOMM20, and decreased P62 and DRP1 expression (Fig. 7A, B and Supplementary Fig. S3D). TEM result showed that tRF‑1:30‑Gln‑CTG‑4 overexpression alleviated abnormal mitochondria (Fig. 7C). Moreover, after tRF‑1:30‑Gln‑CTG‑4 mimic treatment, there was a notable increase of PINK1, LC3 II/I and TOMM20 expression, accompanied by a decrease in the expression of P62 and DRP1 (Fig. 7D and Supplementary Fig. S3D). Furthermore, tRF‑1:30‑Gln‑CTG‑4 overexpression reversed mitochondrial dysfunction in HG-treated HK-2 cells, as determined by the increase in the mitochondrial membrane potential level and reduction in mitochondrial ROS production (Fig. 7E). We treated HK-2 cells with the autophagy inhibitor BafA1 under conditions of tRF‑1:30‑Gln‑CTG‑4 overexpression. The result showed that BafA1 intervention reversed the protective effect of tRF‑1:30‑Gln‑CTG‑4 on autophagic flux (Fig. 7F). Therefore, overexpression of tRF‑1:30‑Gln‑CTG‑4 enhances mitophagy in DKD.

## Discussion

**Fig. 8:**
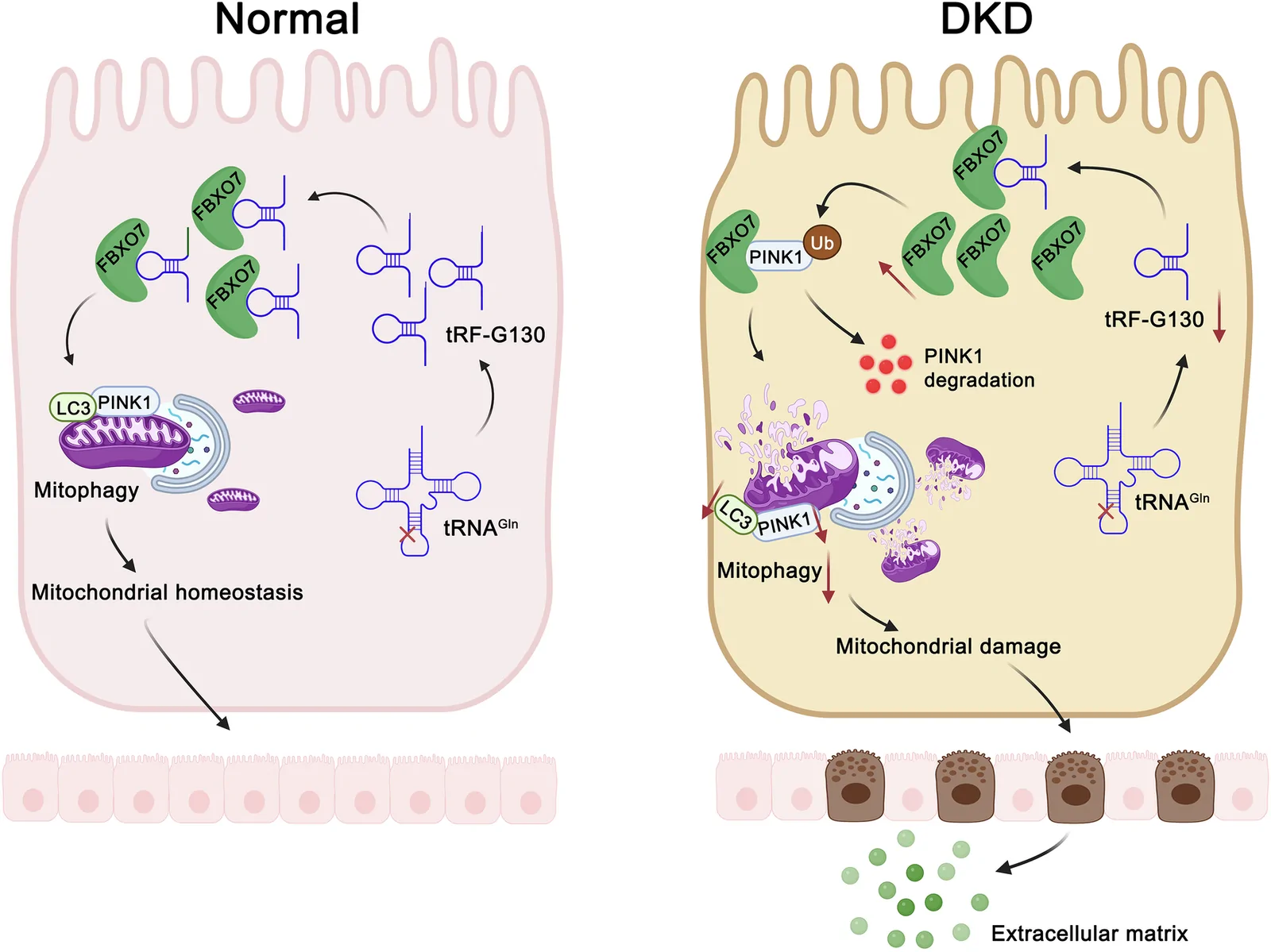
Schematic illustration of the mechanism by which tRF‑1:30‑Gln‑CTG‑4 deficiency could cause TIF in DKD. The tubular tRF‑1:30‑Gln‑CTG‑4 was markedly decreased and strongly correlated with TIF in DKD. Mechanistically, tRF‑1:30‑Gln‑CTG‑4 acted as a chaperone-like regulator that sequestered FBXO7, disrupting its E3 ligase activity toward PINK1, a master regulator for mitophagy homeostasis.

tRF, as an emerging key regulator, represents a novel small non-coding RNA class with various biologic functions [22–24]. However, the roles of tRF in TIF in the setting of DKD remain poorly understood. In the present study, the functional role of a specific tRF in diabetic TIF was comprehensively explored. Interestingly, we found that the tubular tRF‑1:30‑Gln‑CTG‑4 was markedly decreased and strongly correlated with TIF in DKD mice. Mechanistically, tRF‑1:30‑Gln‑CTG‑4 acted as a chaperone-like regulator that sequestered FBXO7, disrupting its E3 ligase activity toward PINK1, a master regulator for mitophagy homeostasis. Thus, a previously unrecognized mechanism linking tubular tRF to TIF in DKD was demonstrated (Fig. 8). Our findings may provide a promising therapeutic target for delaying the progression of DKD.

While emerging mechanisms of DKD have been identified, clinically viable therapeutic strategies for preventing DKD are still lacking. TIF is the final common outcome of almost all progressive chronic kidney diseases. Deciphering its underlying mechanisms is essential for establishing novel therapeutic strategies to prevent or delay progression of DKD. Methodological advances have enabled the characterization of a growing repertoire of novel non-coding RNAs, among which tRFs represent an abundant class of small non-coding RNAs whose biological roles are not well understood. Recently, Lu et al. [25] reported that tRF-3022b was involved in colorectal tumor growth and M2 macrophage polarization via binding to galectin 1 and macrophage migration inhibitory factor. Meanwhile, a novel tRF3-Val was found to promote gastric cancer proliferation by targeting EEF1A1 [26]. However, the potential effects of tRFs on kidney diseases remain fragmentary. In the present study, we for the first time comprehensively delineated the pathological role of tRF‑1:30‑Gln‑CTG‑4 in the development of TIF in DKD. We found that tRF‑1:30‑Gln‑CTG‑4 was predominantly expressed in proximal tubular cells and was significantly downregulated in DKD mice. Then, we found that there was a strong correlation between tRF‑1:30‑Gln‑CTG‑4 and TIF, suggesting that tRF‑1:30‑Gln‑CTG‑4 may be a potential biomarker to identify TIF progression in DKD. Subsequently, the biological functions of tRF‑1:30‑Gln‑CTG‑4 were systematically and comprehensively explored. Interestingly, overexpression of tRF‑1:30‑Gln‑CTG‑4 could reverse the decline of renal function and ameliorate TIF in DKD, which indicated that tRF‑1:30‑Gln‑CTG‑4 may represent a novel therapeutic target for DKD.

Previous study showed that tRF‑1:30‑Gln‑CTG‑4 belonged to tRF-5c, a type of tRF-5, beginning at the 5′end of mature tRNAs and was cleaved by Dicer at the loop or the stem position between the D-loop and the anticodon loop [27]. Although the exact mechanism remains poorly understood, some studies have reported that this type of tRFs plays a crucial role in cellular biological processes by directly binding to downstream proteins in the cytoplasm, modulating post-translational modifications, or disrupting protein-protein interactions [28–30]. In our study, the exact mechanism by which tRF‑1:30‑Gln‑CTG‑4 regulated TIF was investigated. Interestingly, we identified that FBXO7 was one of the specific targets of tRF‑1:30‑Gln‑CTG‑4. Upon tRF‑1:30‑Gln‑CTG‑4 overexpression, the protein expression of FBXO7 was markedly decreased, which was consistent with previous studies, whereby binding to target protein reduces its protein expression level by affecting protein degradation pathways not their mRNA levels [31]. We found that overexpression of tRF-1:30‑Gln‑CTG‑4 could downregulate FBXO7 protein level probably due to ubiquitin-proteasome degradation. Thus, tRFs may function as structural small molecules that target diverse macromolecules. Collectively, a novel mechanism regulating signaling pathways in TIF has been identified.

FBXO7, an adaptor protein in the SKP1-Cullin-1-F-box E3 ubiquitin ligase complex, plays a crucial role in recognizing targeted substrates. A growing body of evidence indicated that FBXO7 was also involved in the regulation of the proteasome complex, the cell cycle, and cell proliferation [32–34], suggesting that FBXO7 may contribute to the pathogenesis of fibrotic diseases. Here, to elucidate the precise mechanism by which FBXO7 modulates TIF, co-immunoprecipitation coupled to mass spectrometry assay was used. Interestingly, we identified PINK1 as the top candidate target for FBXO7 in DKD. Further experiments confirmed that FBXO7 interacted with PINK1 each other and co-localized remarkably in the cytoplasm of renal TECs. Additionally, the increase in FBXO7-mediated K48 ubiquitination and proteasomal degradation of PINK1 contributes to the reduced PINK1 protein level in HG-treated HK-2 cells, which is consistent with the role of FBXO7 as an E3 ubiquitin ligase requiring both its substrate-recognition domain for binding and its F-box domain for recruiting the degradation machinery. Therefore, our study identified for the first time a novel regulatory mechanism mediated by FBXO7 through post-translational modification that regulates TIF progression in DKD.

Tubular mitochondrial dysfunction is not only a well-established feature of DKD, but also a crucial contributor to the pathogenesis of DKD [35]. Mitophagy is an important mitochondrial quality control mechanism that eliminates damaged mitochondria, which maintains cellular energy metabolism and physiological functions [36]. A growing body of evidence indicated that PINK1 was one of the master regulators in mitophagy. Previous studies have shown that PINK1 activated Parkin through phosphorylation, which is considered the primary regulatory mechanism of mitophagy [37, 38]. However, our study identified a novel mechanism of mitophagy regulation through the FBXO7-PINK1 protein degradation system. Accordingly, our in vitro and in vivo studies revealed that FBXO7 regulated mitophagy by ubiquitylation of PINK1. Furthermore, inhibiting FBXO7 expression not only suppressed ubiquitin proteasome degradation of PINK1, but also improved disturbed mitophagy. More interestingly, we found that overexpressing tRF‑1:30‑Gln‑CTG‑4 ameliorated damaged mitochondria and restored mitophagy homeostasis. Thus, a previously unrecognized pathway regulating mitophagy is identified, at least in the setting of DKD.

Accumulating evidence indicates that epigenetics (e.g. histone modifications and non-coding RNAs) play essential roles in multiple human diseases, including kidney diseases [39, 40]. Recently, Li et al. reported that a hypoxia-induced transfer RNA-derived small RNA sequestered pseudouridine synthase PUS7, preventing catalytic pseudouridylation of histone mRNAs, which plays a vital role in renal protection [30]. Understanding epigenetic changes during the early stages of DKD could be valuable for timely diagnosis and treatment. Therefore, it is of crucial importance to precisely elucidate the role and mechanisms of epigenetics in TIF under the condition of DKD. Here, we focused on the functional effects of the tRF on DKD and further elucidated its specific mechanisms. Impressively, a previously unrecognized mechanism regulating the progression of DKD has been demonstrated. Moreover, given that advances in gene therapy position non-coding RNA targeting as a promising therapeutic strategy, our findings may provide additional theoretical foundation for the treatment of DKD.

In conclusion, an important role of tubular tRF‑1:30‑Gln‑CTG‑4 in regulating TIF under the conditions of DKD is demonstrated. Accordingly, tRF‑1:30‑Gln‑CTG‑4 acted as a chaperone-like regulator that sequestered FBXO7, disrupting its E3 ligase activity toward PINK1, a master regulator for mitophagy homeostasis. Our findings not only represent a novel insight into the pathogenesis of TIF but also provide a promising therapeutic target for delaying the progression of DKD.

## Supplementary information

Supplementary figures and tables

Supplementary material

Raw data and uncropped western blot images

## Data Availability

The authors confirm that the data supporting the conclusions in the paper are presented in the article. Additional data related to this paper are available from the corresponding author.
