# Supplementary material for: Tubular tRF‑1:30‑Gln‑CTG‑4 attenuates tubulointerstitial fibrosis by mitigating FBXO7-mediated mitophagy defect in diabetic kidney disease
Source: Cell Death Dis. 2026 Jul 18;17(1):886. doi: 10.1038/s41419-026-09112-2 (PMC13661522; doi:10.1038/s41419-026-09112-2)
Supplement: Supplementary file 1 [file 41419_2026_9112_MOESM3_ESM.pdf]

Figure.1D

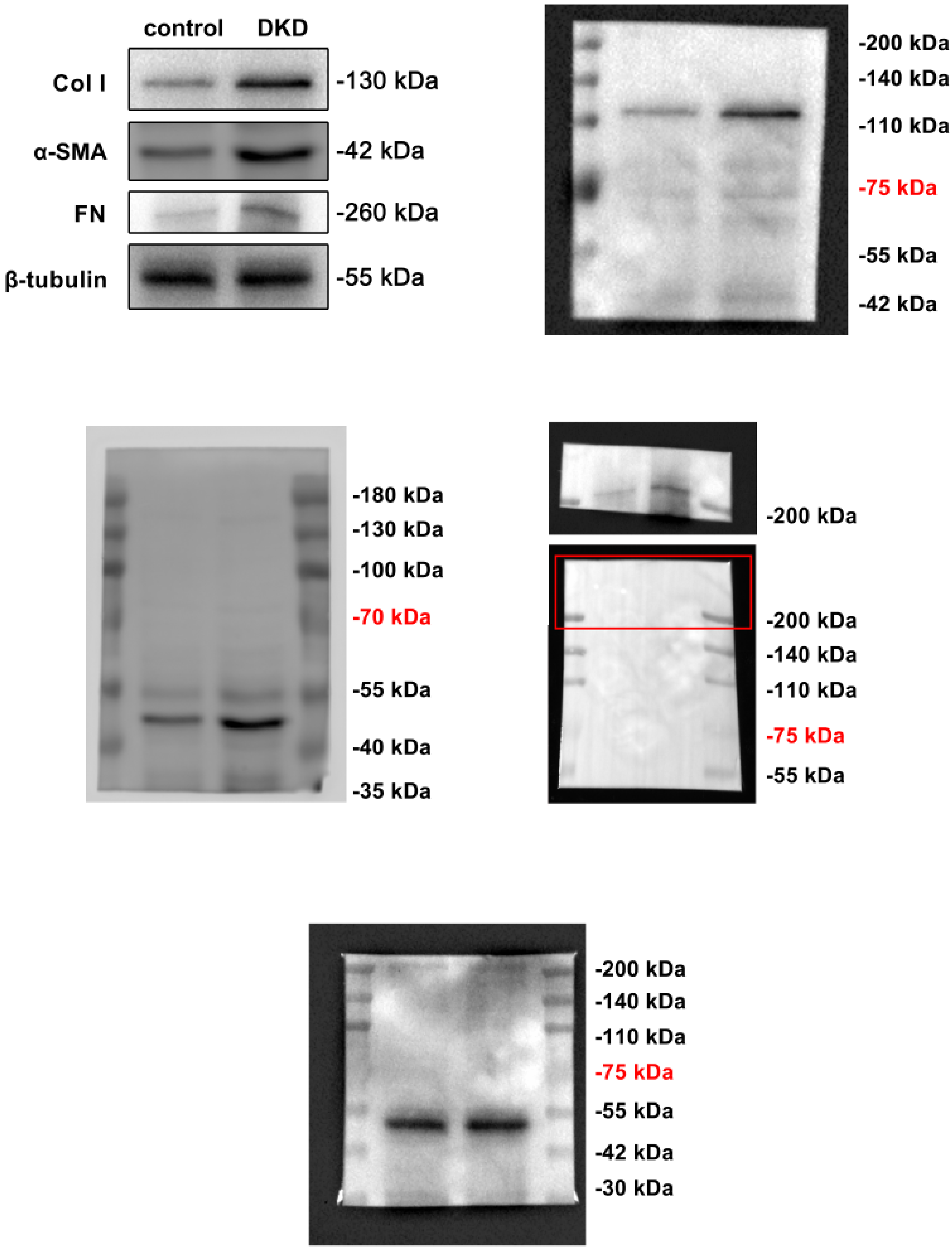
control
DKD
Col I
-130 kDa
α-SMA
-42 kDa
FN
-260 kDa
β-tubulin
-55 kDa
-200 kDa
-140 kDa
-110 kDa
-75 kDa
-55 kDa
-42 kDa
-180 kDa
-130 kDa
-100 kDa
-70 kDa
-55 kDa
-40 kDa
-35 kDa
-200 kDa
-200 kDa
-140 kDa
-110 kDa
-75 kDa
-55 kDa
-200 kDa
-140 kDa
-110 kDa
-75 kDa
-55 kDa
-42 kDa
-30 kDa

Figure. 1H

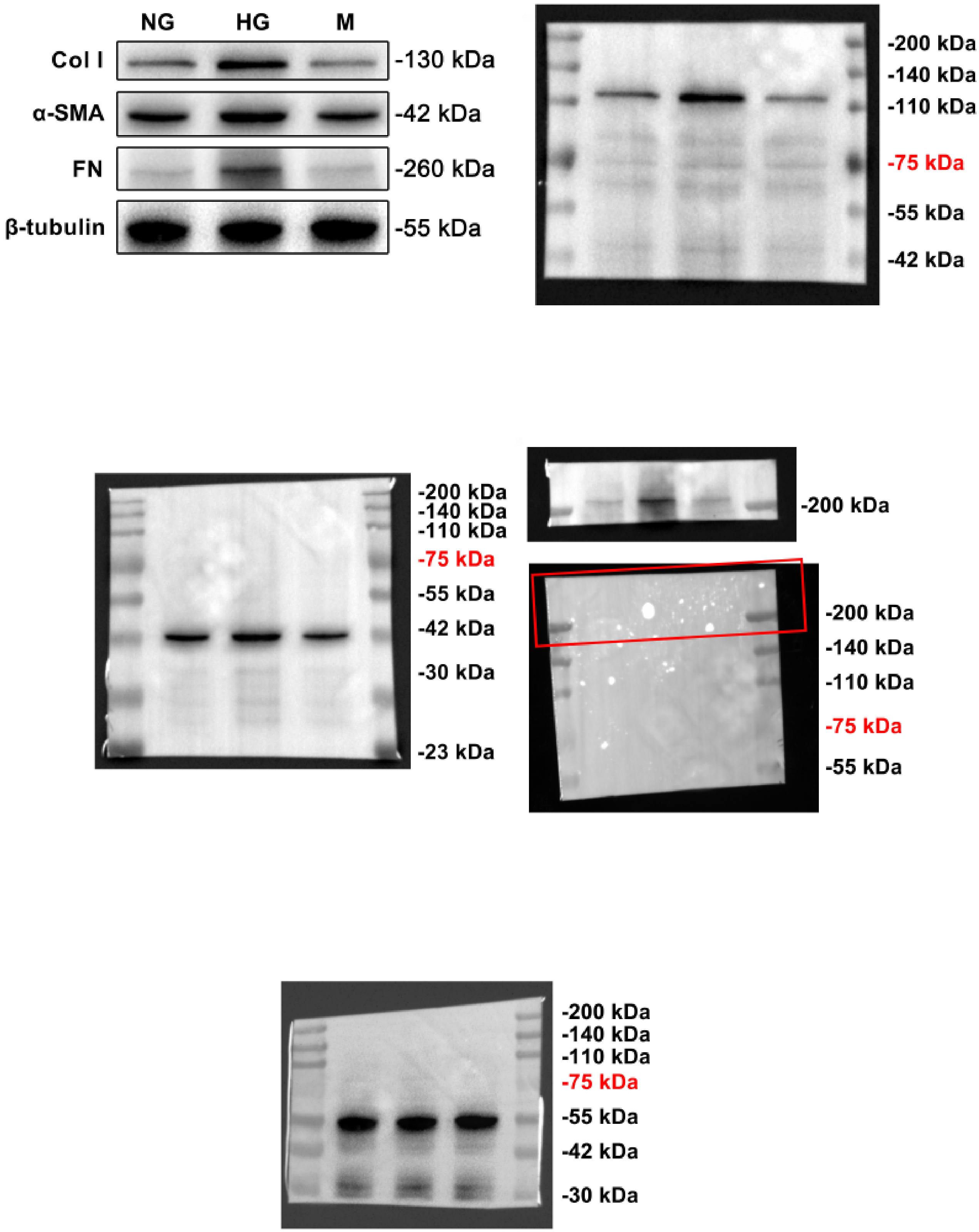

NG
HG
M
Col I
-130 kDa
α-SMA
-42 kDa
FN
-260 kDa
β-tubulin
-55 kDa
-200 kDa
-140 kDa
-110 kDa
-75 kDa
-55 kDa
-42 kDa
-200 kDa
-140 kDa
-110 kDa
-75 kDa
-55 kDa
-42 kDa
-30 kDa
-23 kDa
-200 kDa
-200 kDa
-140 kDa
-110 kDa
-75 kDa
-55 kDa
-200 kDa
-140 kDa
-110 kDa
-75 kDa
-55 kDa
-42 kDa
-30 kDa

Figure. 2D

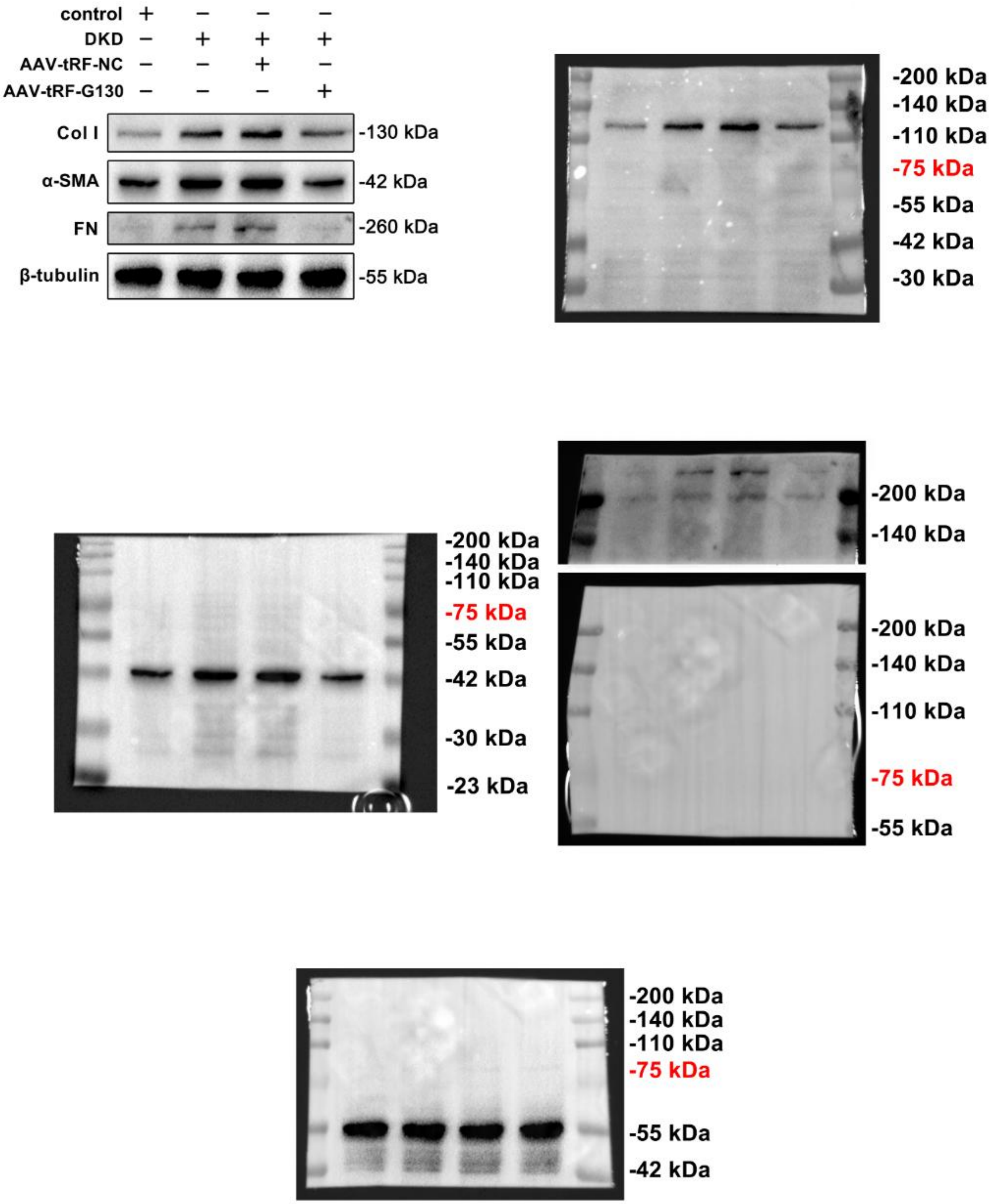

control + − − −
DKD − + + +
AAV-tRF-NC − − + −
AAV-tRF-G130 − − − +
Col I
-130 kDa
α-SMA
-42 kDa
FN
-260 kDa
β-tubulin
-55 kDa
-200 kDa
-140 kDa
-110 kDa
-75 kDa
-55 kDa
-42 kDa
-30 kDa
-200 kDa
-140 kDa
-110 kDa
-75 kDa
-55 kDa
-42 kDa
-30 kDa
-23 kDa
-200 kDa
-140 kDa
-200 kDa
-140 kDa
-110 kDa
-75 kDa
-55 kDa
-200 kDa
-140 kDa
-110 kDa
-75 kDa
-55 kDa
-42 kDa

Figure. 2G

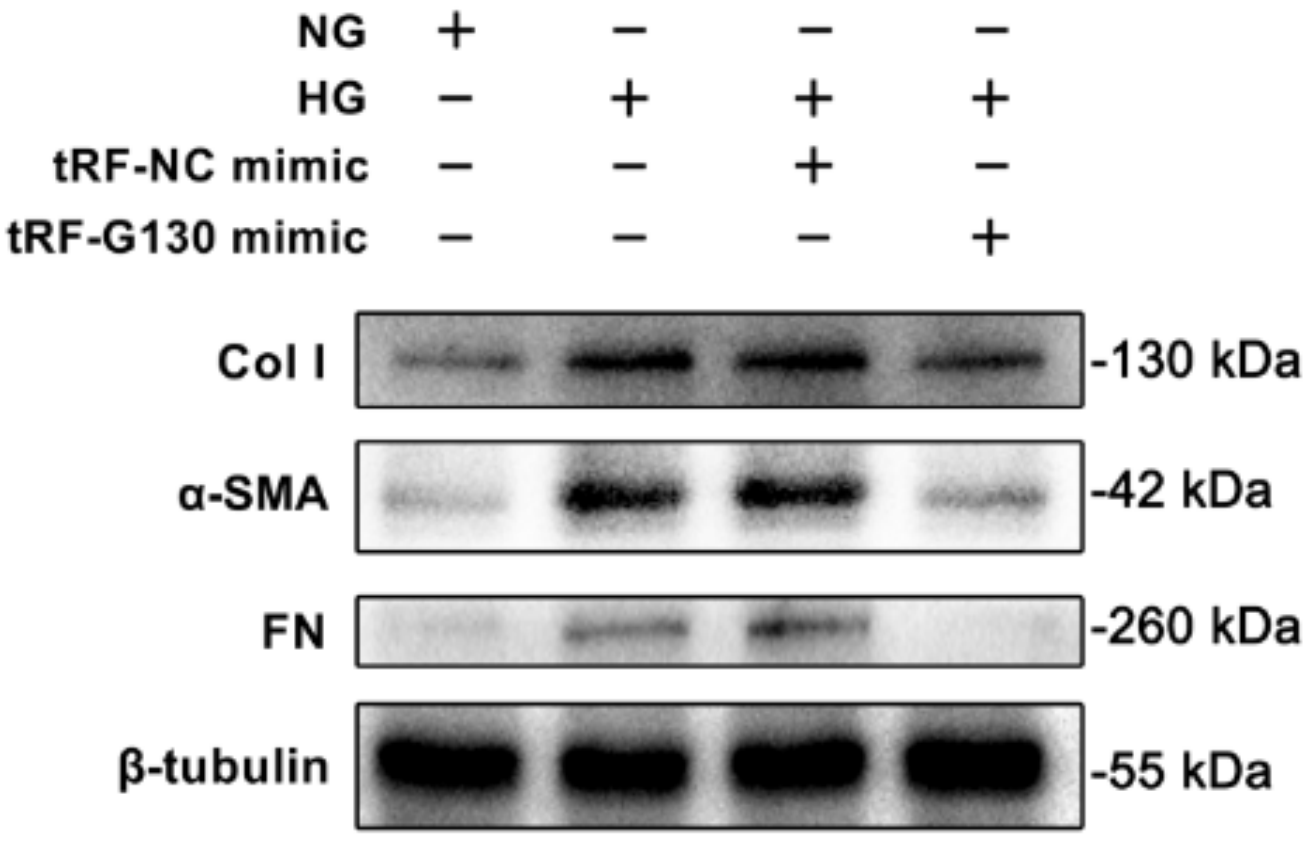
NG + − − −
HG − + + +
tRF-NC mimic − − + −
tRF-G130 mimic − − − +
Col I
α-SMA
FN
β-tubulin
-130 kDa
-42 kDa
-260 kDa
-55 kDa

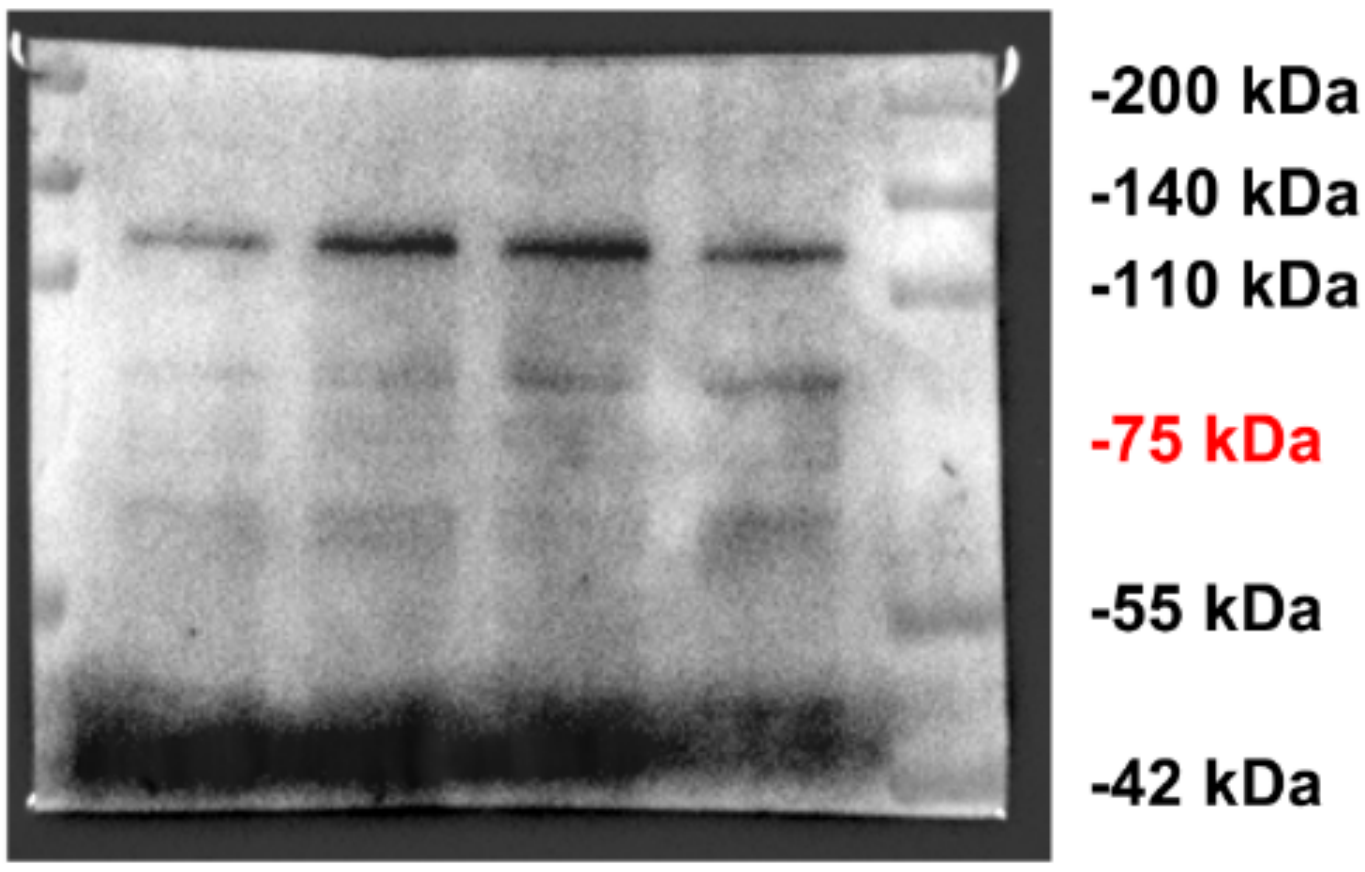
-200 kDa
-140 kDa
-110 kDa
-75 kDa
-55 kDa
-42 kDa

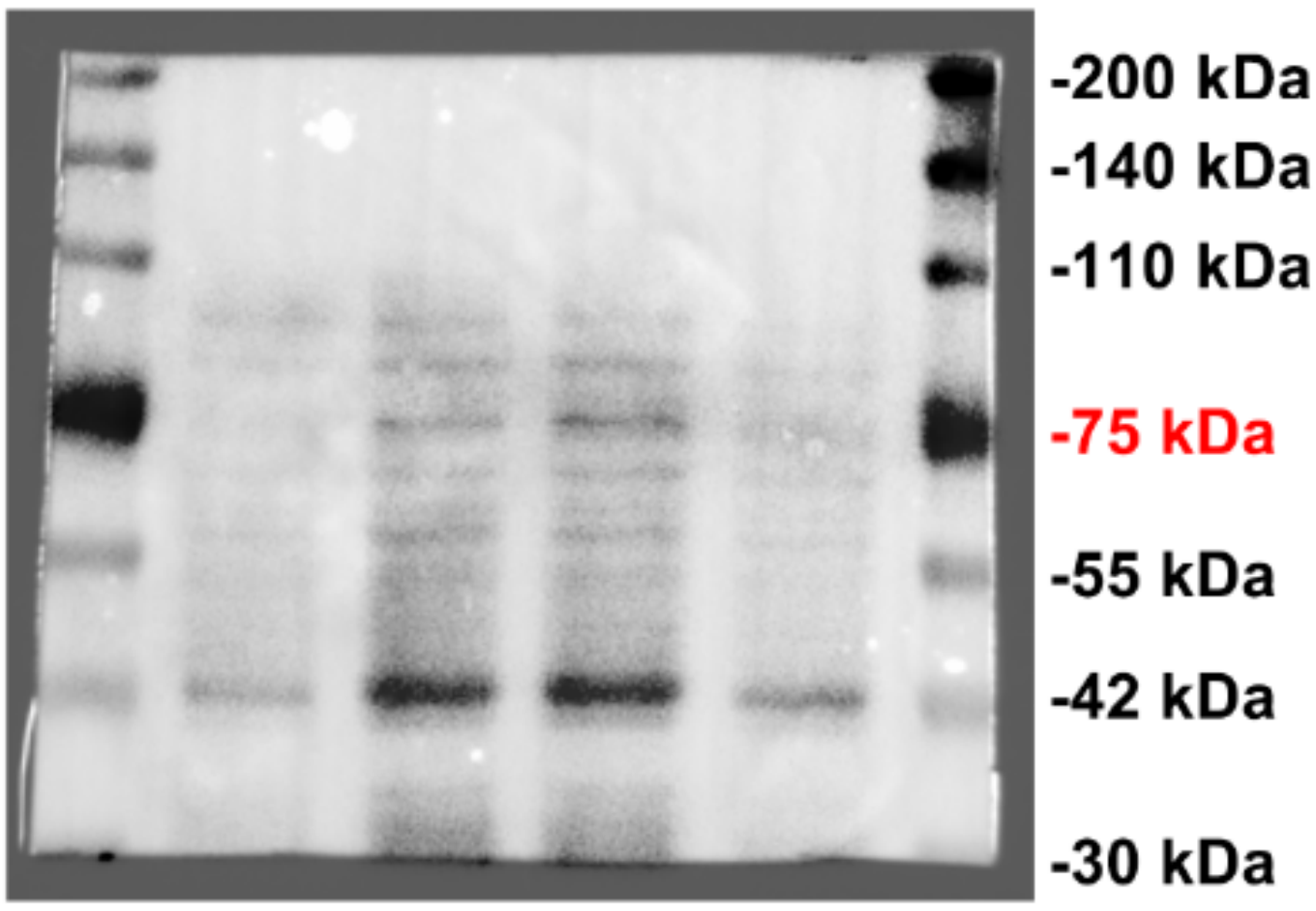
-200 kDa
-140 kDa
-110 kDa
-75 kDa
-55 kDa
-42 kDa
-30 kDa

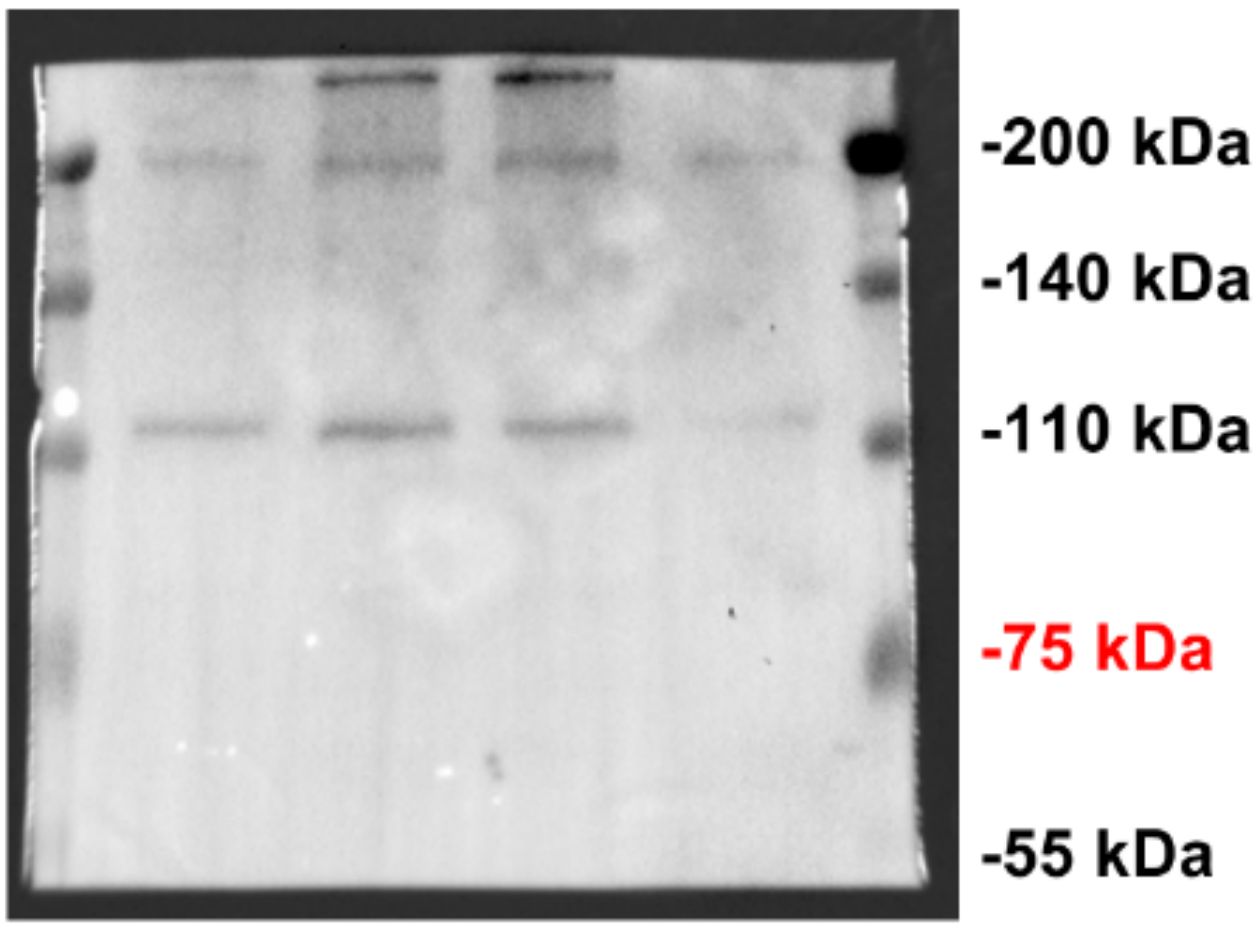
-200 kDa
-140 kDa
-110 kDa
-75 kDa
-55 kDa

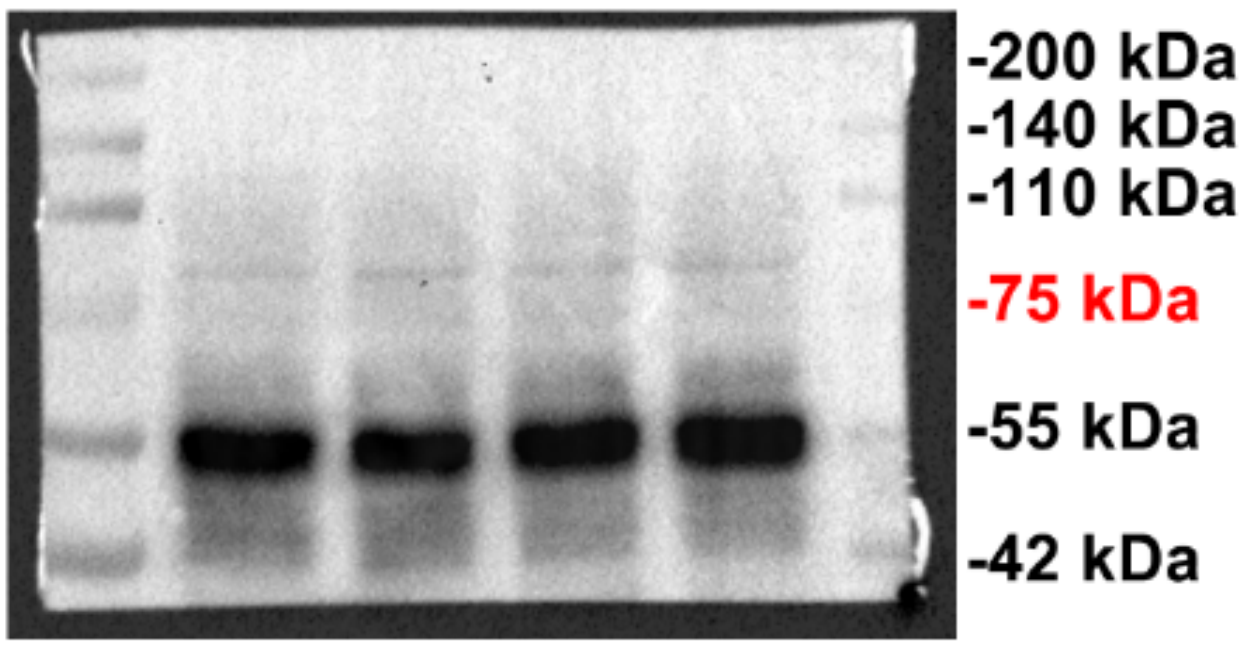
-200 kDa
-140 kDa
-110 kDa
-75 kDa
-55 kDa
-42 kDa

Figure. 3E

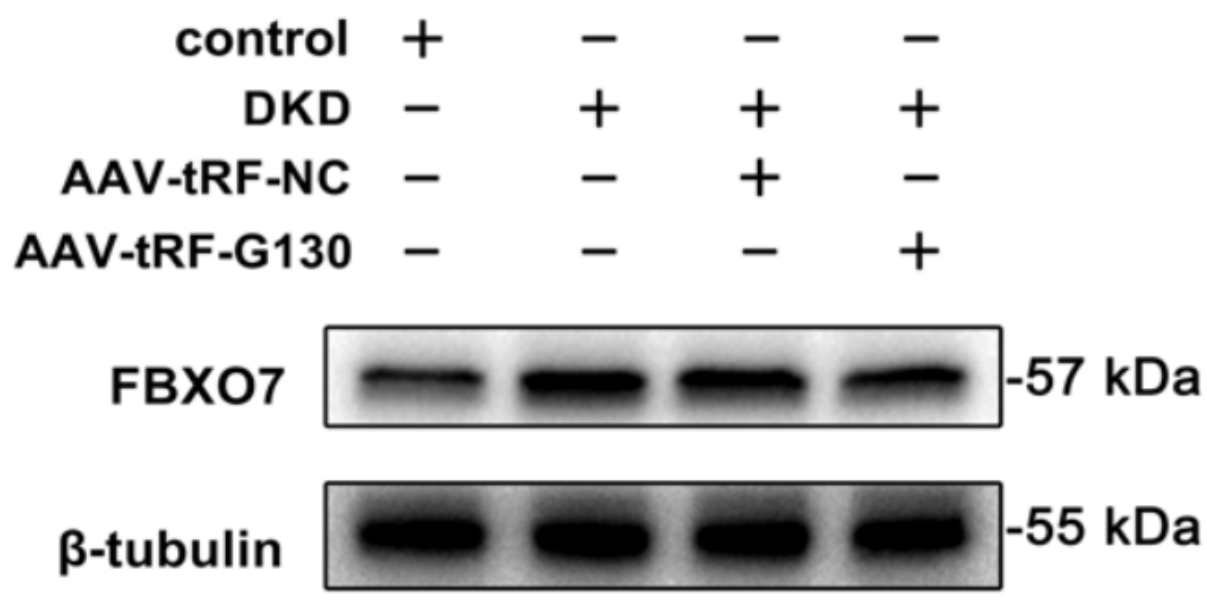
control + − − −
DKD − + + +
AAV-tRF-NC − − + −
AAV-tRF-G130 − − − +
FBXO7
-57 kDa
β-tubulin
-55 kDa

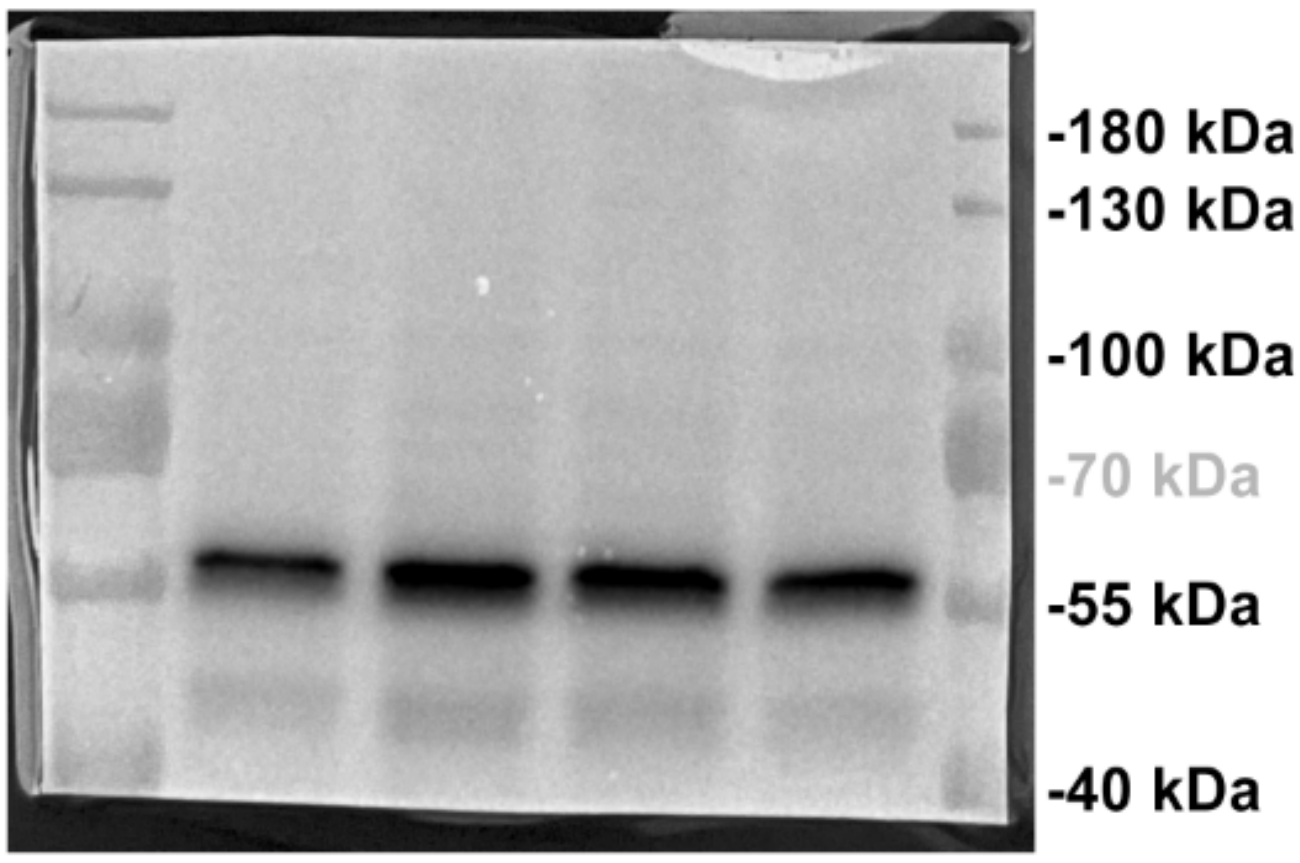
-180 kDa
-130 kDa
-100 kDa
-70 kDa
-55 kDa
-40 kDa

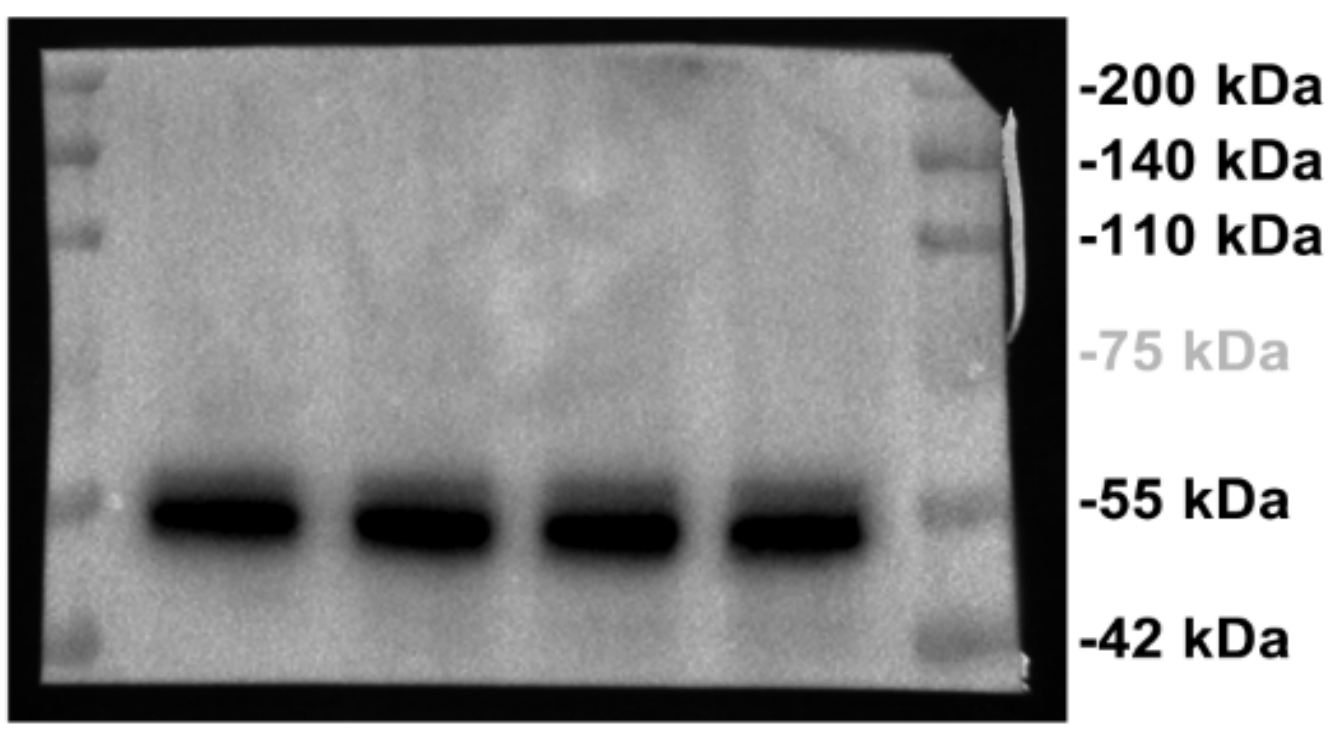
-200 kDa
-140 kDa
-110 kDa
-75 kDa
-55 kDa
-42 kDa

Figure. 3F

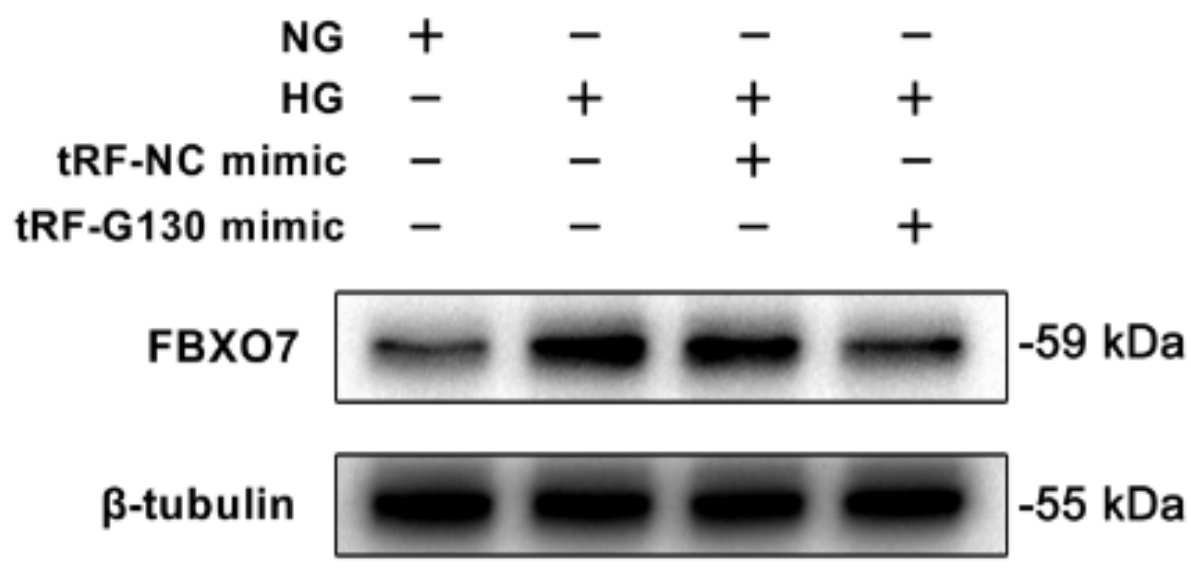
NG + − − −
HG − + + +
tRF-NC mimic − − + −
tRF-G130 mimic − − − +
FBXO7
-59 kDa
β-tubulin
-55 kDa

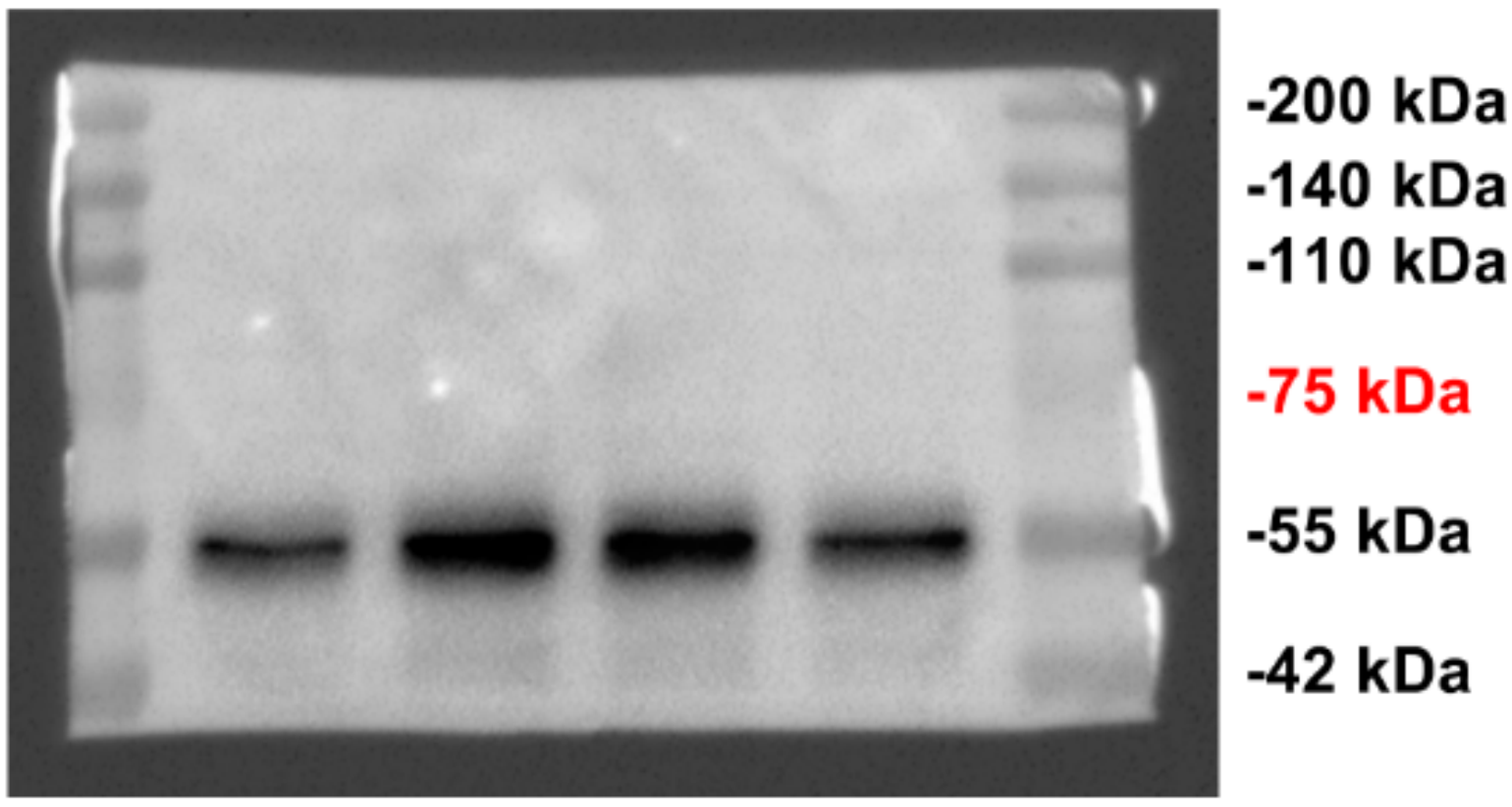
-200 kDa
-140 kDa
-110 kDa
-75 kDa
-55 kDa
-42 kDa

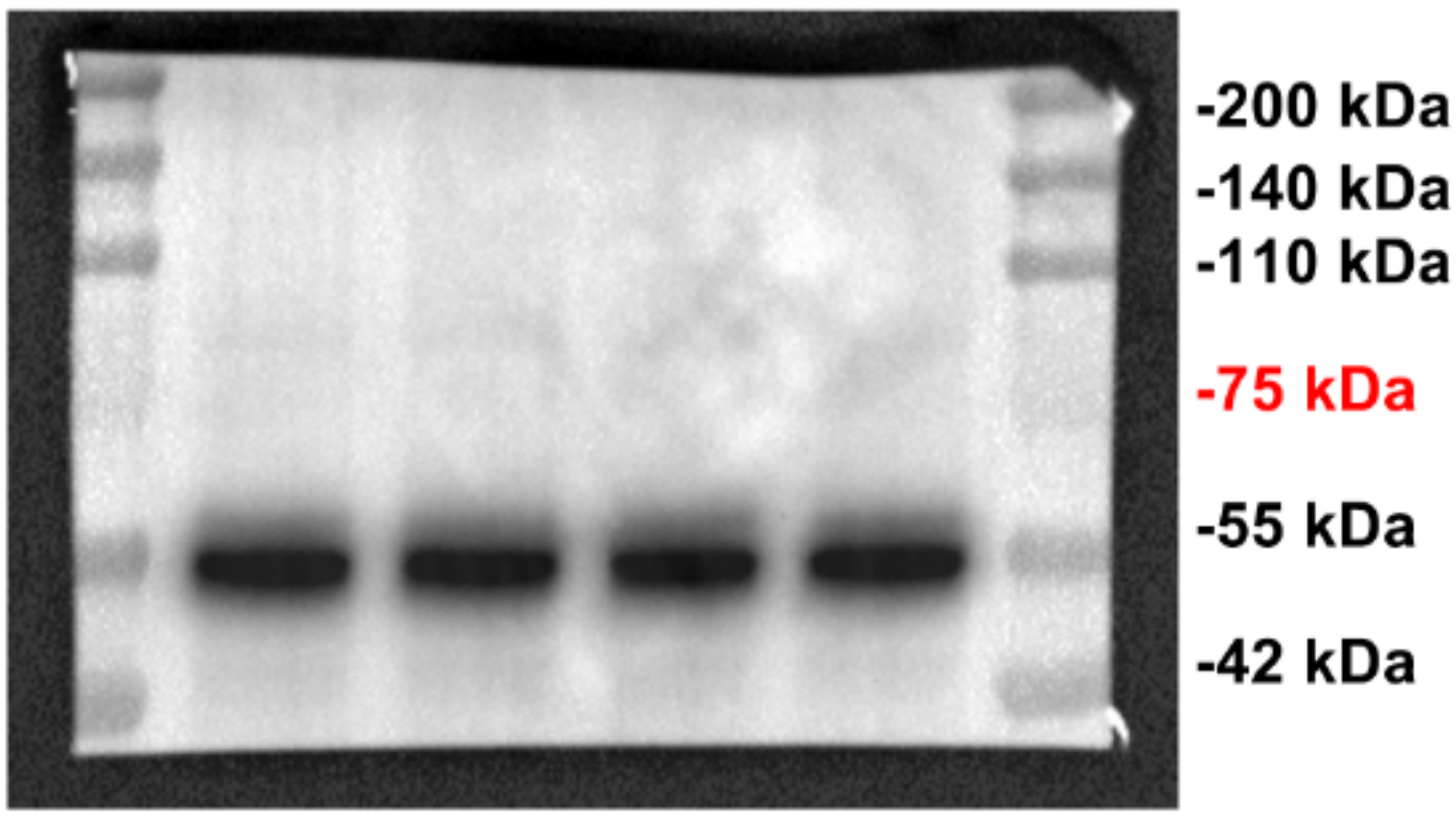
-200 kDa
-140 kDa
-110 kDa
-75 kDa
-55 kDa
-42 kDa

Figure. 3G

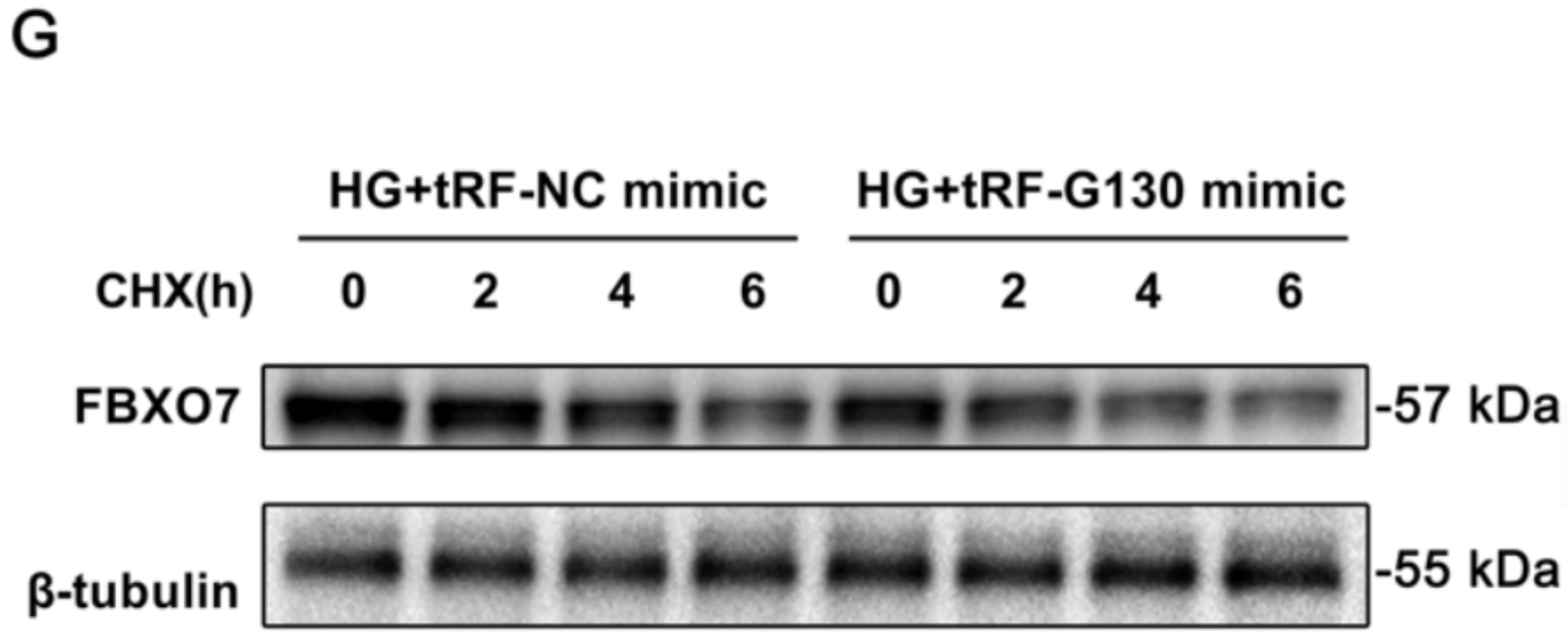

G
HG+tRF-NC mimic
HG+tRF-G130 mimic
CHX(h)
0
2
4
6
0
2
4
6
FBXO7
-57 kDa
β-tubulin
-55 kDa

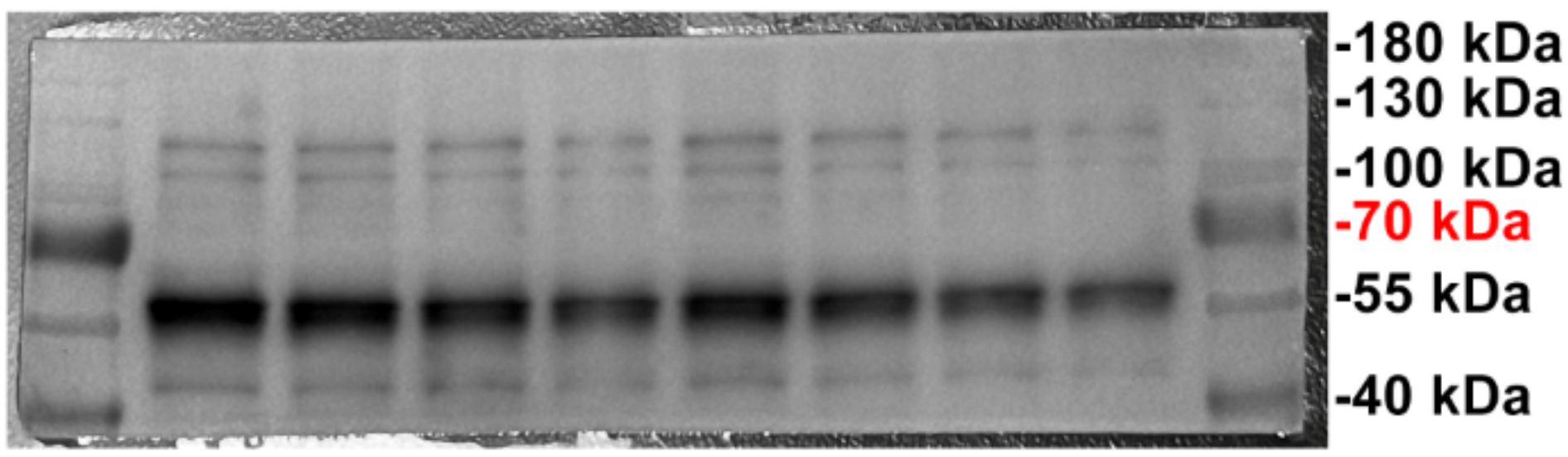

-180 kDa
-130 kDa
-100 kDa
-70 kDa
-55 kDa
-40 kDa

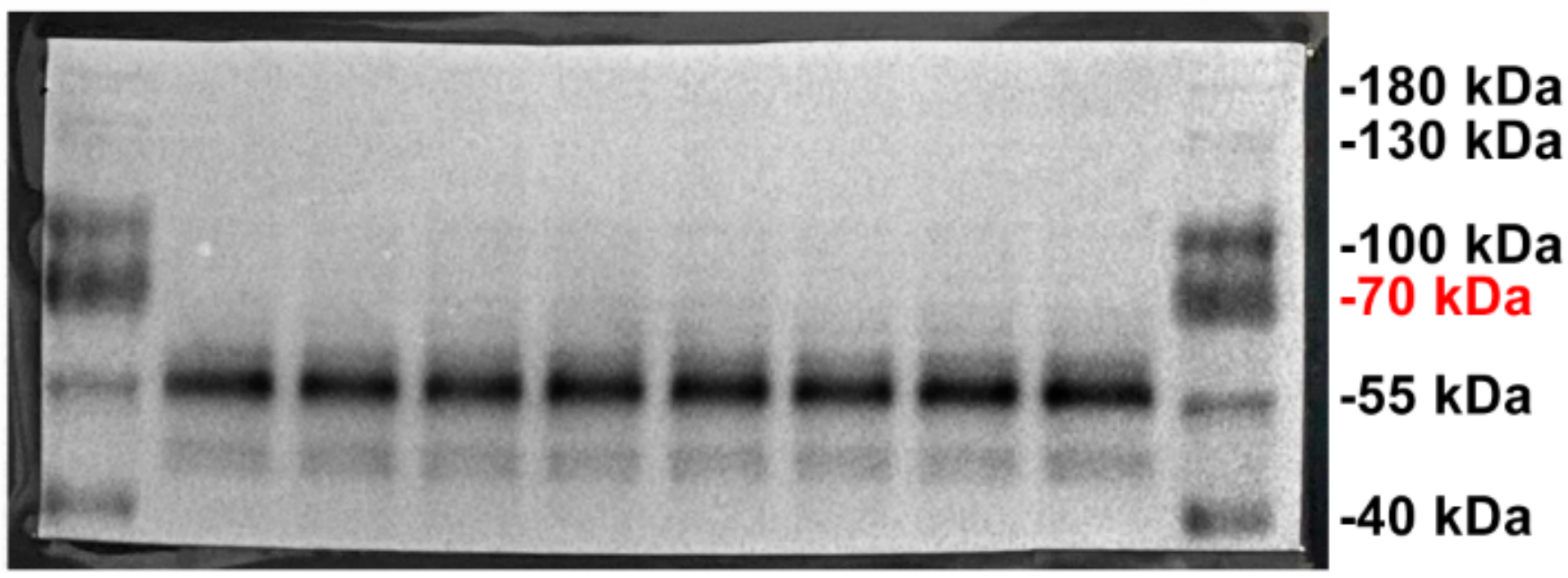

-180 kDa
-130 kDa
-100 kDa
-70 kDa
-55 kDa
-40 kDa

Figure. 3H

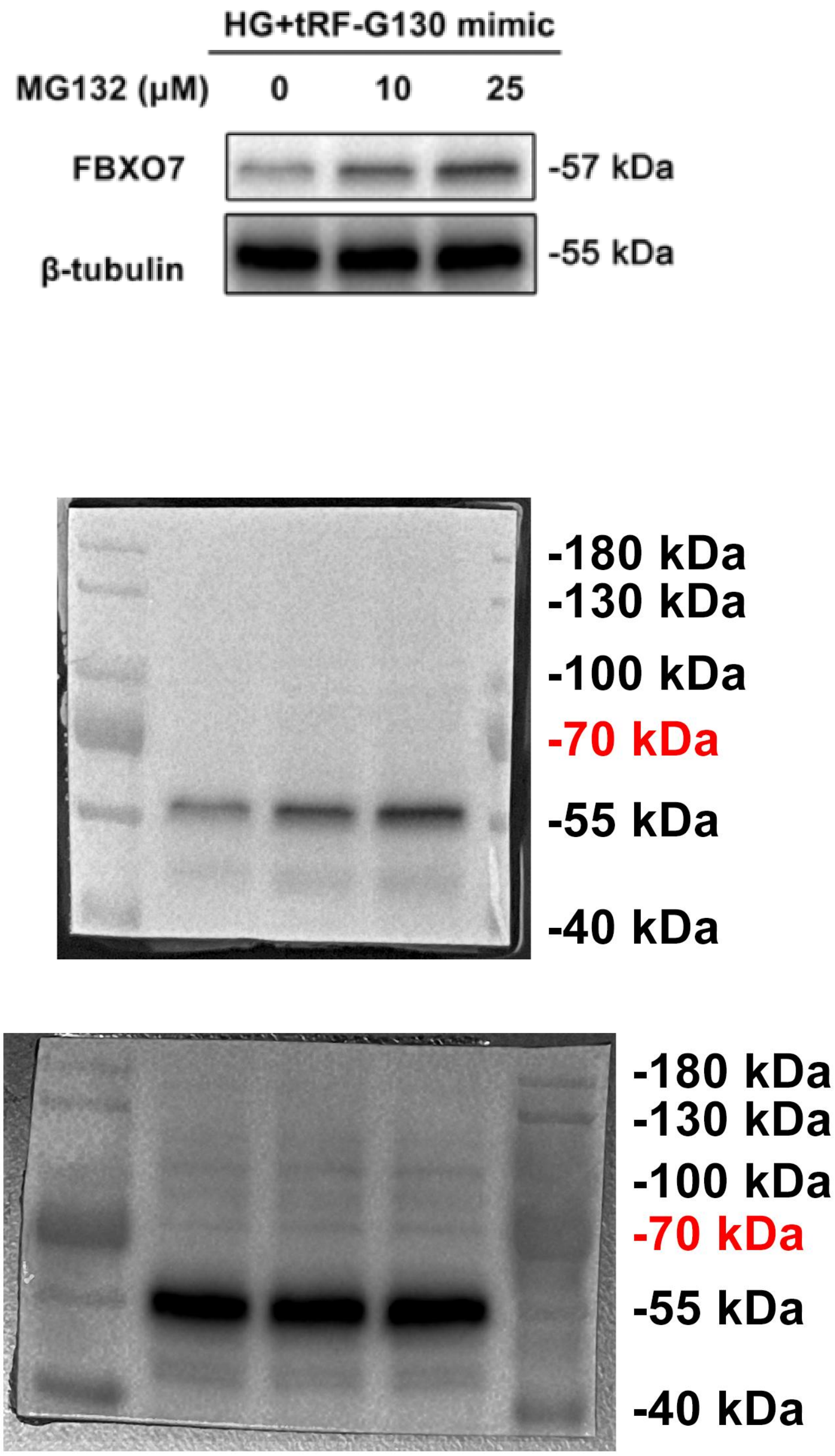
HG+tRF-G130 mimic
MG132 (μM)
0
10
25
FBXO7
-57 kDa
β-tubulin
-55 kDa
-180 kDa
-130 kDa
-100 kDa
-70 kDa
-55 kDa
-40 kDa
-180 kDa
-130 kDa
-100 kDa
-70 kDa
-55 kDa
-40 kDa

Figure. 3I

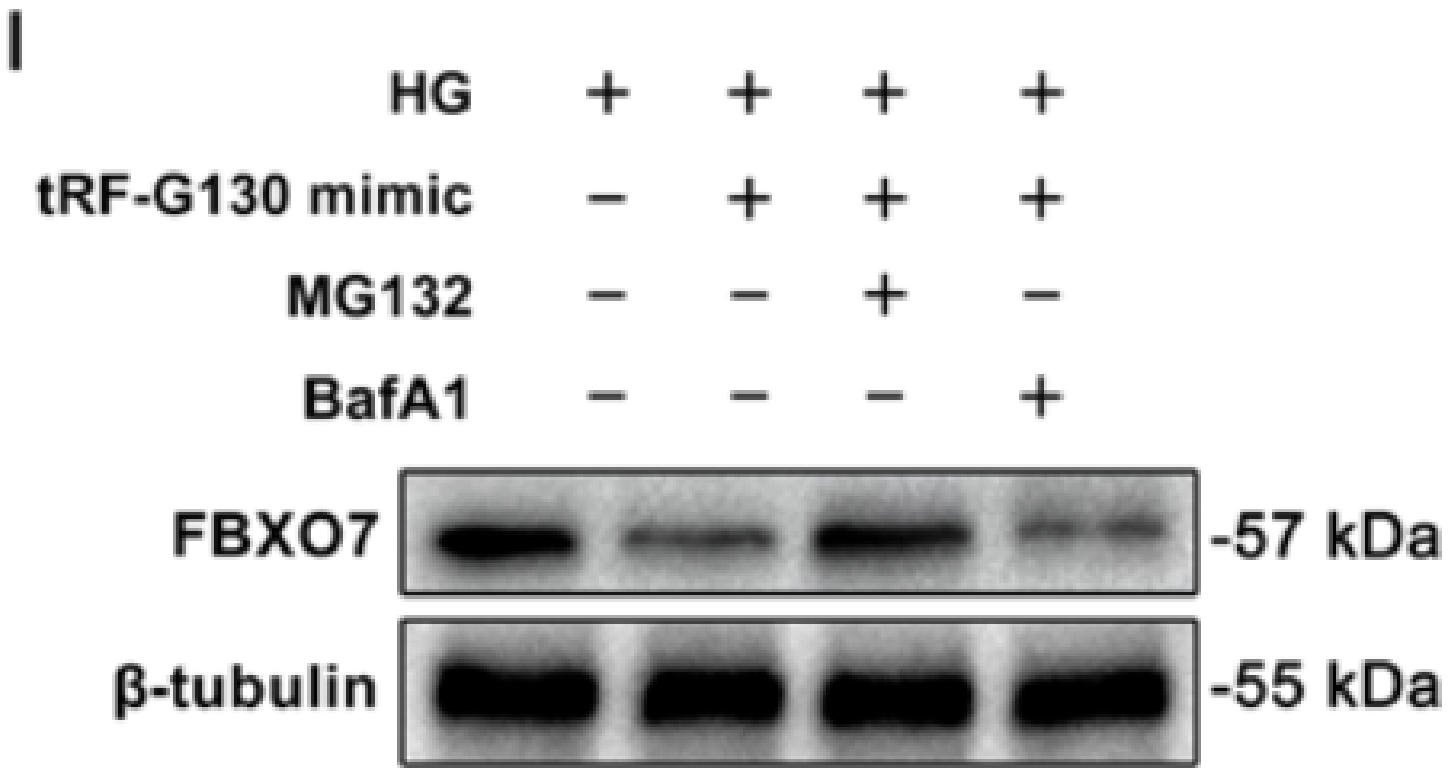
I
HG + + + +
tRF-G130 mimic − + + +
MG132 − − + −
BafA1 − − − +
FBXO7
-57 kDa
β-tubulin
-55 kDa

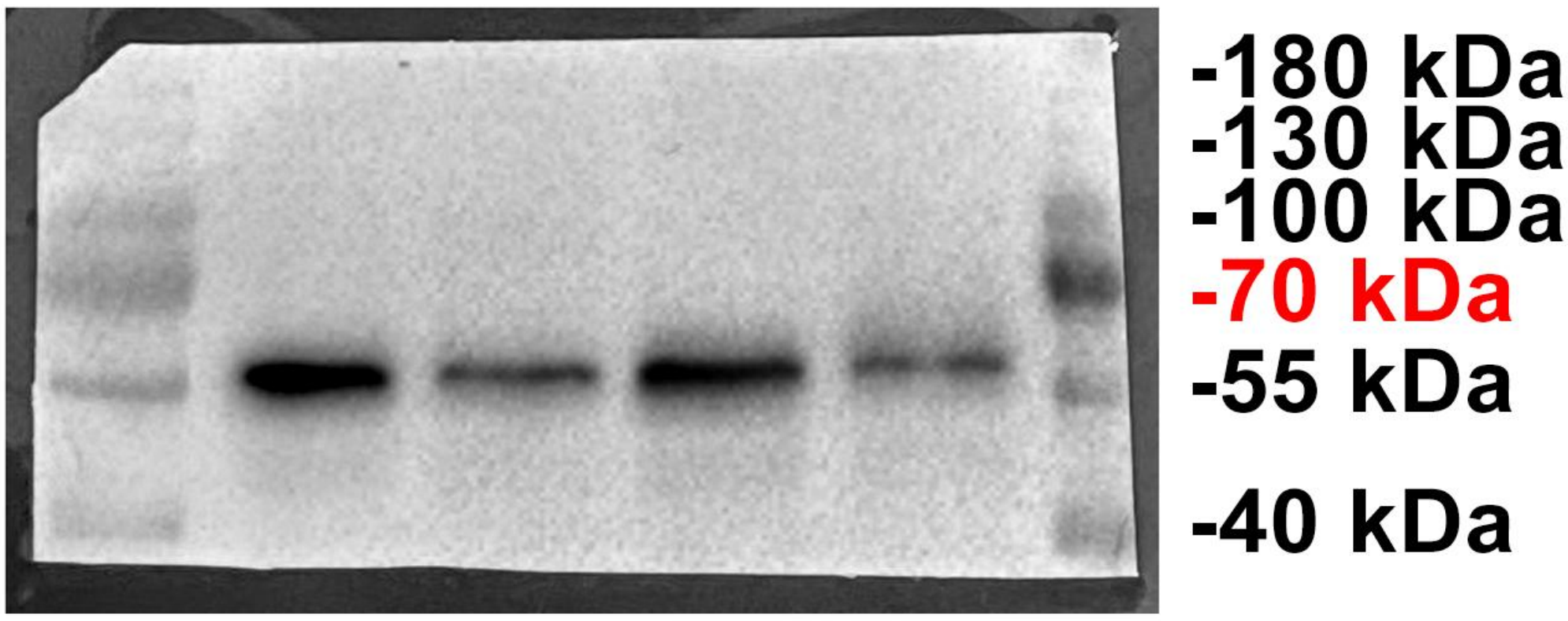
-180 kDa
-130 kDa
-100 kDa
-70 kDa
-55 kDa
-40 kDa

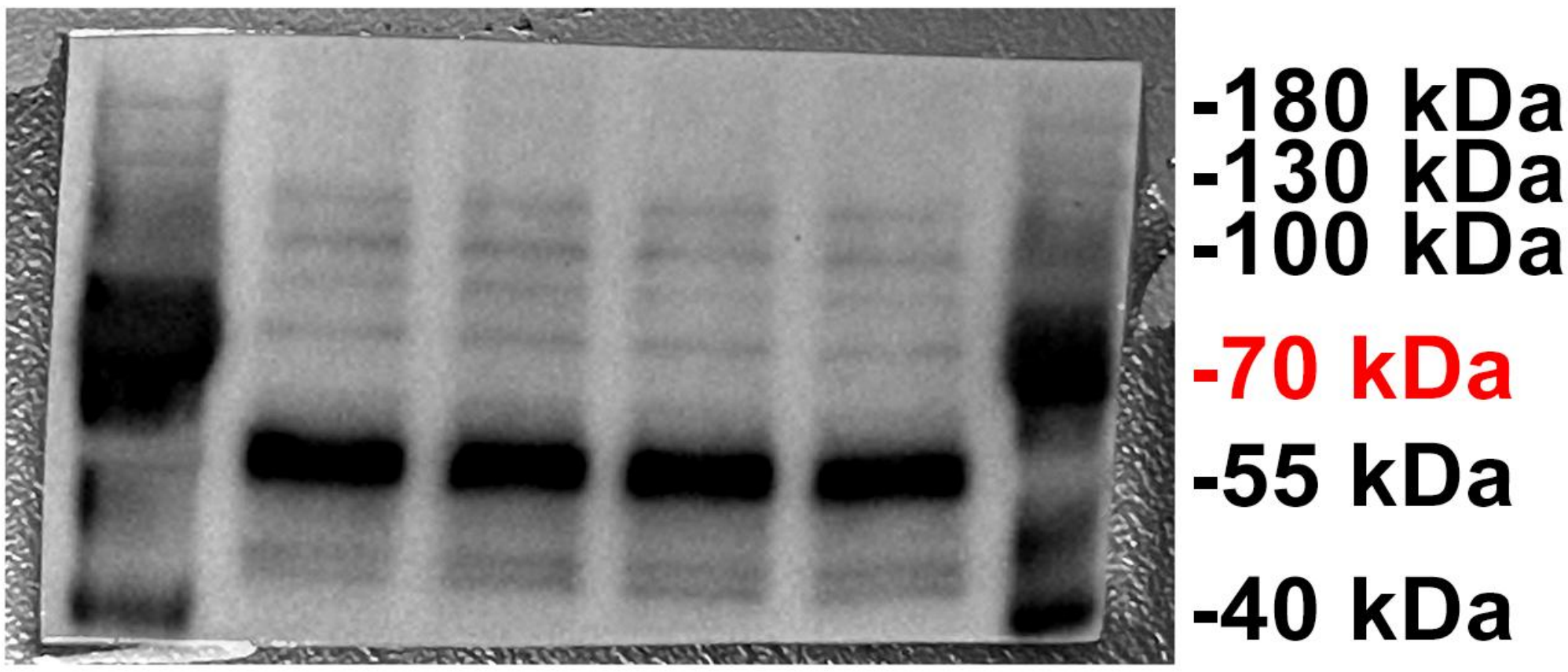
-180 kDa
-130 kDa
-100 kDa
-70 kDa
-55 kDa
-40 kDa

Figure. 3J

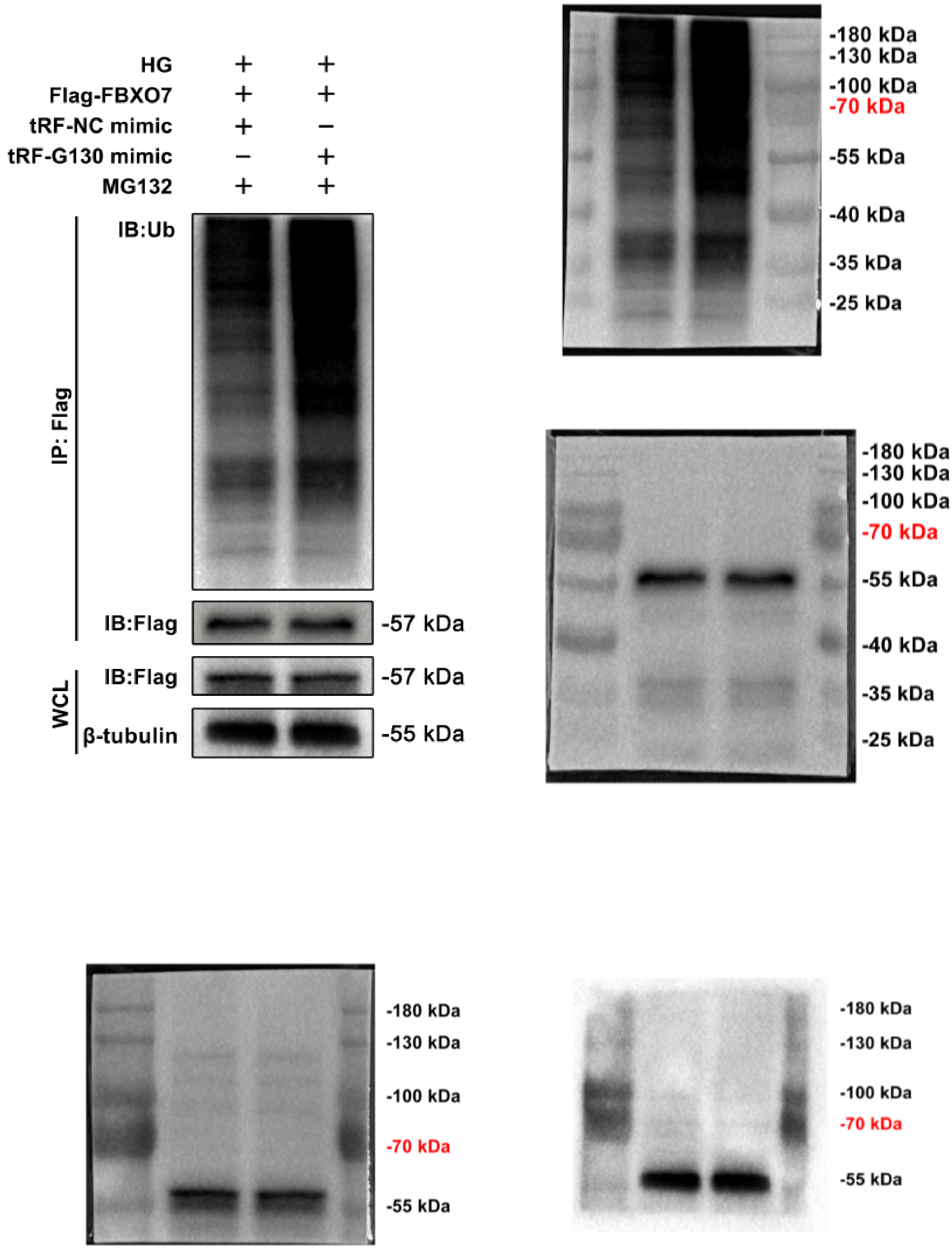
HG + +
Flag-FBXO7 + +
tRF-NC mimic + −
tRF-G130 mimic − +
MG132 + +
IP: Flag
IB:Ub
IB:Flag
-57 kDa
WCL
IB:Flag
-57 kDa
β-tubulin
-55 kDa
-180 kDa
-130 kDa
-100 kDa
-70 kDa
-55 kDa
-40 kDa
-35 kDa
-25 kDa
-180 kDa
-130 kDa
-100 kDa
-70 kDa
-55 kDa
-40 kDa
-35 kDa
-25 kDa
-180 kDa
-130 kDa
-100 kDa
-70 kDa
-55 kDa
-180 kDa
-130 kDa
-100 kDa
-70 kDa
-55 kDa

Figure. 4B

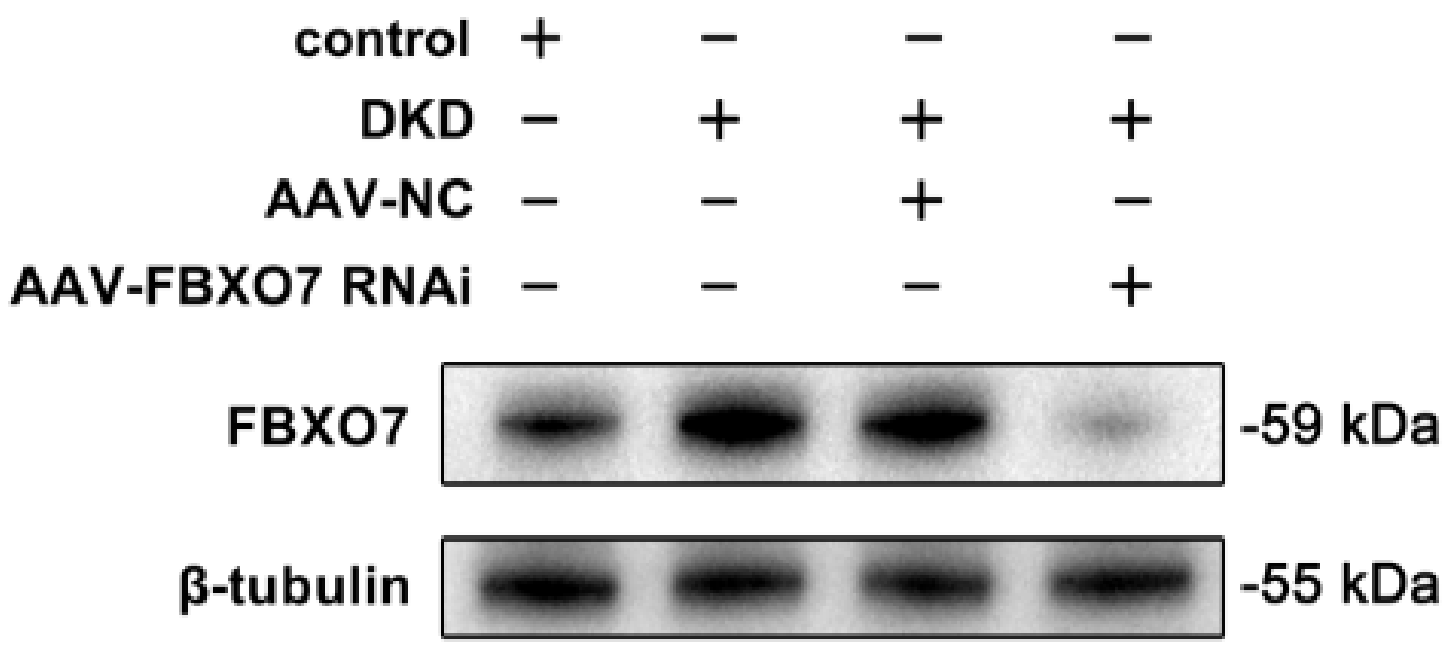

control + − − −
DKD − + + +
AAV-NC − − + −
AAV-FBXO7 RNAi − − − +
FBXO7
-59 kDa
β-tubulin
-55 kDa

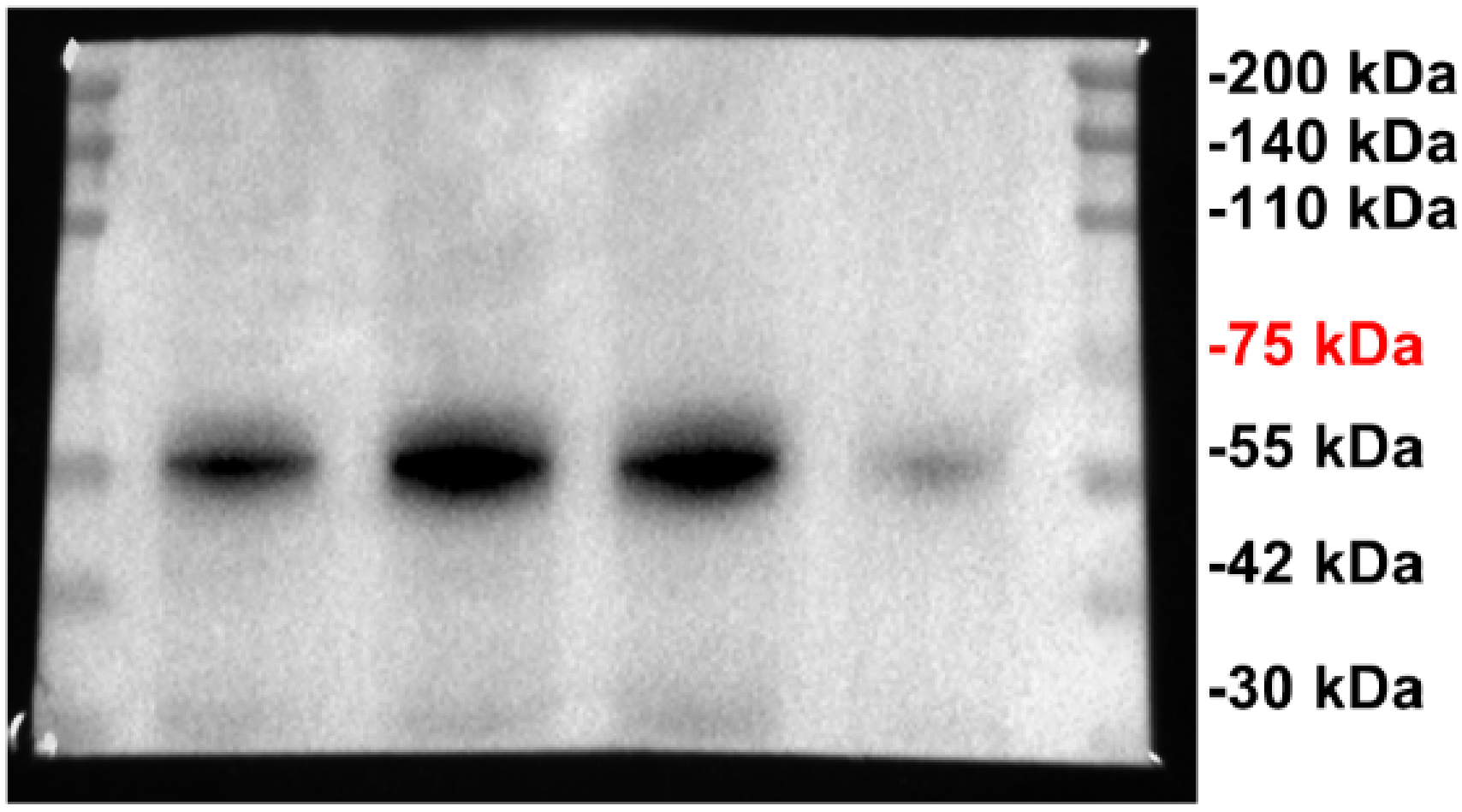

-200 kDa
-140 kDa
-110 kDa
-75 kDa
-55 kDa
-42 kDa
-30 kDa

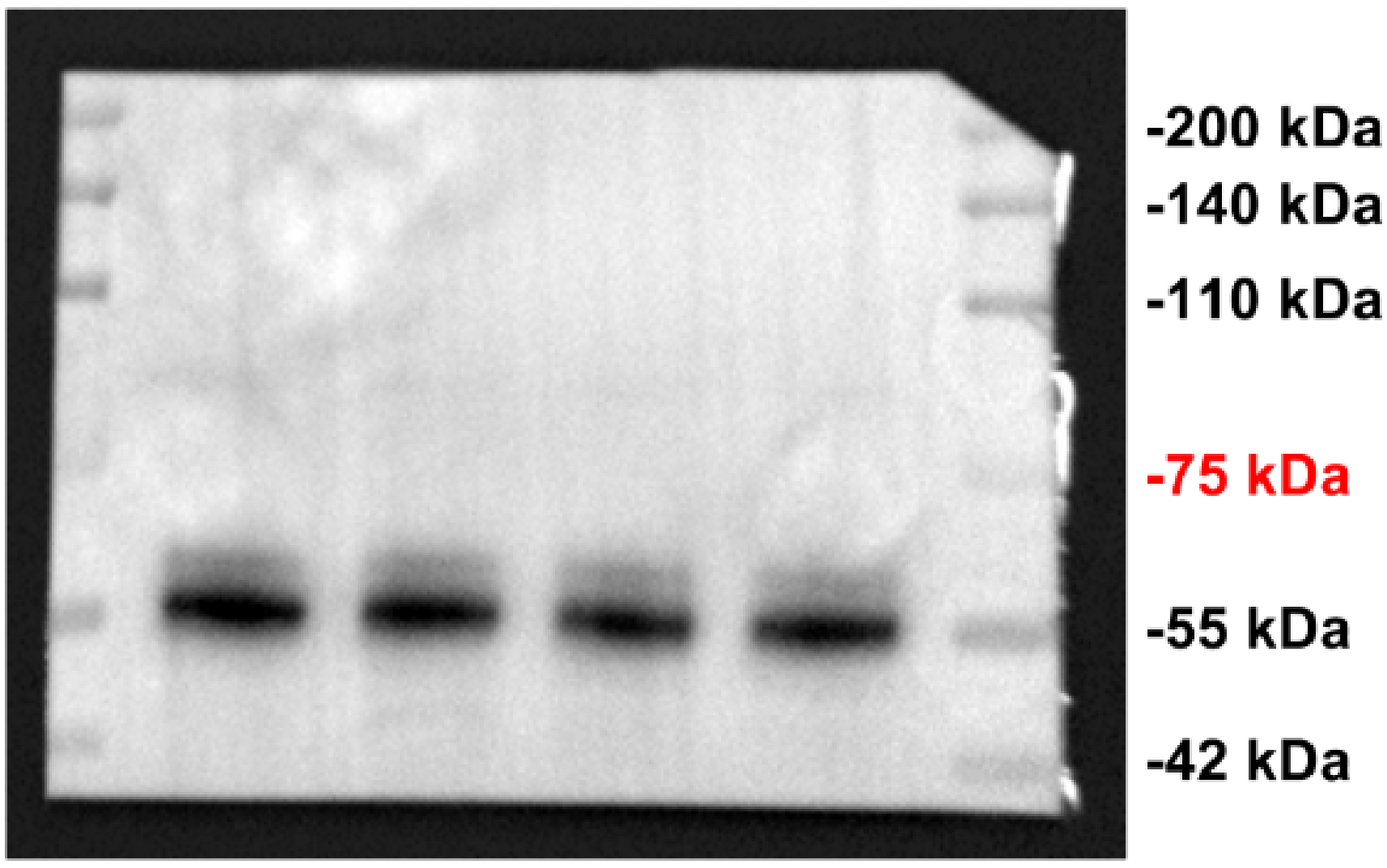

-200 kDa
-140 kDa
-110 kDa
-75 kDa
-55 kDa
-42 kDa

Figure. 4G

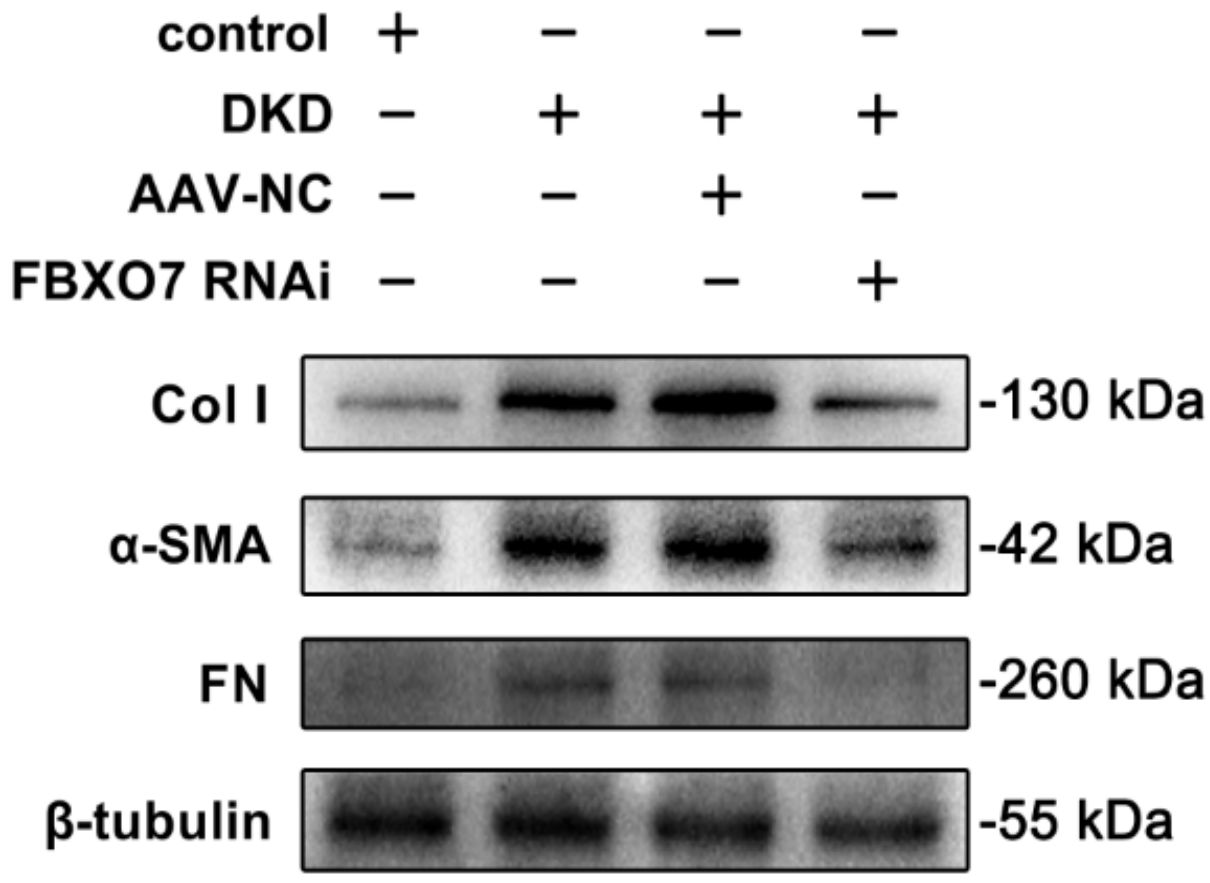
control + − − −
DKD − + + +
AAV-NC − − + −
FBXO7 RNAi − − − +
Col I
α-SMA
FN
β-tubulin
-130 kDa
-42 kDa
-260 kDa
-55 kDa

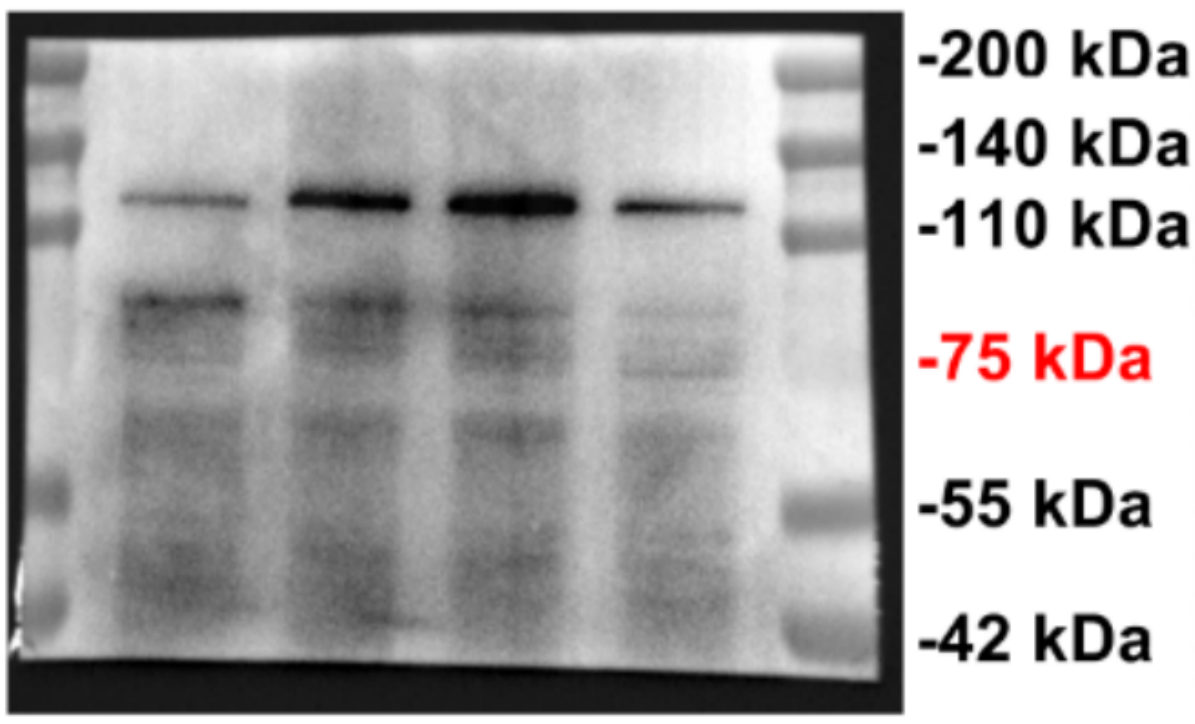
-200 kDa
-140 kDa
-110 kDa
-75 kDa
-55 kDa
-42 kDa

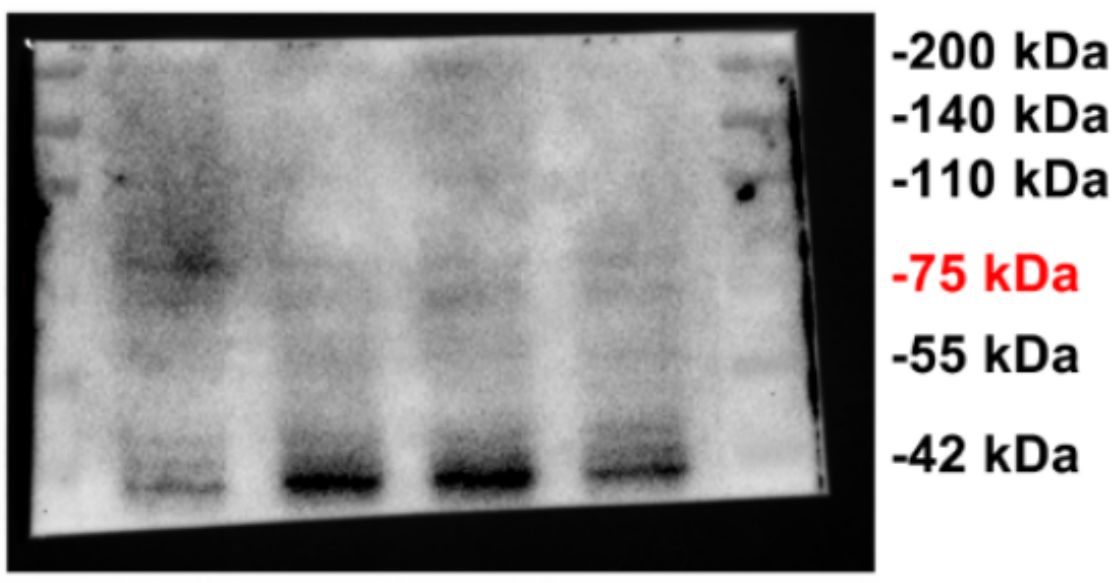
-200 kDa
-140 kDa
-110 kDa
-75 kDa
-55 kDa
-42 kDa

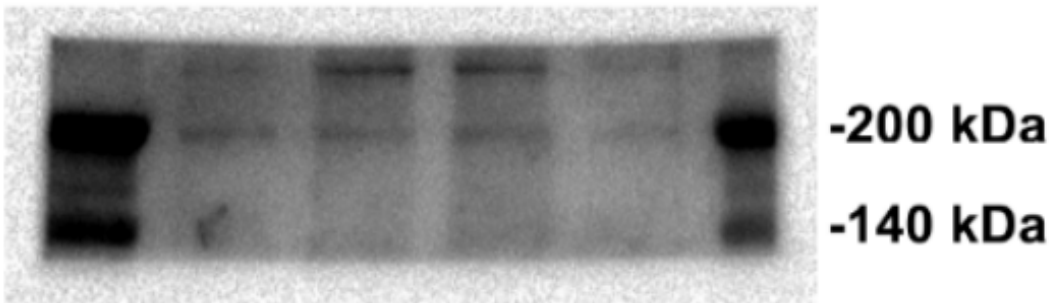
-200 kDa
-140 kDa

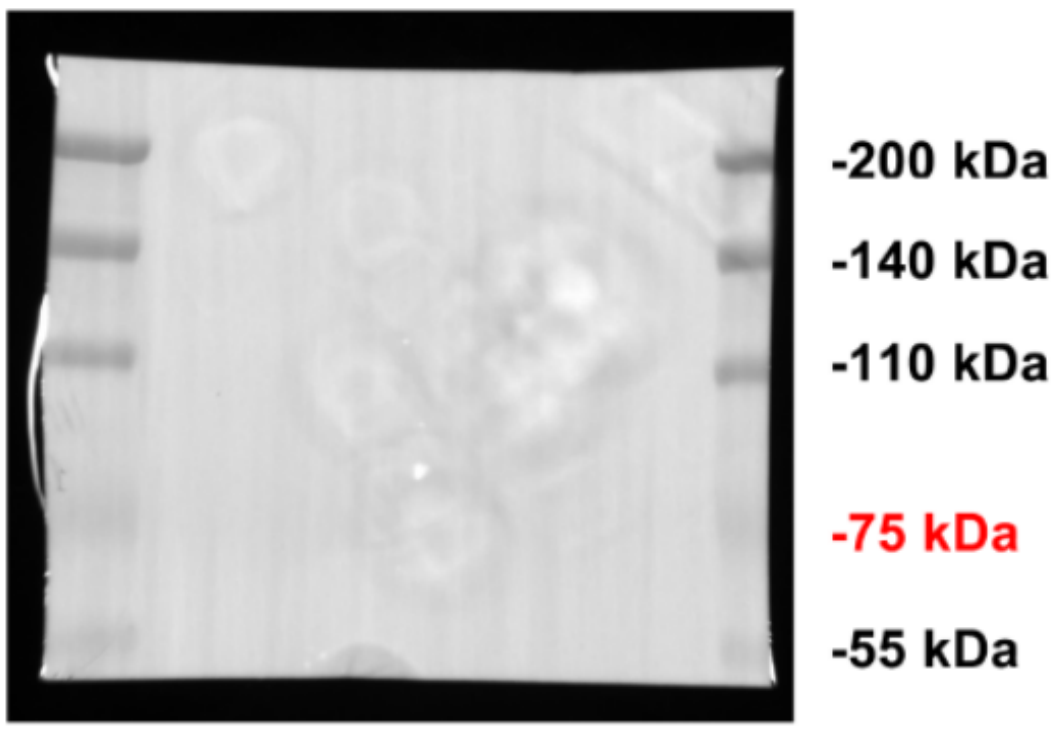
-200 kDa
-140 kDa
-110 kDa
-75 kDa
-55 kDa

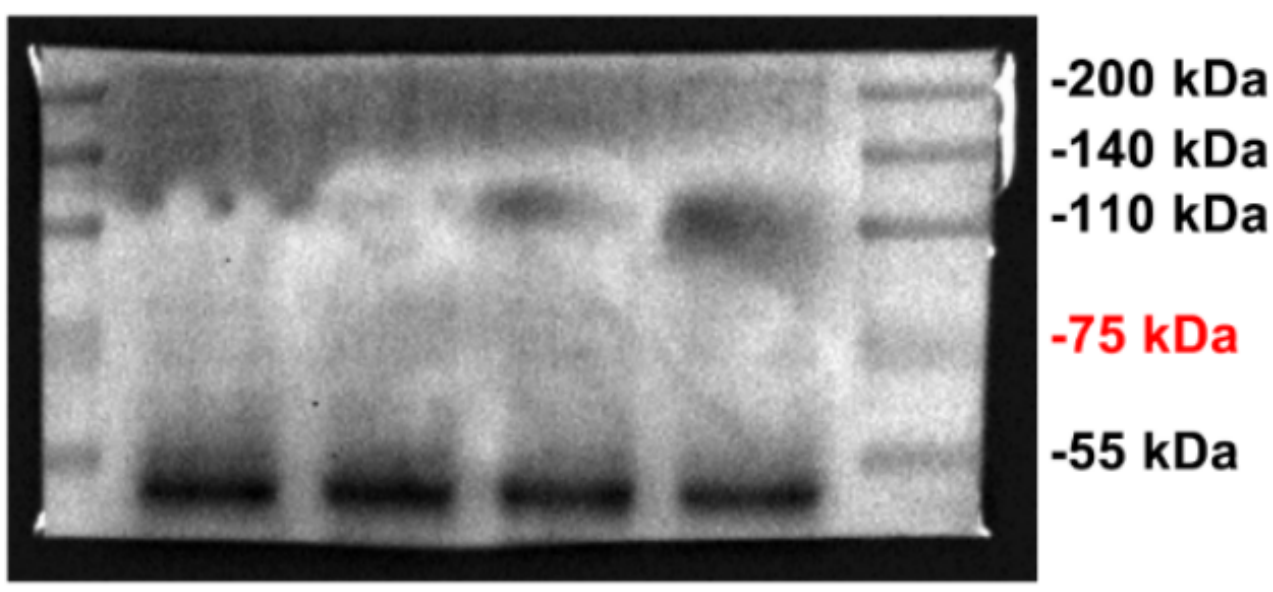
-200 kDa
-140 kDa
-110 kDa
-75 kDa
-55 kDa

Figure. S2E

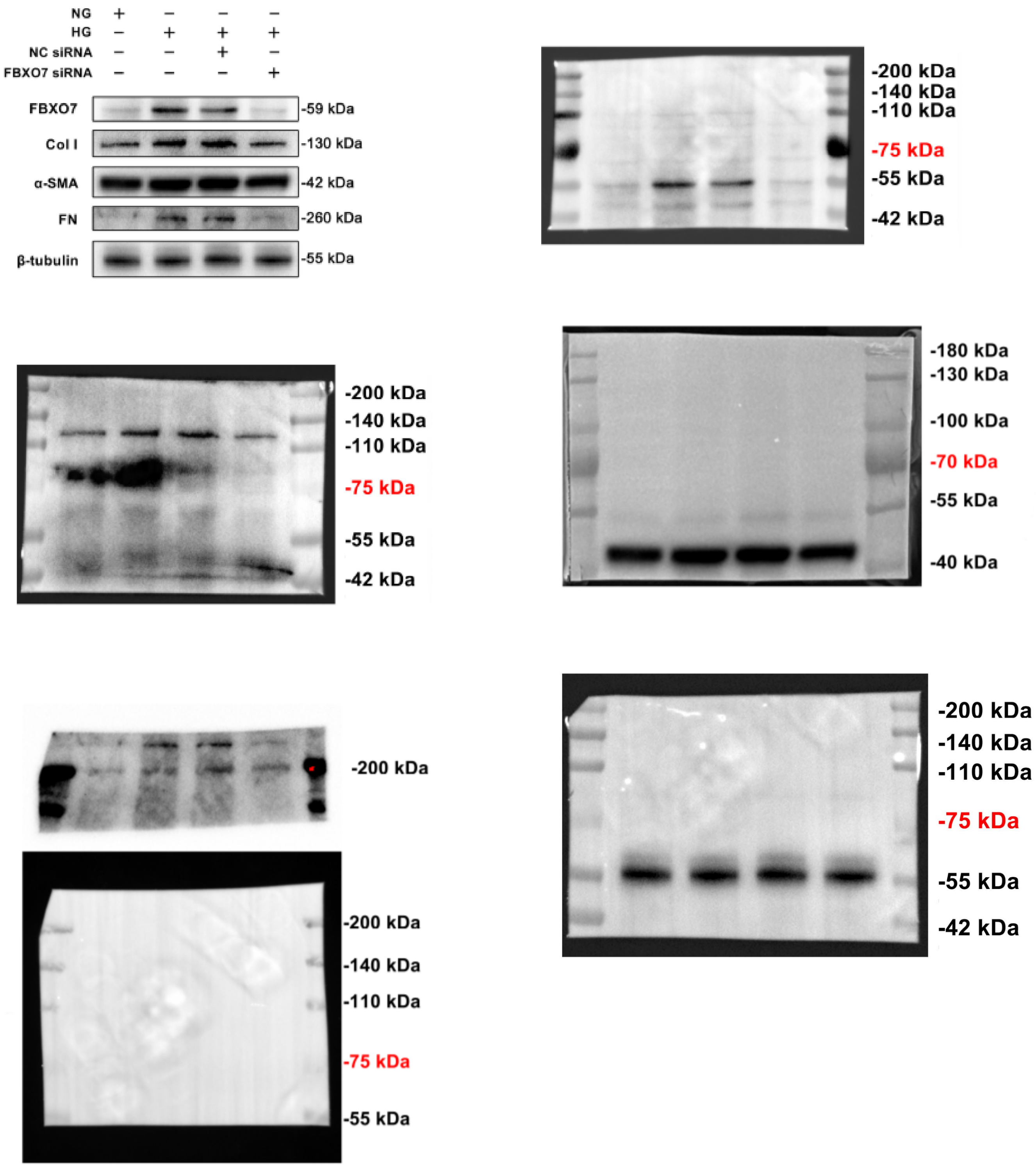

NG + − − −
HG − + + +
NC siRNA − − + −
FBXO7 siRNA − − − +
FBXO7
-59 kDa
Col I
-130 kDa
α-SMA
-42 kDa
FN
-260 kDa
β-tubulin
-55 kDa
-200 kDa
-140 kDa
-110 kDa
-75 kDa
-55 kDa
-42 kDa
-200 kDa
-140 kDa
-110 kDa
-75 kDa
-55 kDa
-42 kDa
-180 kDa
-130 kDa
-100 kDa
-70 kDa
-55 kDa
-40 kDa
-200 kDa
-200 kDa
-140 kDa
-110 kDa
-75 kDa
-55 kDa
-200 kDa
-140 kDa
-110 kDa
-75 kDa
-55 kDa
-42 kDa

Figure. 4H

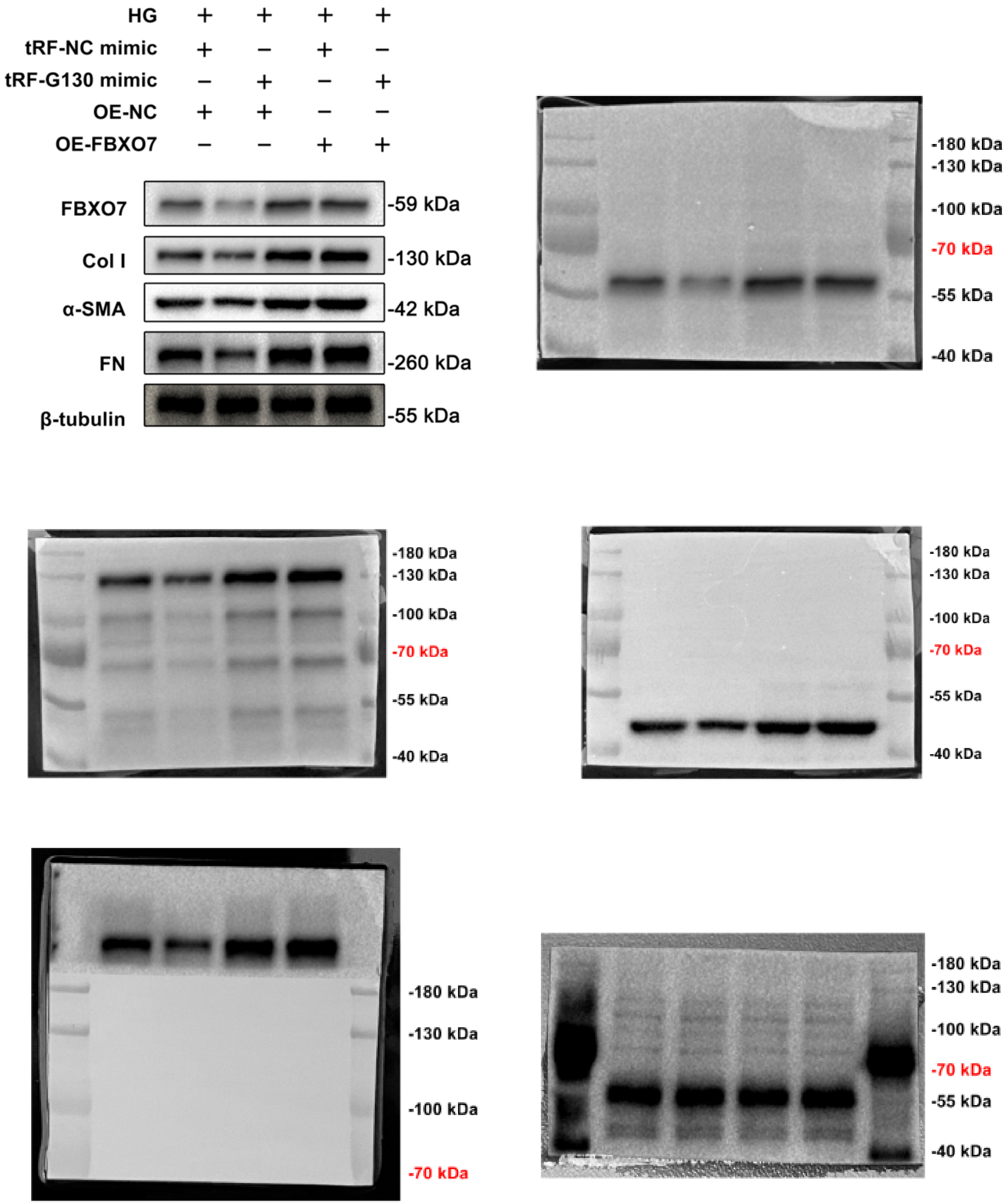

HG + + + +
tRF-NC mimic + − + −
tRF-G130 mimic − + − +
OE-NC + + − −
OE-FBXO7 − − + +
FBXO7
-59 kDa
Col I
-130 kDa
α-SMA
-42 kDa
FN
-260 kDa
β-tubulin
-55 kDa
-180 kDa
-130 kDa
-100 kDa
-70 kDa
-55 kDa
-40 kDa
-180 kDa
-130 kDa
-100 kDa
-70 kDa
-55 kDa
-40 kDa
-180 kDa
-130 kDa
-100 kDa
-70 kDa
-55 kDa
-40 kDa
-180 kDa
-130 kDa
-100 kDa
-70 kDa
-180 kDa
-130 kDa
-100 kDa
-70 kDa
-55 kDa
-40 kDa

Figure. 4I

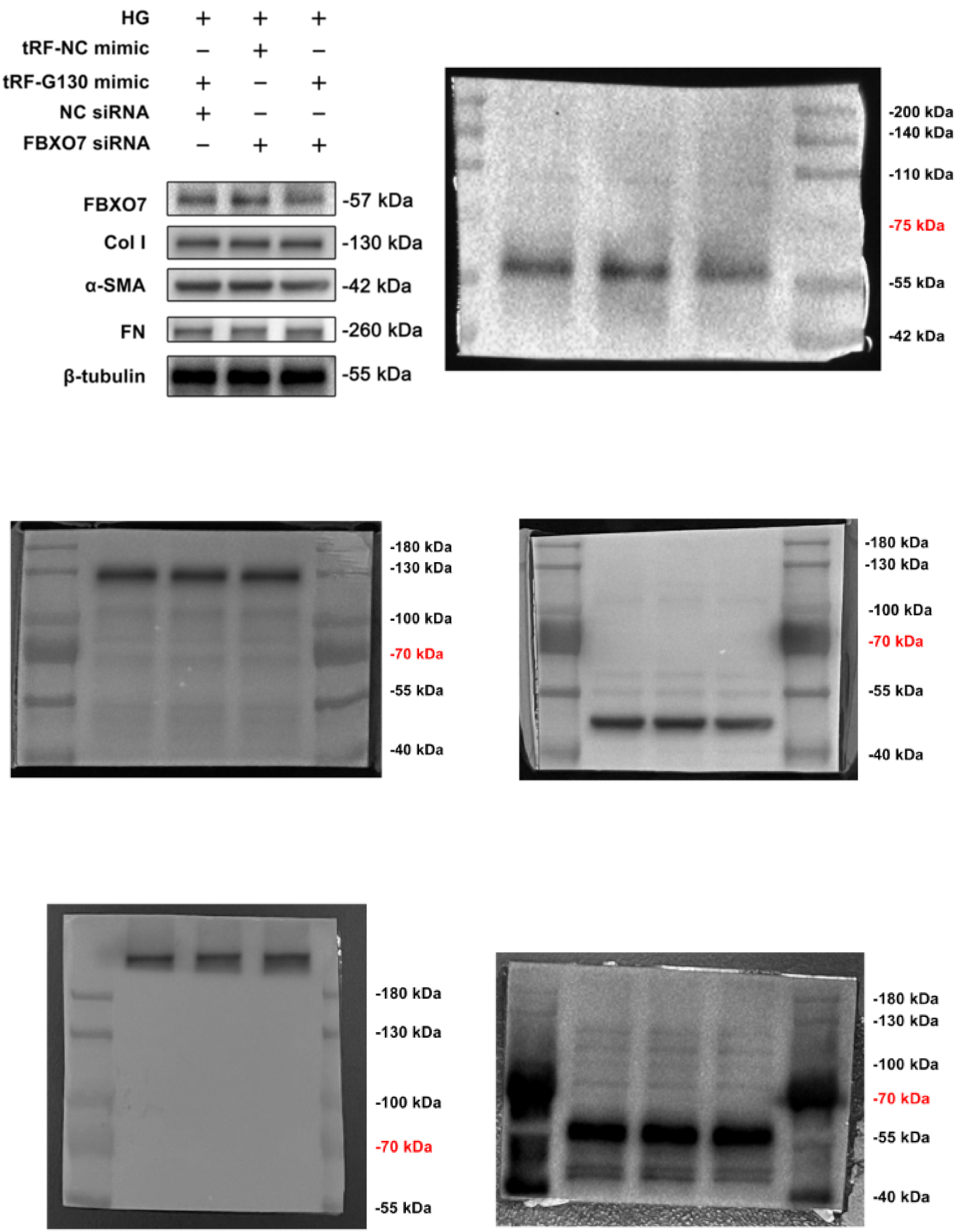
HG + + +
tRF-NC mimic − + −
tRF-G130 mimic + − +
NC siRNA + − −
FBXO7 siRNA − + +
FBXO7
-57 kDa
Col I
-130 kDa
α-SMA
-42 kDa
FN
-260 kDa
β-tubulin
-55 kDa
-200 kDa
-140 kDa
-110 kDa
-75 kDa
-55 kDa
-42 kDa
-180 kDa
-130 kDa
-100 kDa
-70 kDa
-55 kDa
-40 kDa
-180 kDa
-130 kDa
-100 kDa
-70 kDa
-55 kDa
-40 kDa
-180 kDa
-130 kDa
-100 kDa
-70 kDa
-55 kDa
-180 kDa
-130 kDa
-100 kDa
-70 kDa
-55 kDa
-40 kDa

Figure. 5B-C

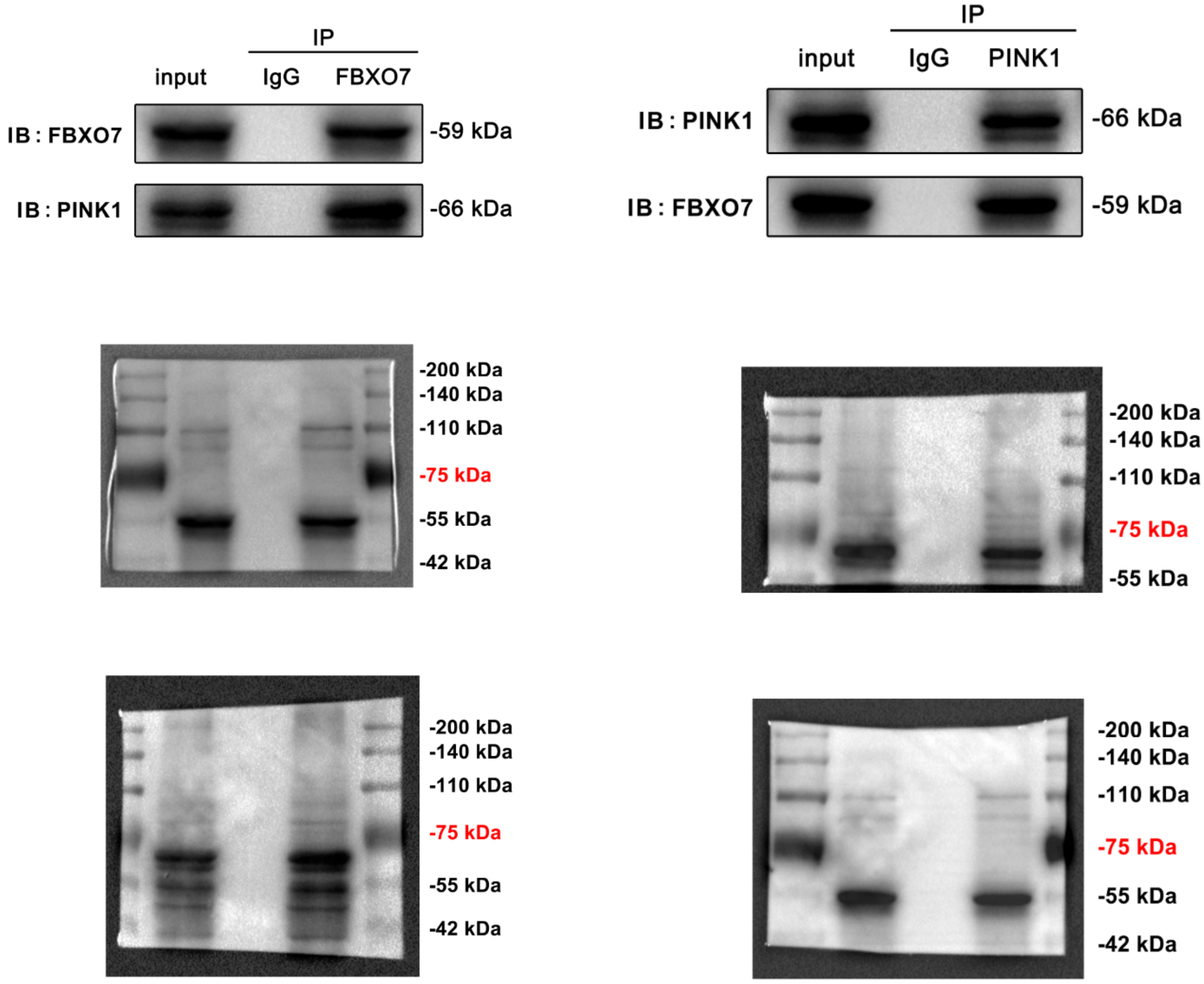

Figure. 5G

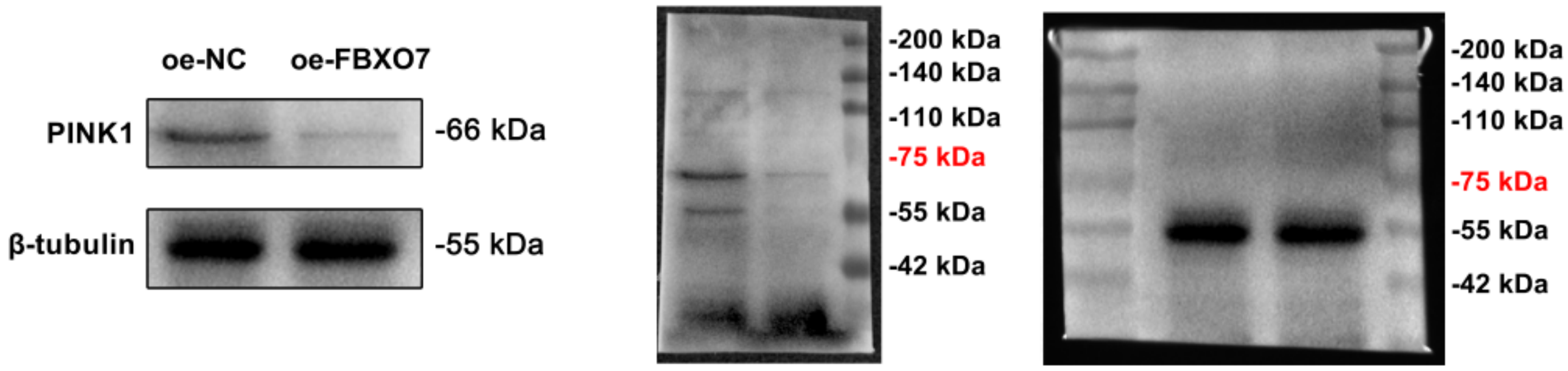

Figure. 5I

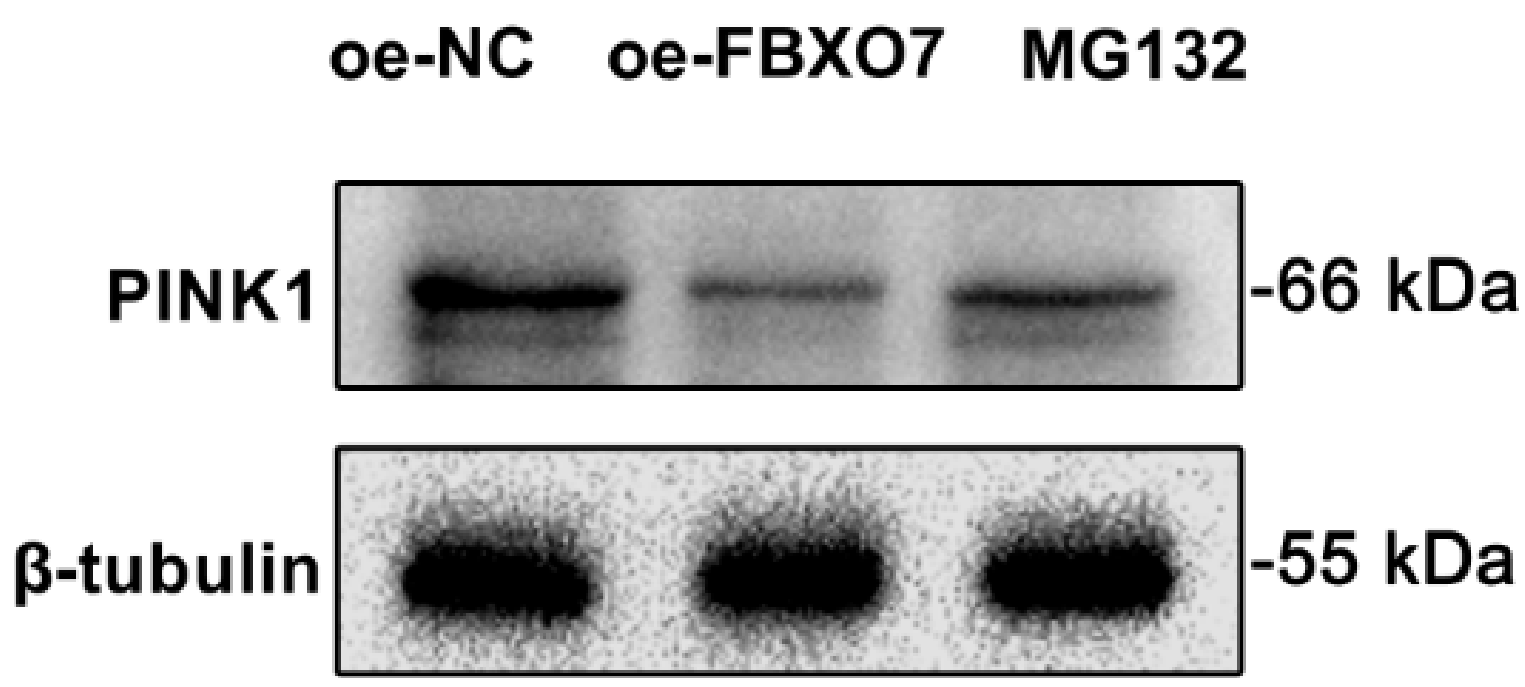

oe-NC
oe-FBXO7
MG132
PINK1
-66 kDa
β-tubulin
-55 kDa

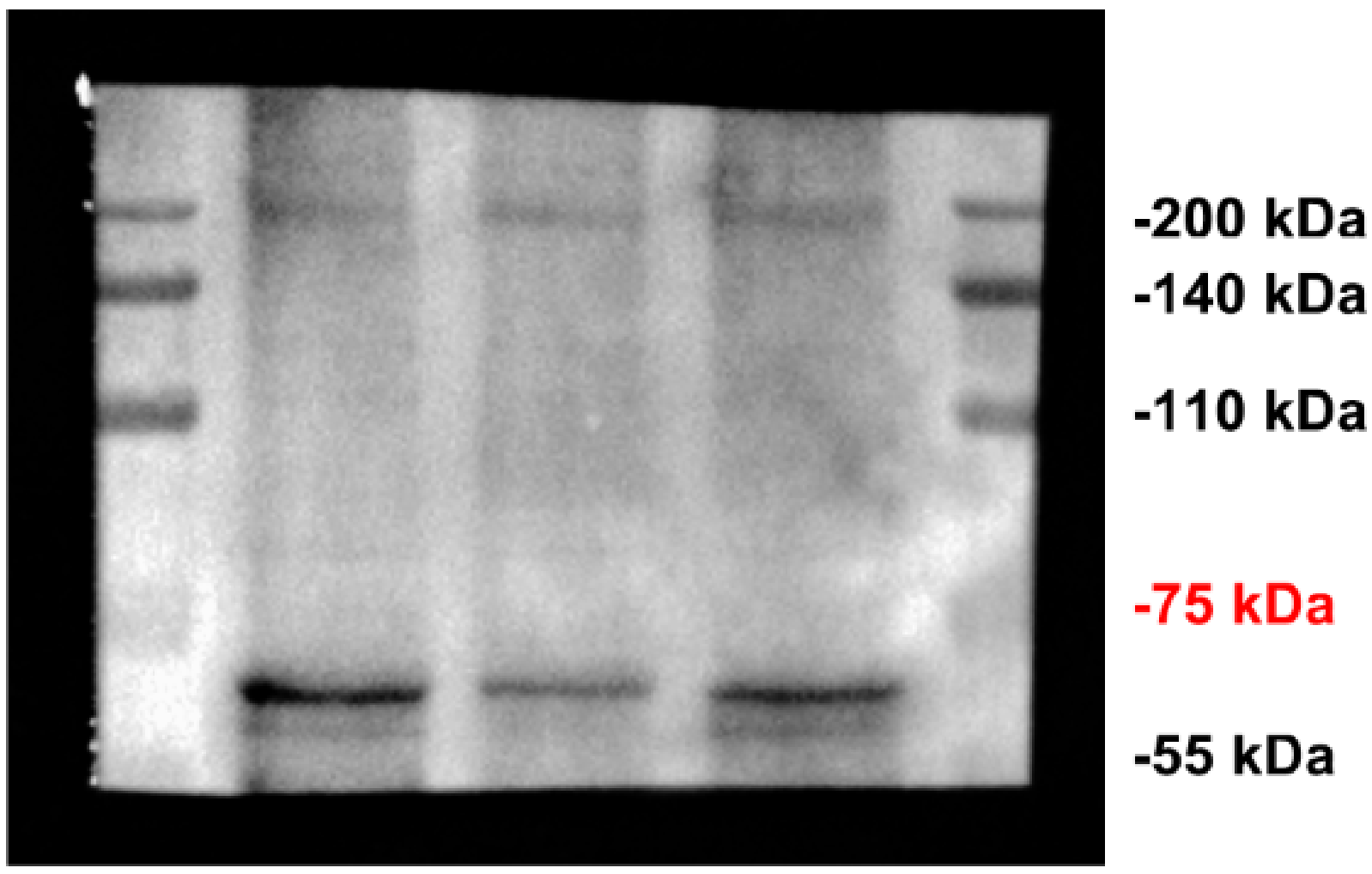

-200 kDa
-140 kDa
-110 kDa
-75 kDa
-55 kDa

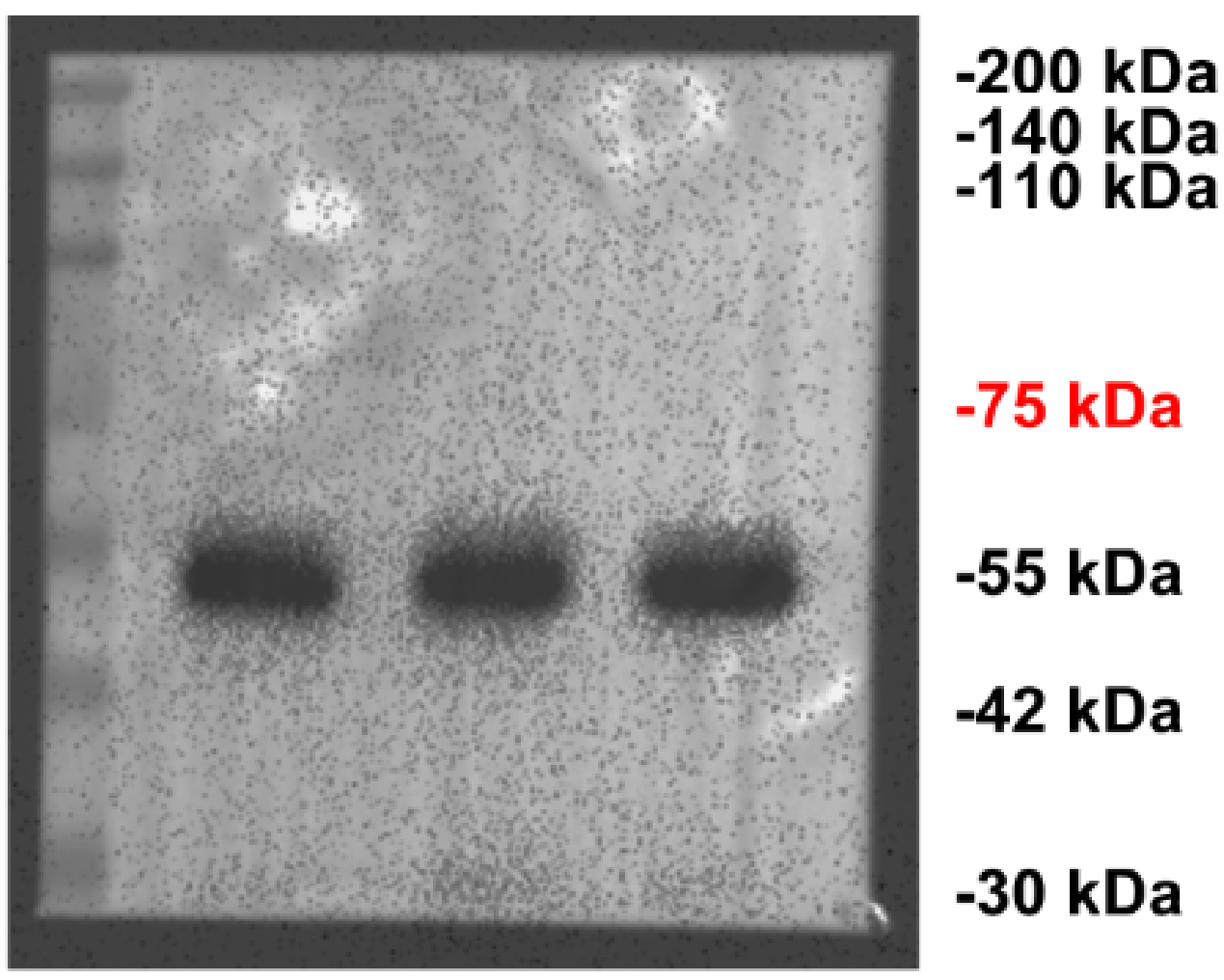

-200 kDa
-140 kDa
-110 kDa
-75 kDa
-55 kDa
-42 kDa
-30 kDa

Figure. 5J

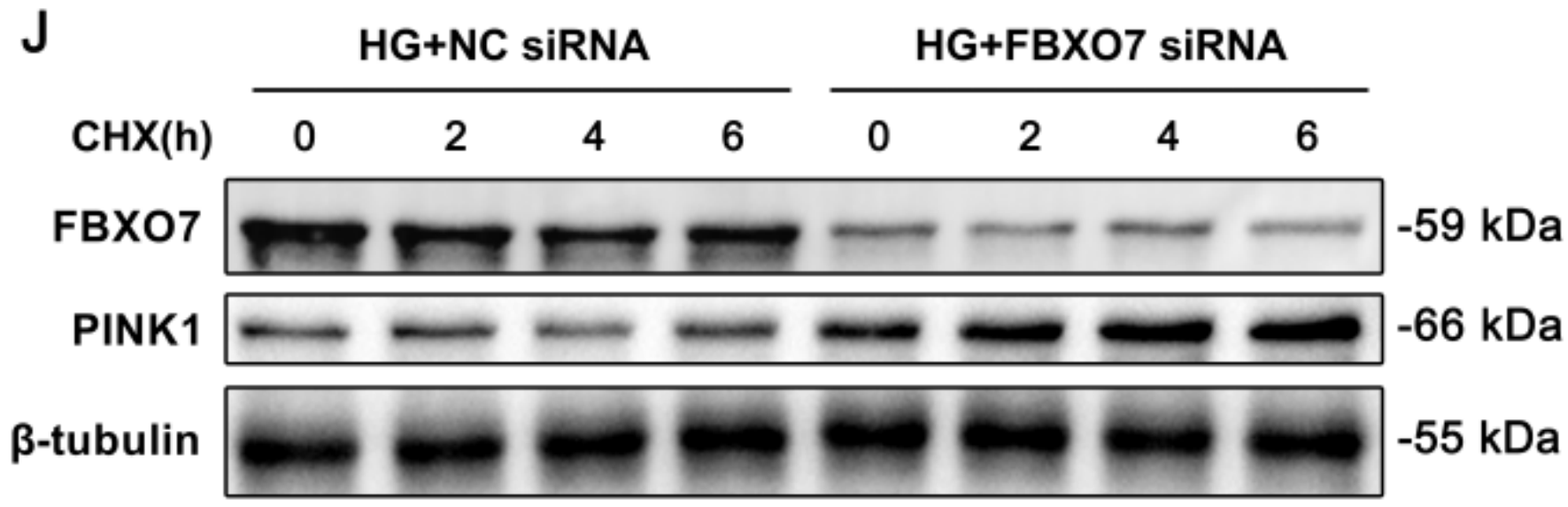
J
HG+NC siRNA
HG+FBXO7 siRNA
CHX(h)
0
2
4
6
0
2
4
6
FBXO7
-59 kDa
PINK1
-66 kDa
β-tubulin
-55 kDa

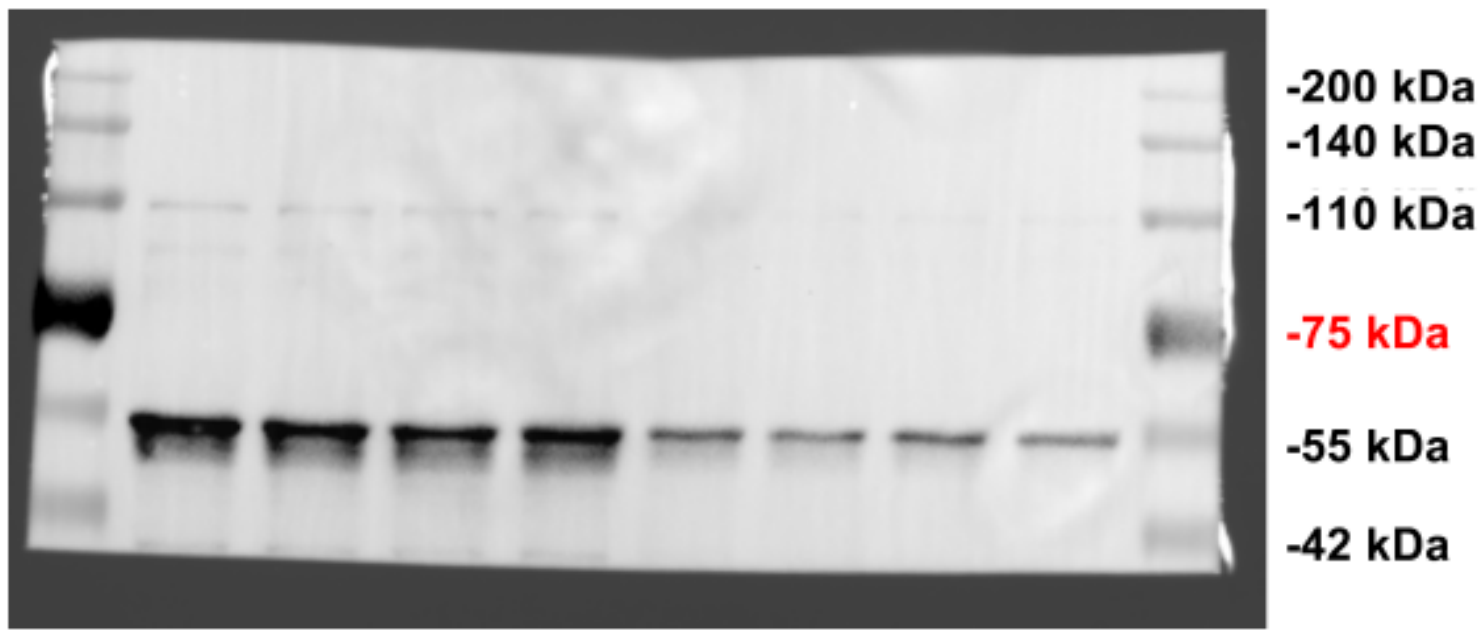
-200 kDa
-140 kDa
-110 kDa
-75 kDa
-55 kDa
-42 kDa

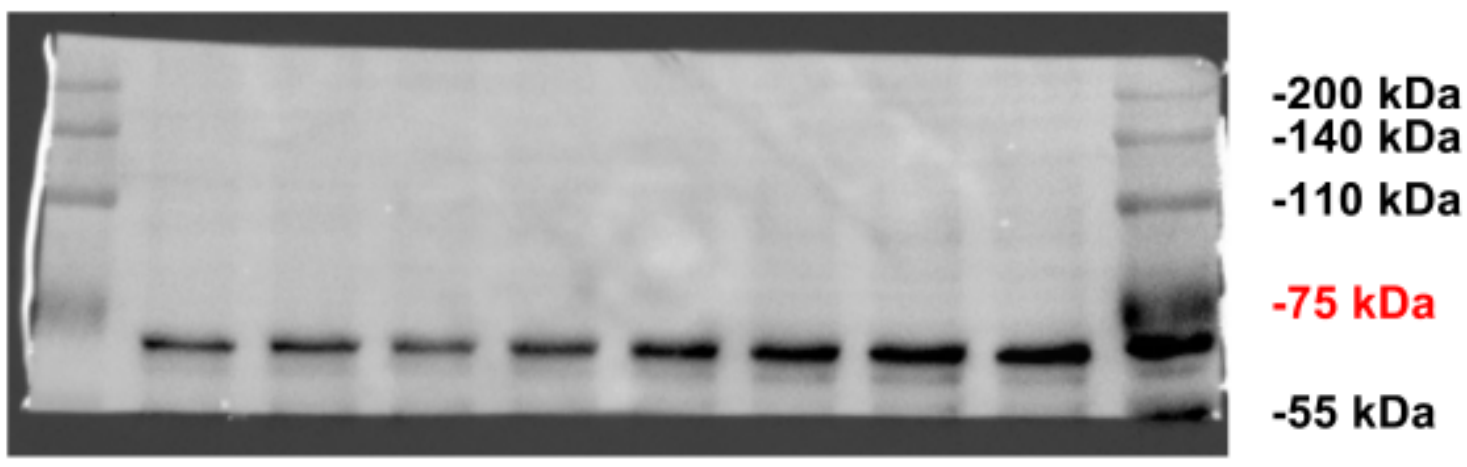
-200 kDa
-140 kDa
-110 kDa
-75 kDa
-55 kDa

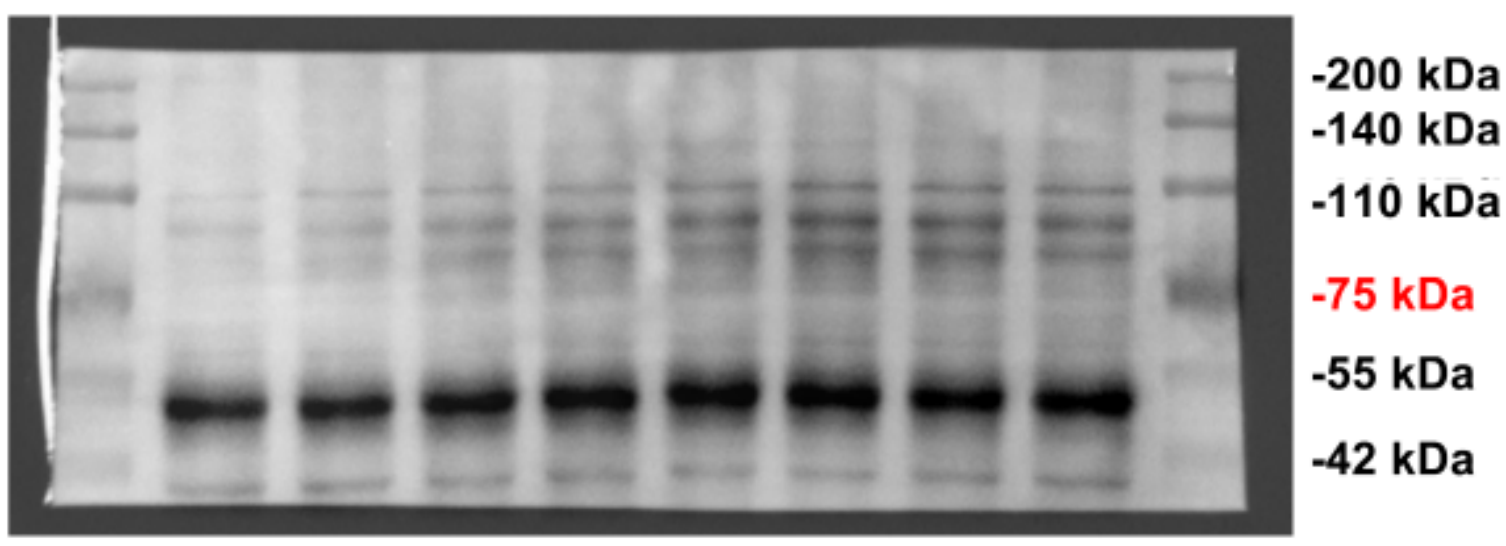
-200 kDa
-140 kDa
-110 kDa
-75 kDa
-55 kDa
-42 kDa

Figure. 5H

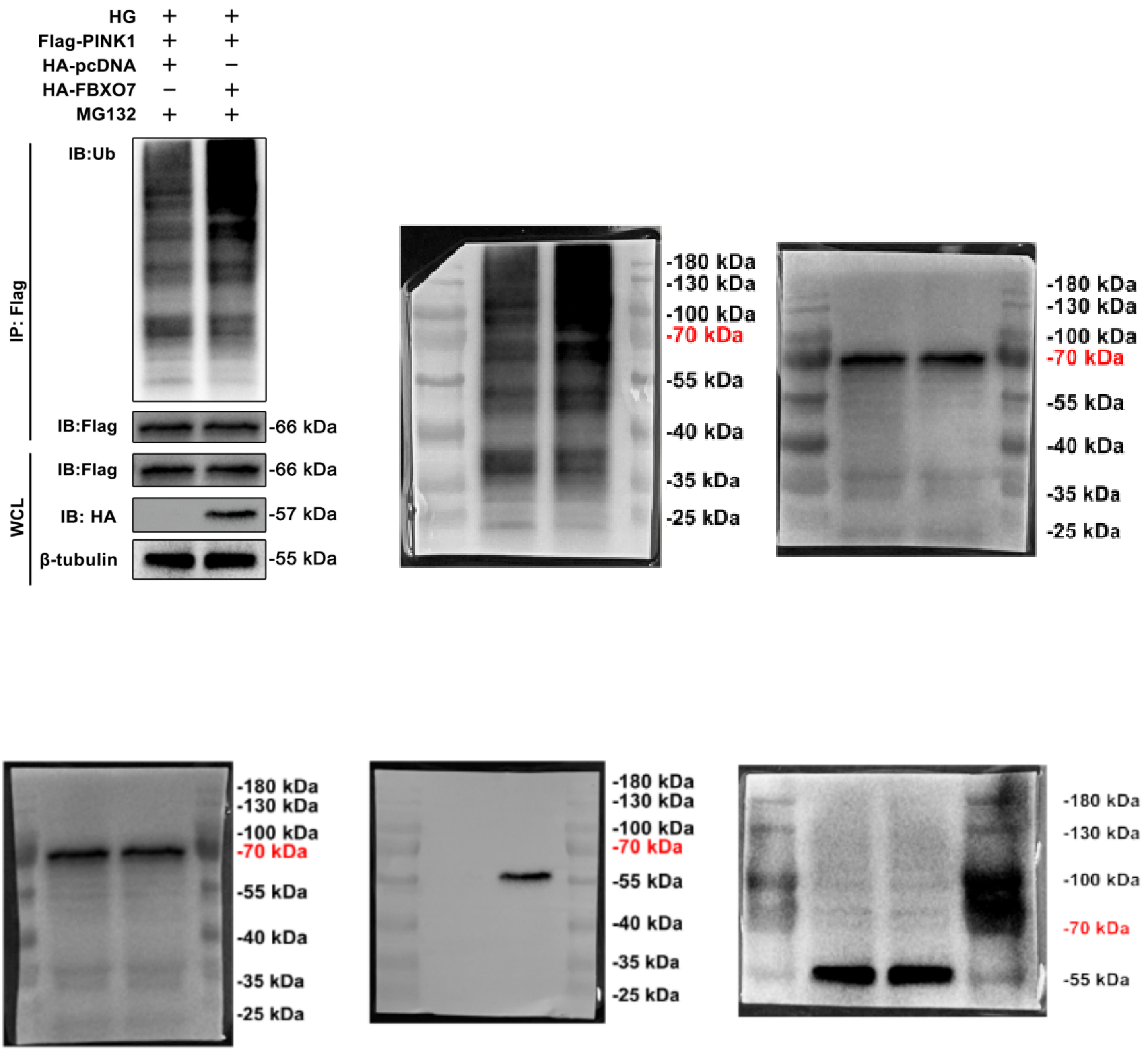
HG + +
Flag-PINK1 + +
HA-pcDNA + −
HA-FBXO7 − +
MG132 + +
IP: Flag
IB:Ub
IB:Flag -66 kDa
WCL
IB:Flag -66 kDa
IB: HA -57 kDa
β-tubulin -55 kDa
-180 kDa
-130 kDa
-100 kDa
-70 kDa
-55 kDa
-40 kDa
-35 kDa
-25 kDa

Figure. 5M

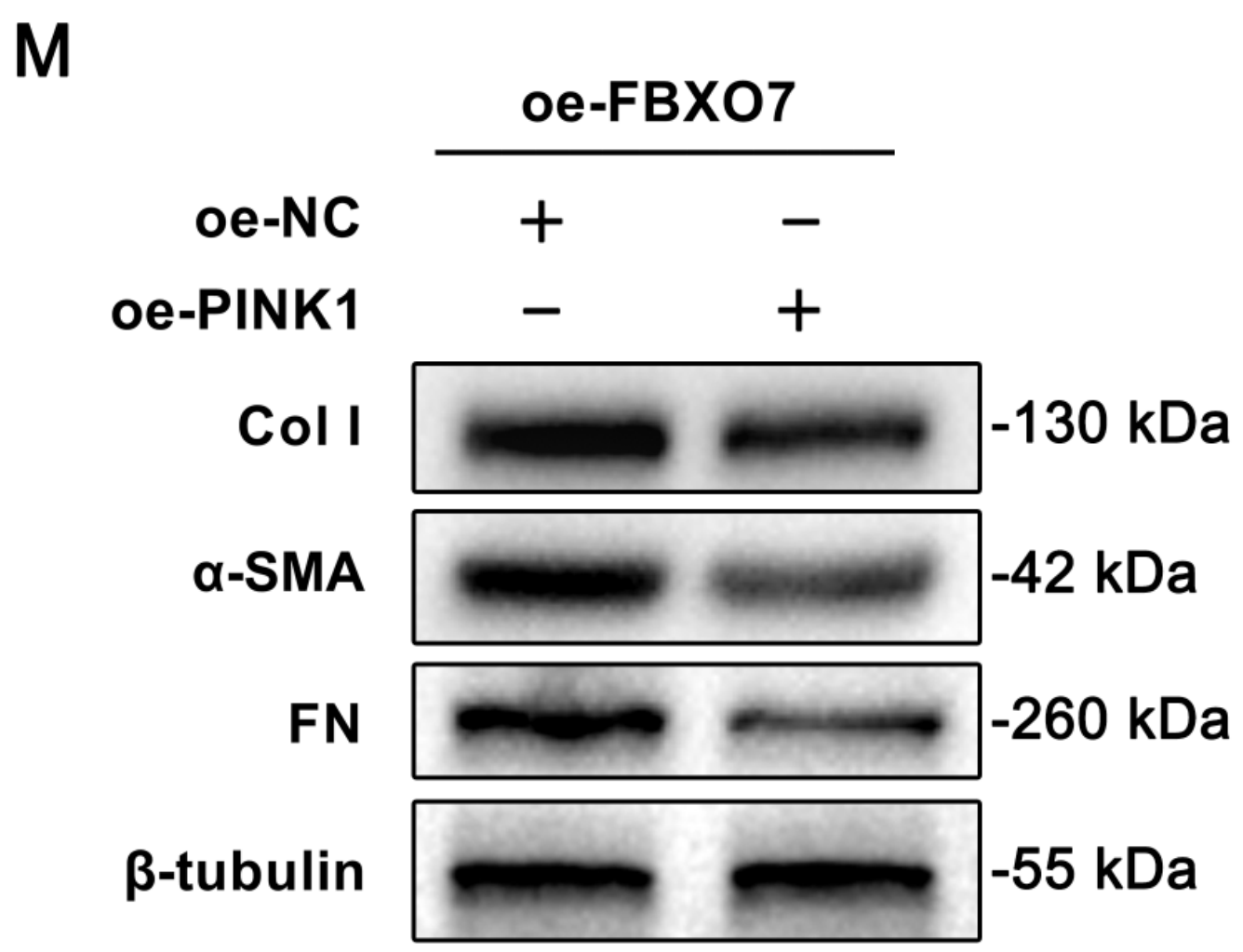
M
oe-FBXO7
oe-NC + −
oe-PINK1 − +
Col I
α-SMA
FN
β-tubulin
-130 kDa
-42 kDa
-260 kDa
-55 kDa

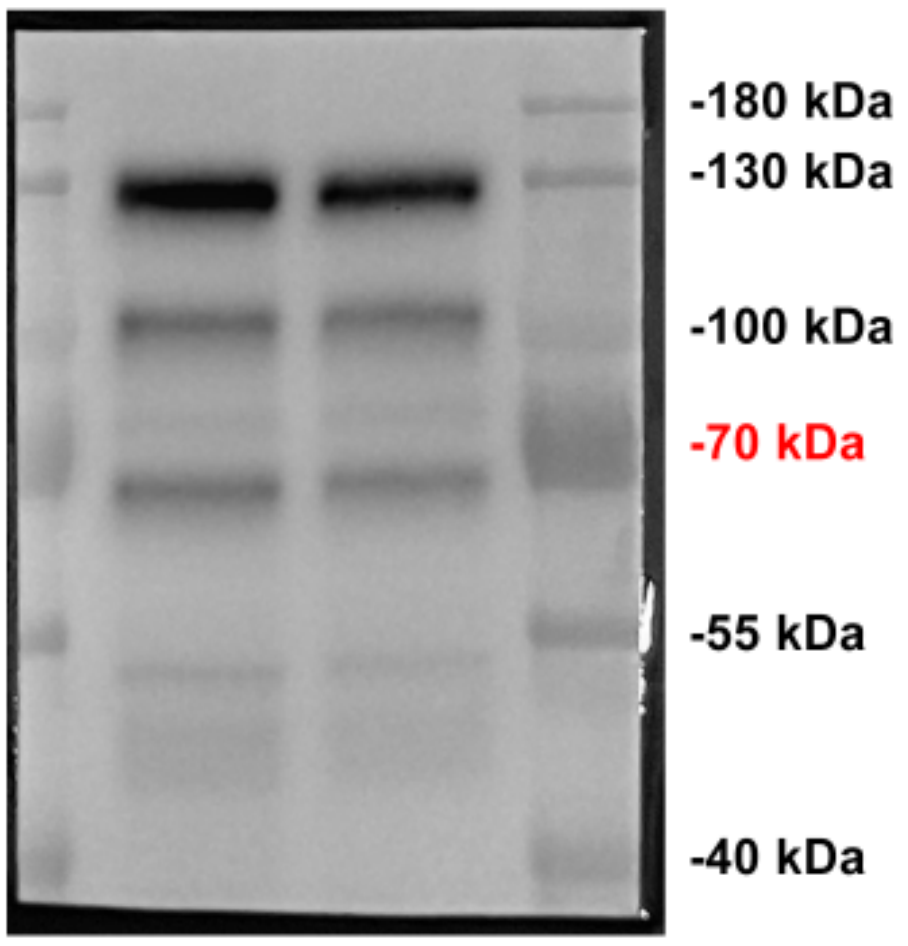
-180 kDa
-130 kDa
-100 kDa
-70 kDa
-55 kDa
-40 kDa

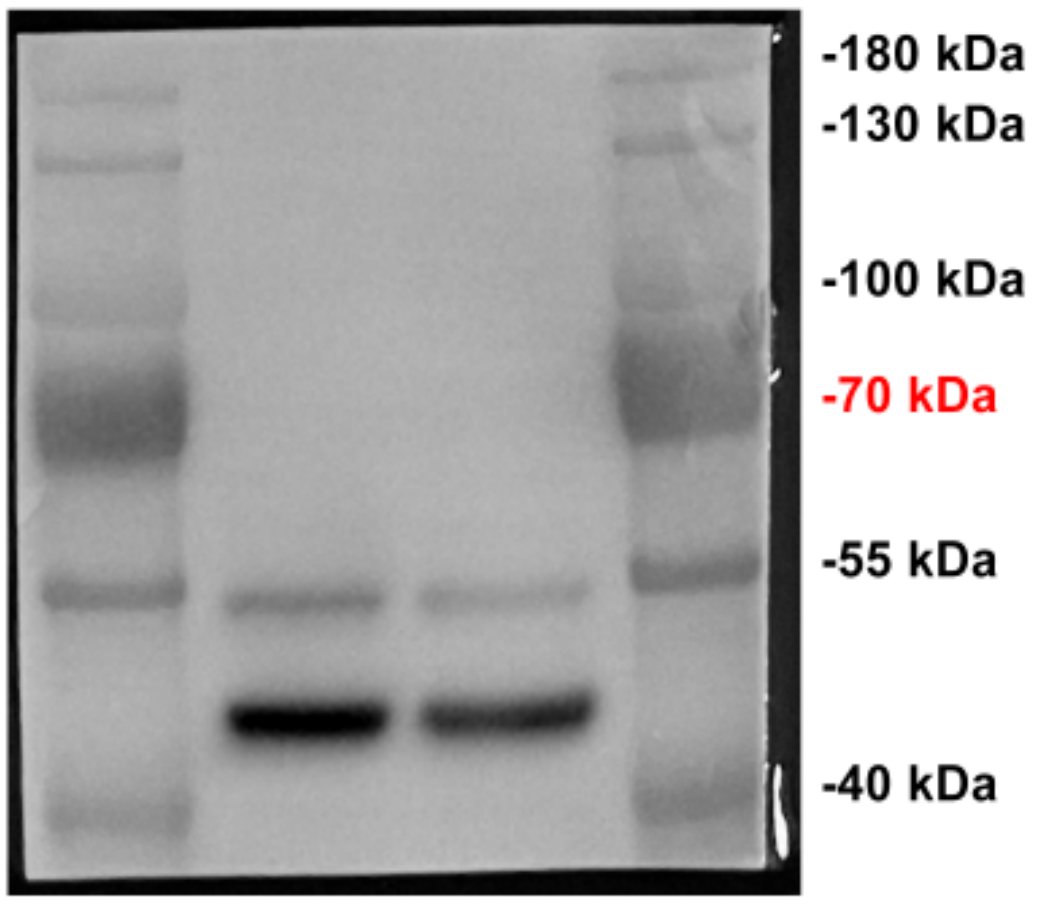
-180 kDa
-130 kDa
-100 kDa
-70 kDa
-55 kDa
-40 kDa

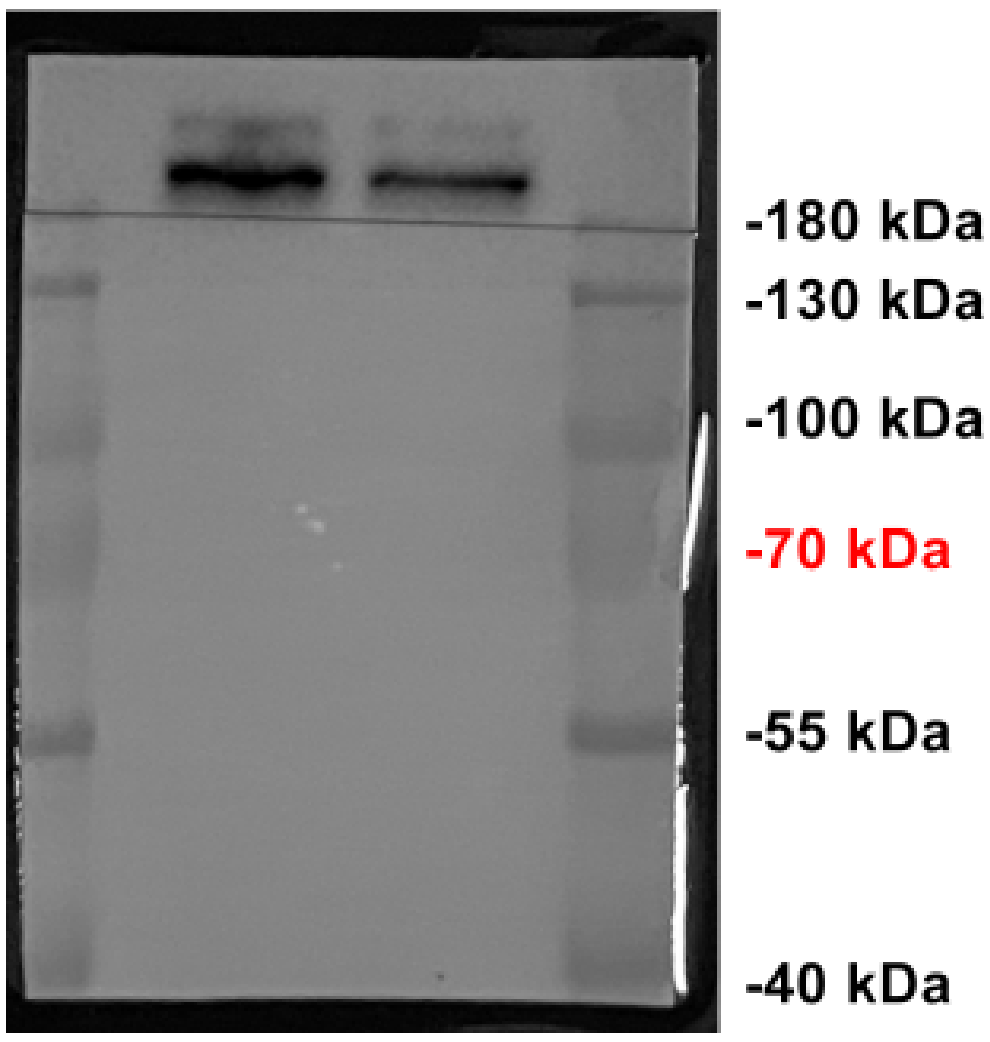
-180 kDa
-130 kDa
-100 kDa
-70 kDa
-55 kDa
-40 kDa

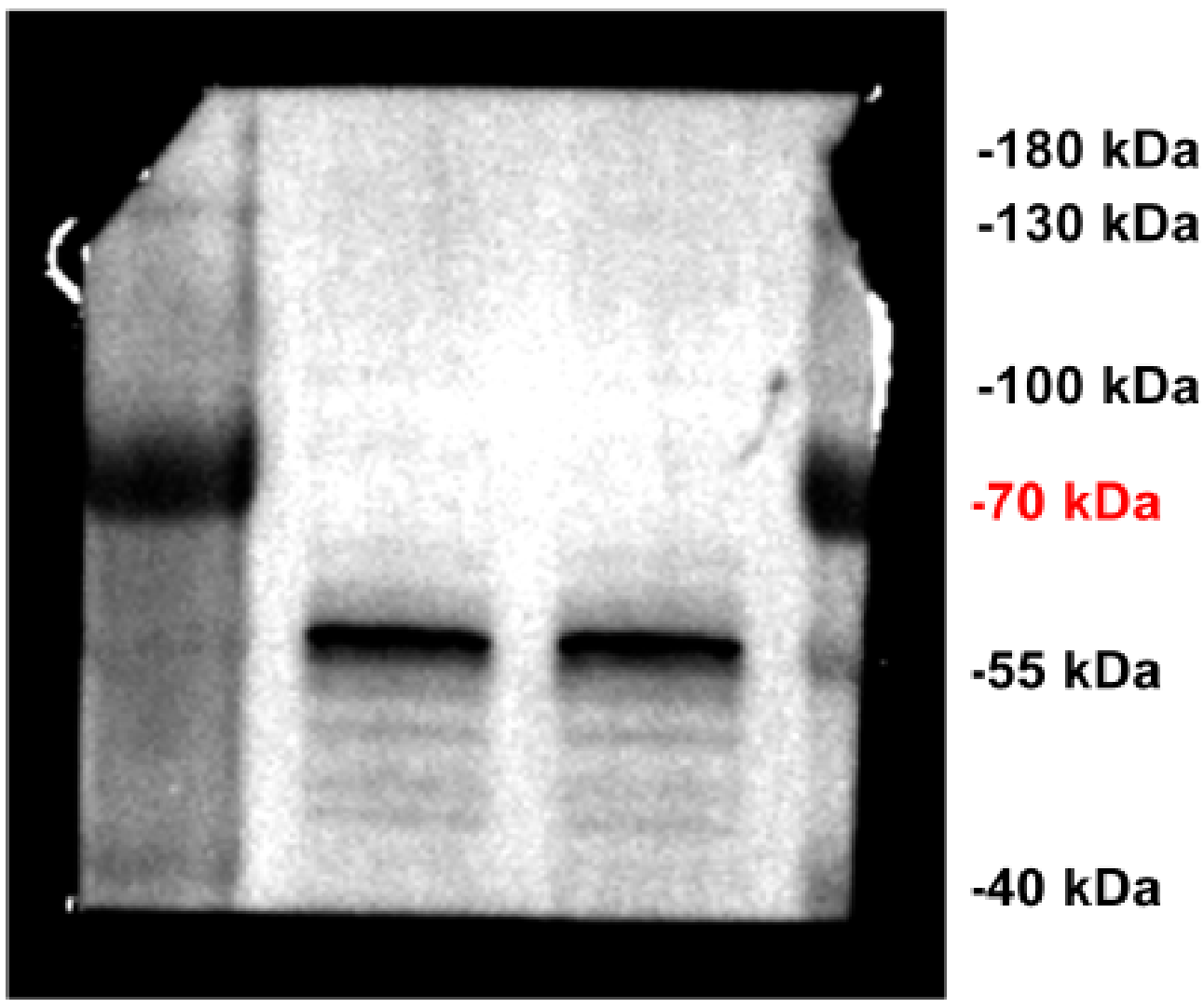
-180 kDa
-130 kDa
-100 kDa
-70 kDa
-55 kDa
-40 kDa

Figure. 5N

N

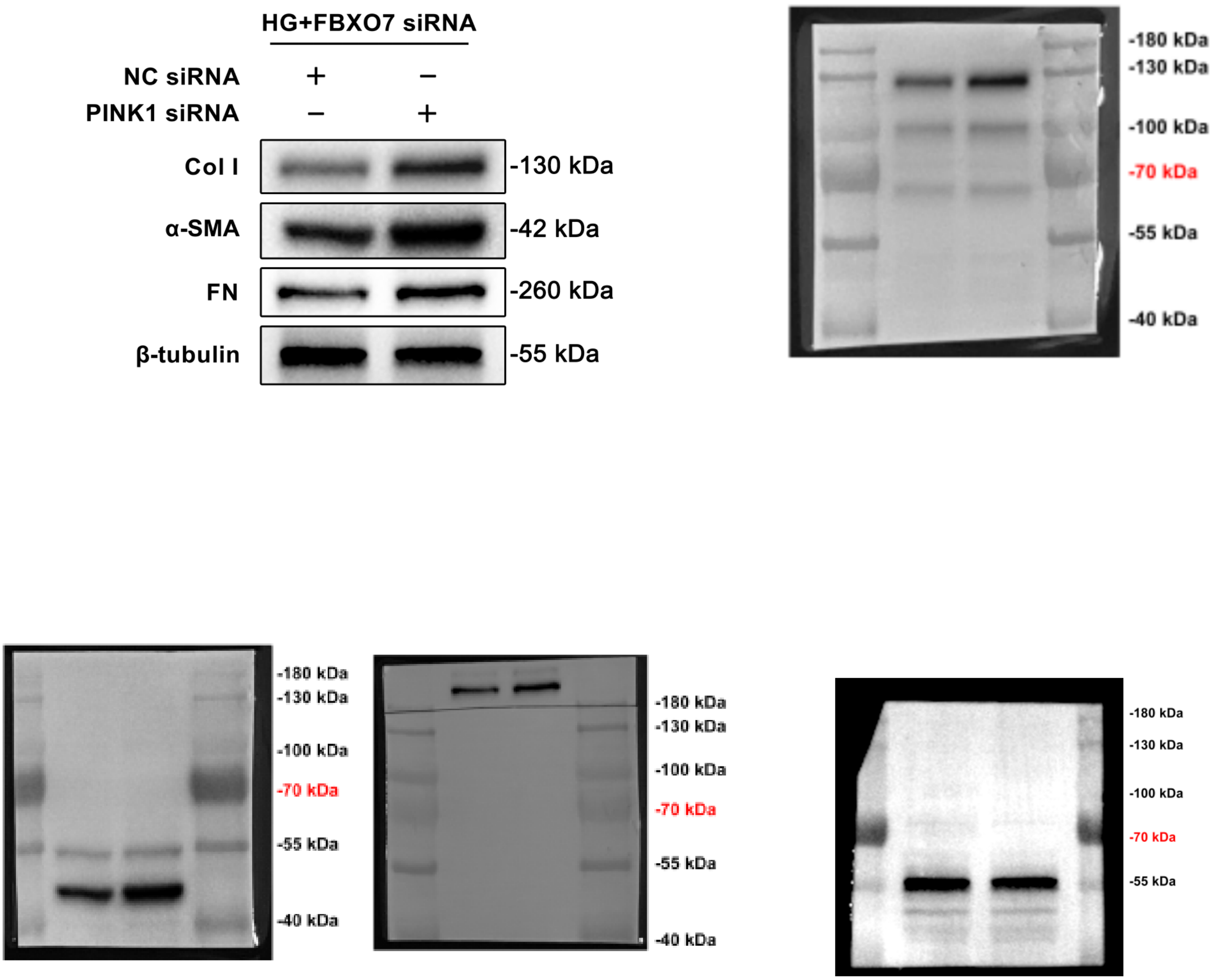
HG+FBXO7 siRNA
NC siRNA + −
PINK1 siRNA − +
Col I
-130 kDa
α-SMA
-42 kDa
FN
-260 kDa
β-tubulin
-55 kDa
-180 kDa
-130 kDa
-100 kDa
-70 kDa
-55 kDa
-40 kDa
-180 kDa
-130 kDa
-100 kDa
-70 kDa
-55 kDa
-40 kDa
-180 kDa
-130 kDa
-100 kDa
-70 kDa
-55 kDa
-40 kDa
-180 kDa
-130 kDa
-100 kDa
-70 kDa
-55 kDa

Figure. 5K

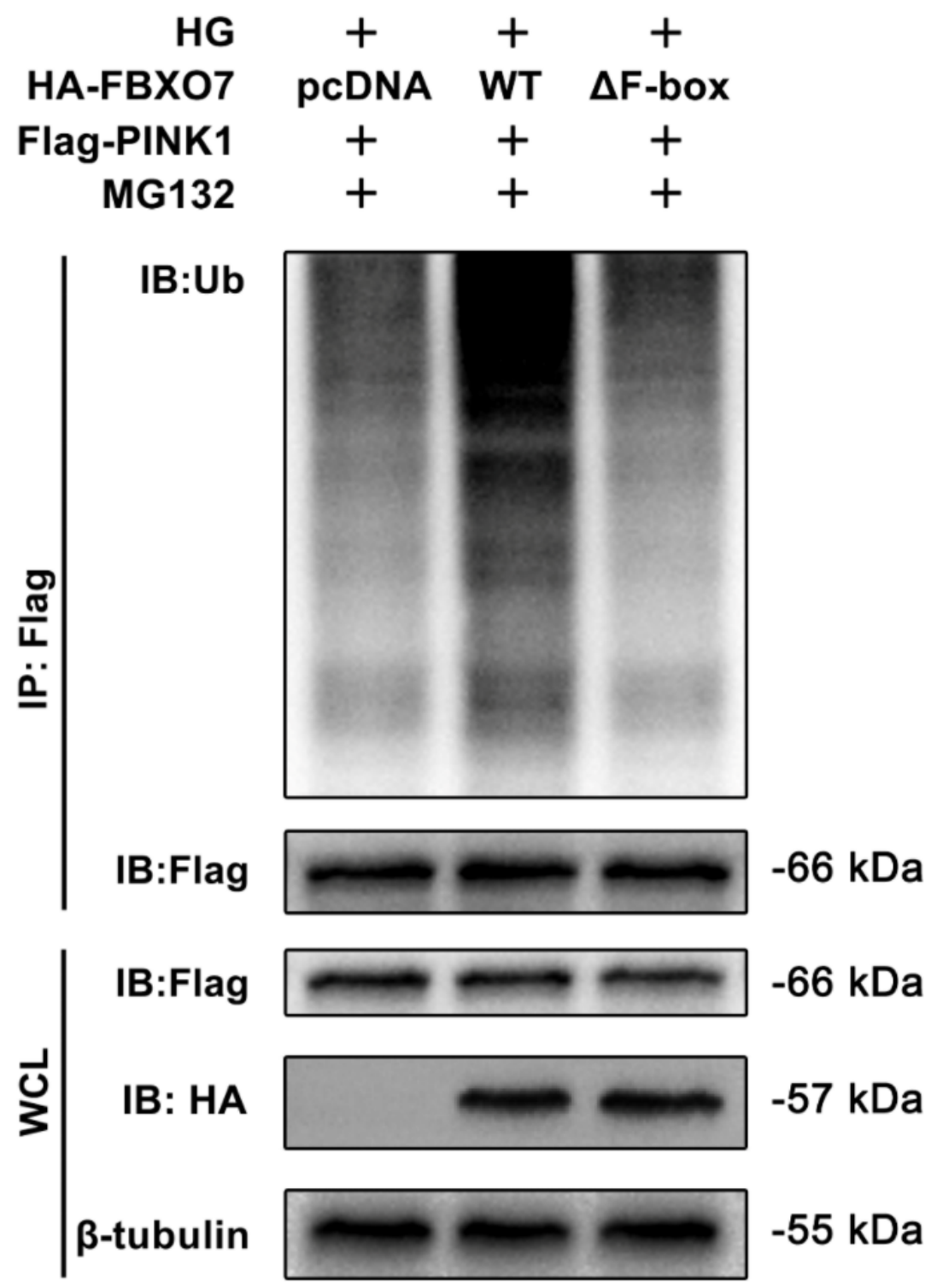
HG + + +
HA-FBXO7 pcDNA WT ΔF-box
Flag-PINK1 + + +
MG132 + + +
IP: Flag
IB:Ub
IB:Flag
-66 kDa
WCL
IB:Flag
-66 kDa
IB: HA
-57 kDa
β-tubulin
-55 kDa

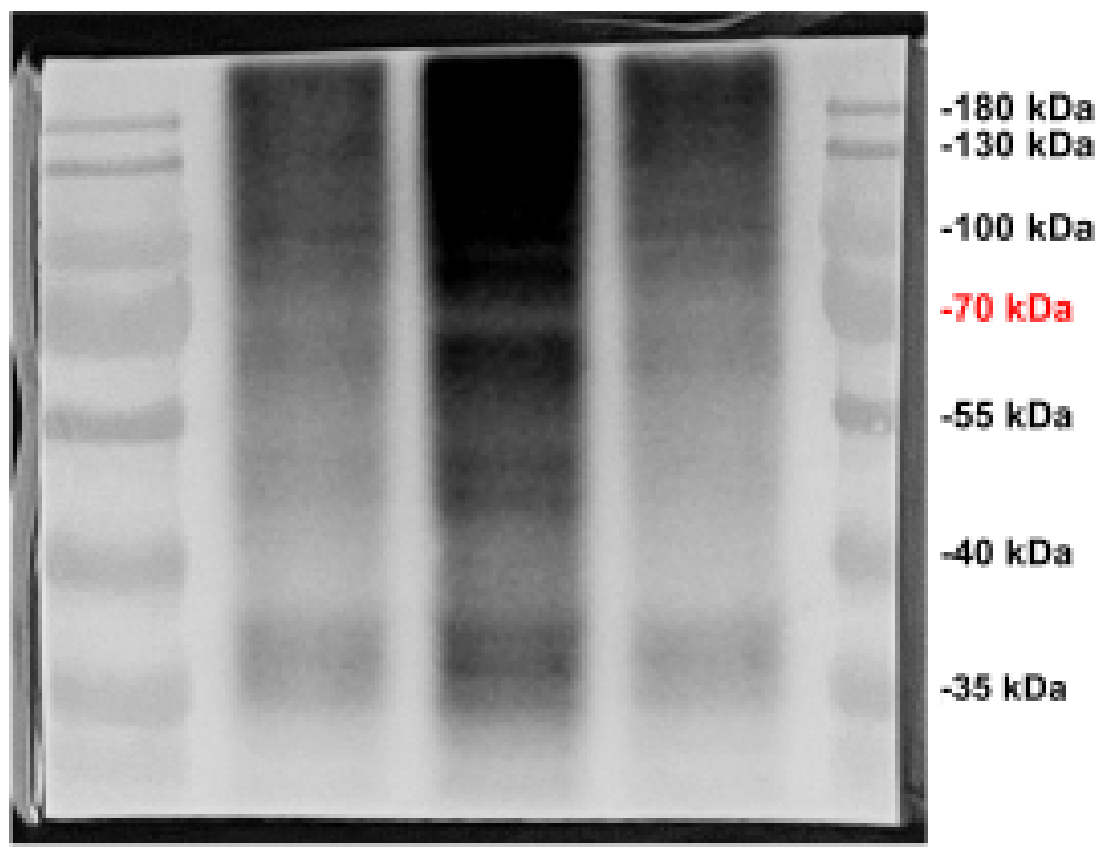
-180 kDa
-130 kDa
-100 kDa
-70 kDa
-55 kDa
-40 kDa
-35 kDa

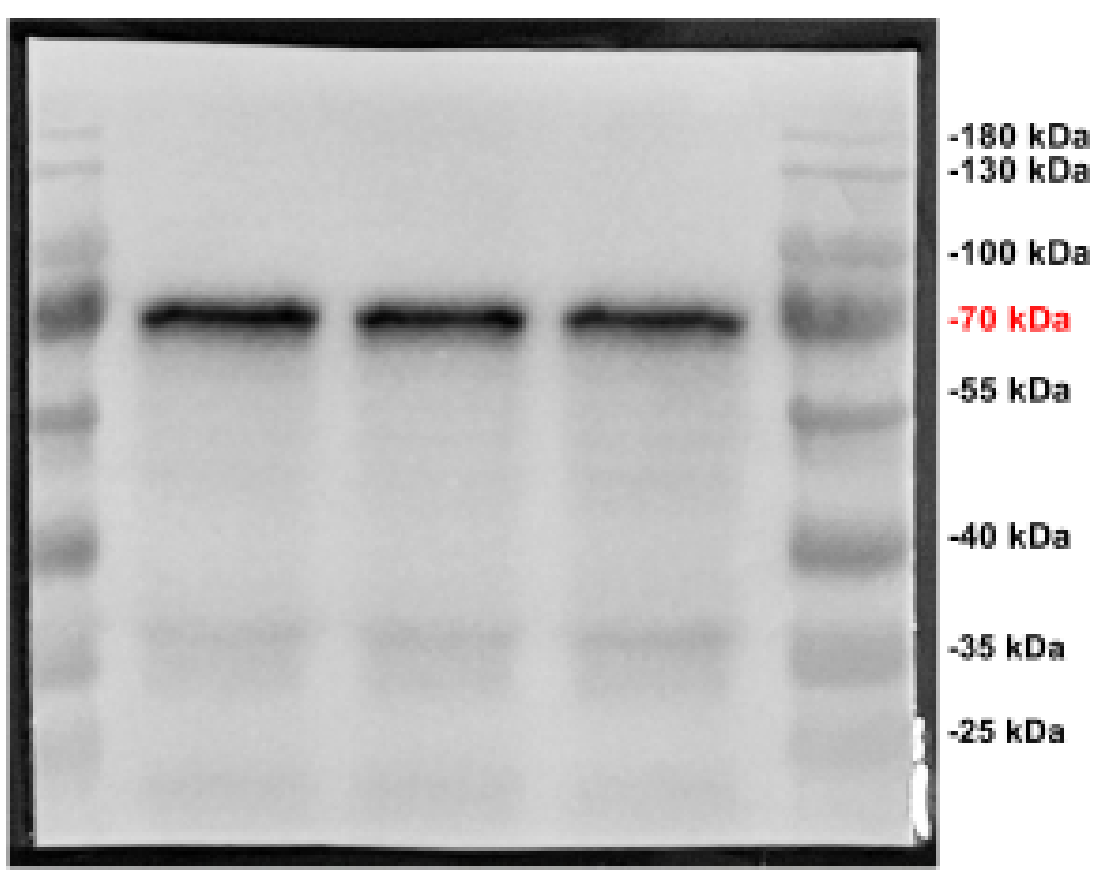
-180 kDa
-130 kDa
-100 kDa
-70 kDa
-55 kDa
-40 kDa
-35 kDa
-25 kDa

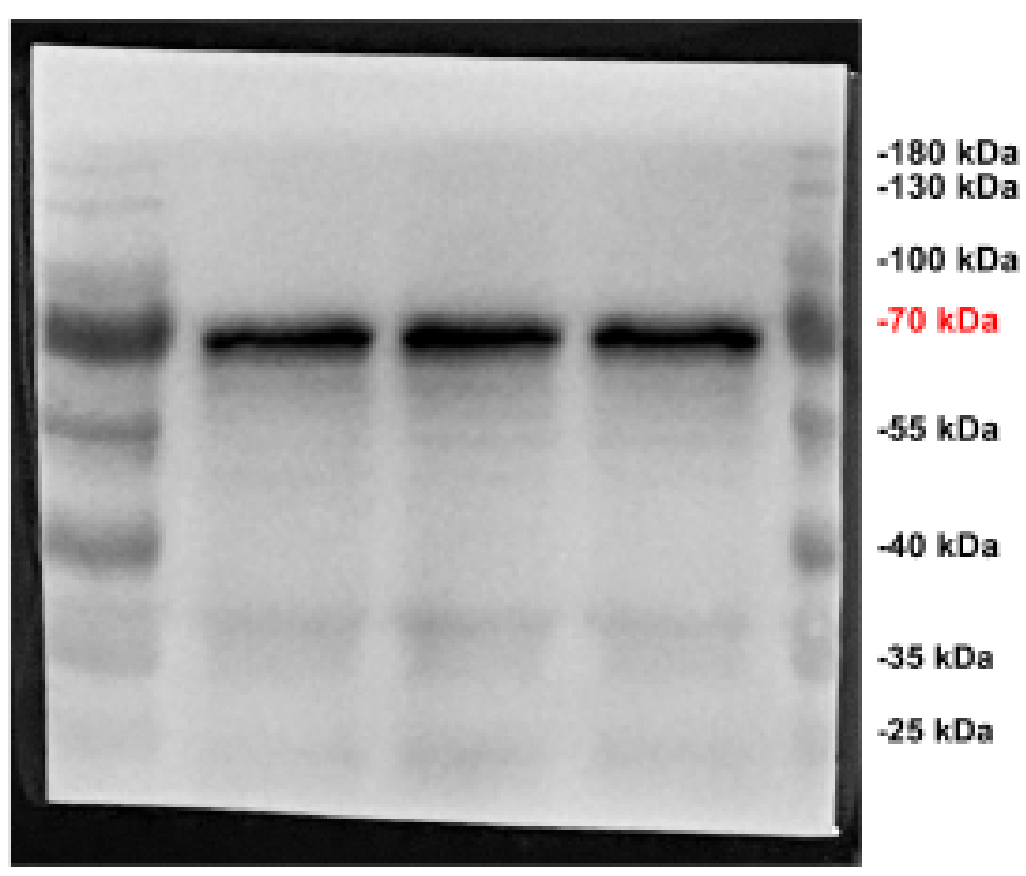
-180 kDa
-130 kDa
-100 kDa
-70 kDa
-55 kDa
-40 kDa
-35 kDa
-25 kDa

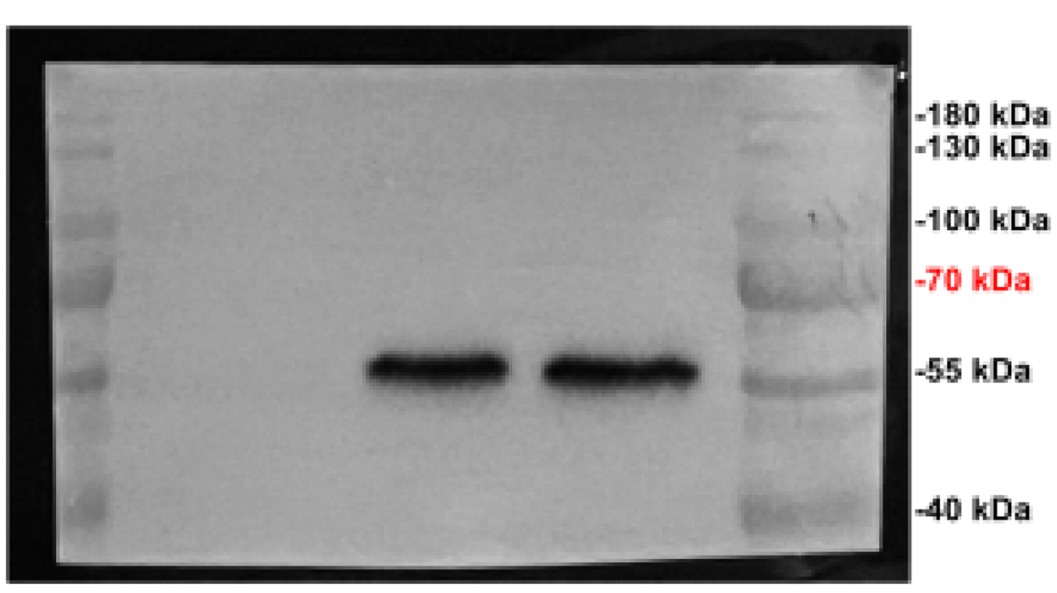
-180 kDa
-130 kDa
-100 kDa
-70 kDa
-55 kDa
-40 kDa

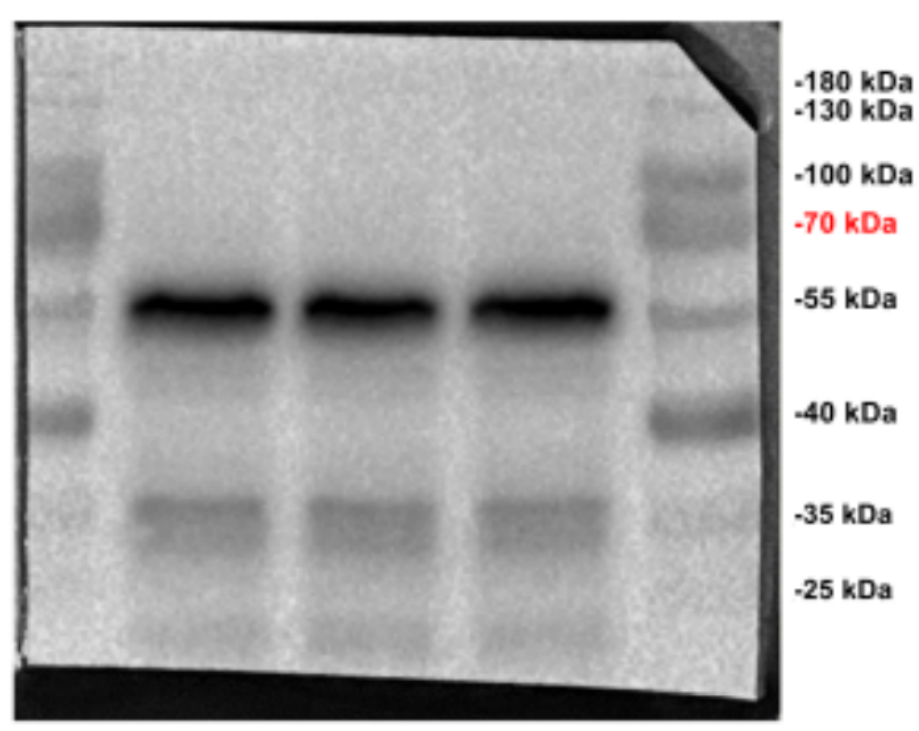
-180 kDa
-130 kDa
-100 kDa
-70 kDa
-55 kDa
-40 kDa
-35 kDa
-25 kDa

Figure. 5L

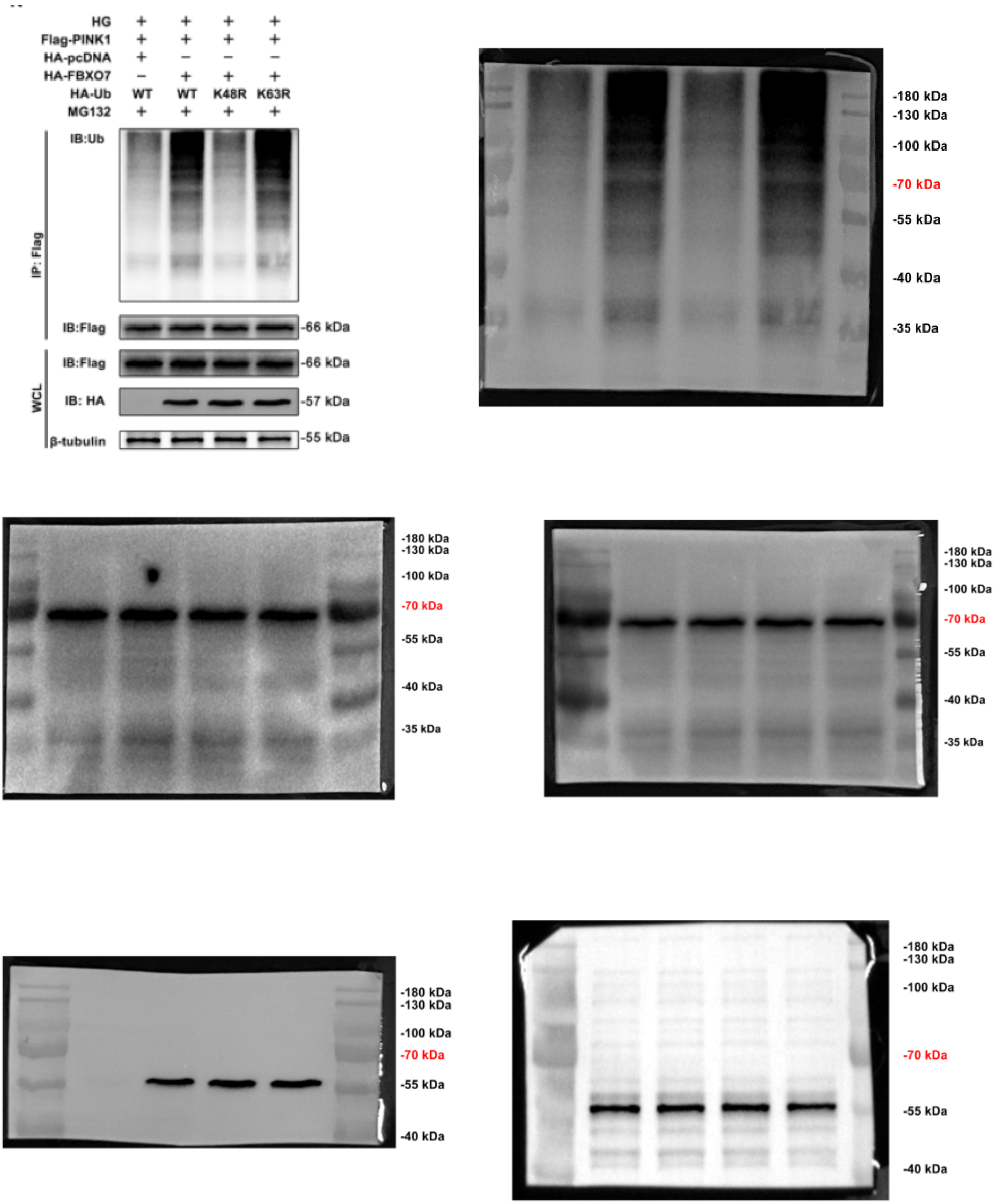

HG + + + +
Flag-PINK1 + + + +
HA-pcDNA + − − −
HA-FBXO7 − + + +
HA-Ub WT WT K48R K63R
MG132 + + + +
IP: Flag
IB:Ub
IB:Flag
-66 kDa
WCL
IB:Flag
-66 kDa
IB: HA
-57 kDa
β-tubulin
-55 kDa
-180 kDa
-130 kDa
-100 kDa
-70 kDa
-55 kDa
-40 kDa
-35 kDa
-180 kDa
-130 kDa
-100 kDa
-70 kDa
-55 kDa
-40 kDa
-35 kDa
-180 kDa
-130 kDa
-100 kDa
-70 kDa
-55 kDa
-40 kDa
-35 kDa
-180 kDa
-130 kDa
-100 kDa
-70 kDa
-55 kDa
-40 kDa
-180 kDa
-130 kDa
-100 kDa
-70 kDa
-55 kDa
-40 kDa

Figure. 6A

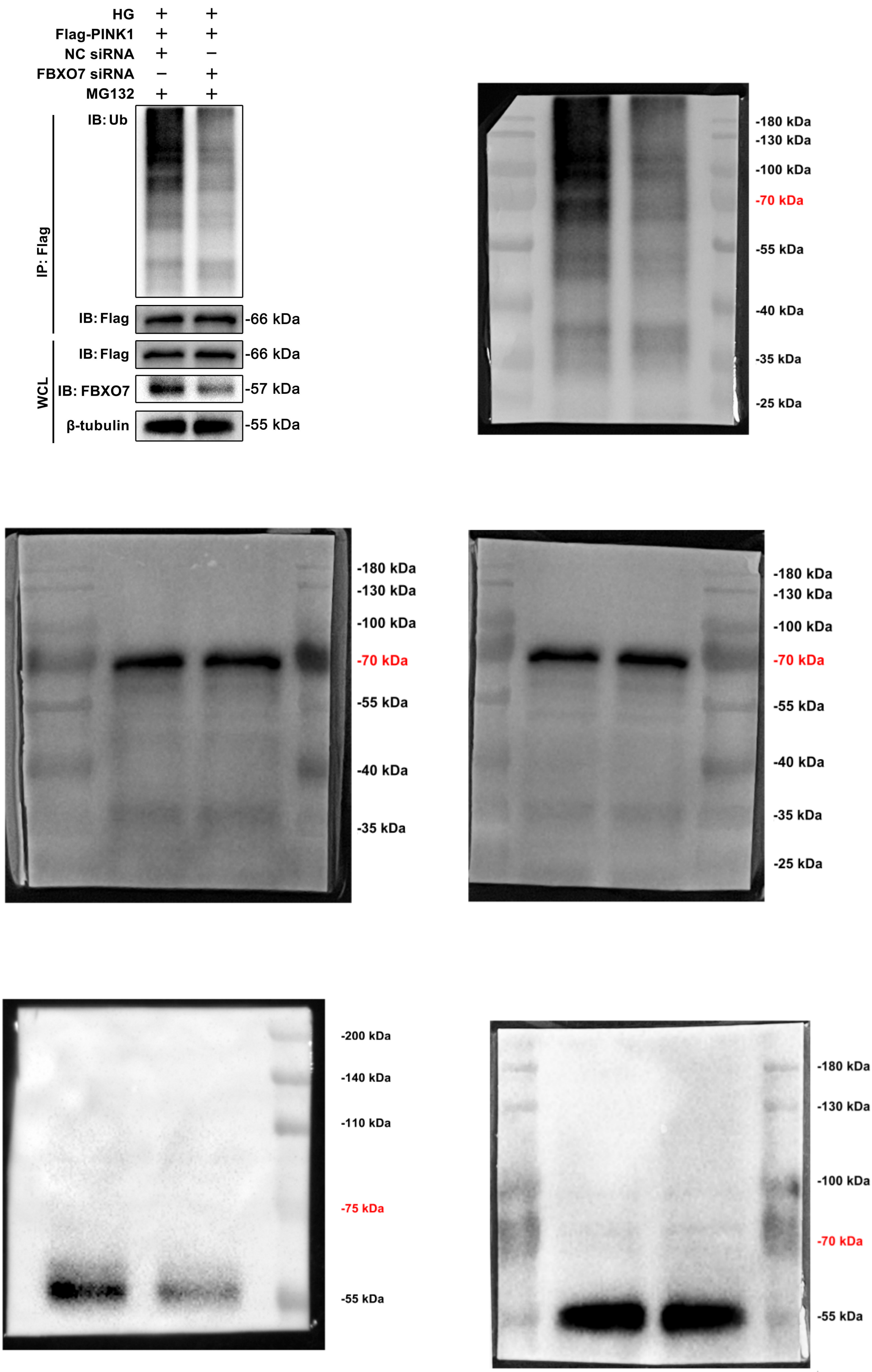
HG + +
Flag-PINK1 + +
NC siRNA + −
FBXO7 siRNA − +
MG132 + +
IP: Flag
IB: Ub
IB: Flag
-66 kDa
WCL
IB: Flag
-66 kDa
IB: FBXO7
-57 kDa
β-tubulin
-55 kDa
-180 kDa
-130 kDa
-100 kDa
-70 kDa
-55 kDa
-40 kDa
-35 kDa
-25 kDa
-180 kDa
-130 kDa
-100 kDa
-70 kDa
-55 kDa
-40 kDa
-35 kDa
-180 kDa
-130 kDa
-100 kDa
-70 kDa
-55 kDa
-40 kDa
-35 kDa
-25 kDa
-200 kDa
-140 kDa
-110 kDa
-75 kDa
-55 kDa
-180 kDa
-130 kDa
-100 kDa
-70 kDa
-55 kDa

Figure. 6B

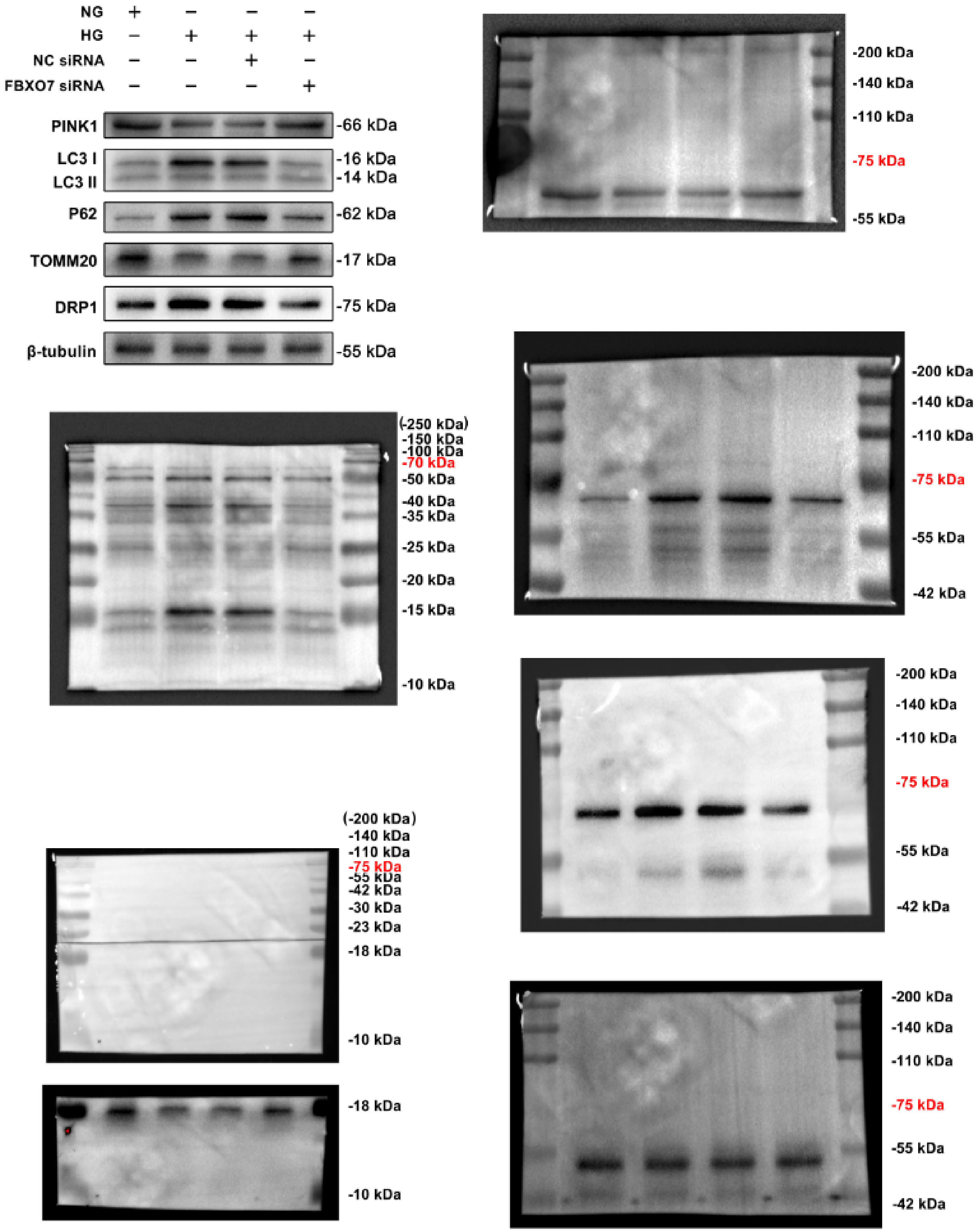
NG + − − −
HG − + + +
NC siRNA − − + −
FBXO7 siRNA − − − +
PINK1
-66 kDa
LC3 I
LC3 II
-16 kDa
-14 kDa
P62
-62 kDa
TOMM20
-17 kDa
DRP1
-75 kDa
β-tubulin
-55 kDa
-200 kDa
-140 kDa
-110 kDa
-75 kDa
-55 kDa
(-250 kDa)
-150 kDa
-100 kDa
-70 kDa
-50 kDa
-40 kDa
-35 kDa
-25 kDa
-20 kDa
-15 kDa
-10 kDa
-200 kDa
-140 kDa
-110 kDa
-75 kDa
-55 kDa
-42 kDa
-200 kDa
-140 kDa
-110 kDa
-75 kDa
-55 kDa
-42 kDa
(-200 kDa)
-140 kDa
-110 kDa
-75 kDa
-55 kDa
-42 kDa
-30 kDa
-23 kDa
-18 kDa
-10 kDa
-18 kDa
-10 kDa
-200 kDa
-140 kDa
-110 kDa
-75 kDa
-55 kDa
-42 kDa

Figure. 6G

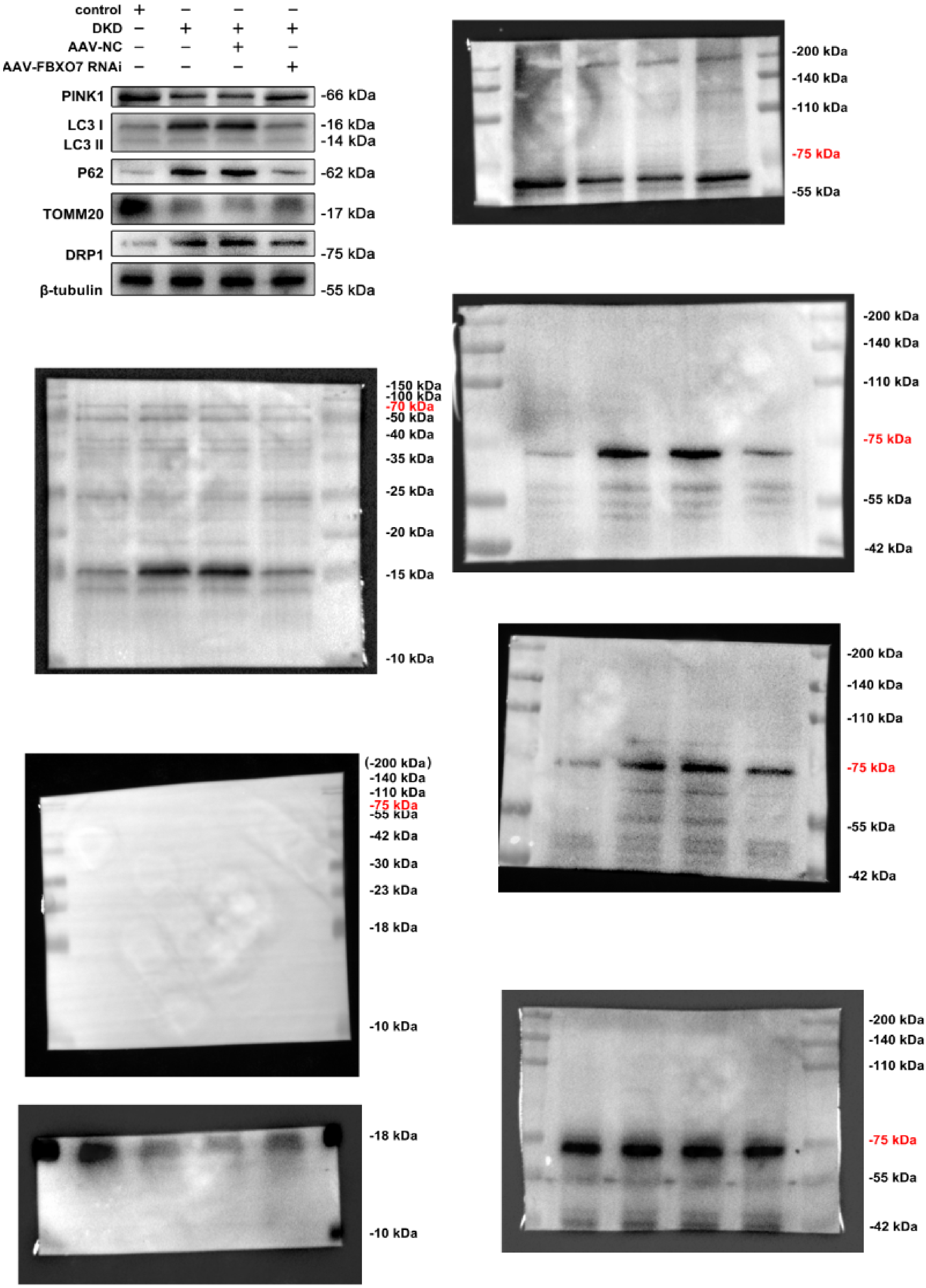
control + − − −
DKD − + + +
AAV-NC − − + −
AAV-FBXO7 RNAi − − − +
PINK1
-66 kDa
LC3 I
LC3 II
-16 kDa
-14 kDa
P62
-62 kDa
TOMM20
-17 kDa
DRP1
-75 kDa
β-tubulin
-55 kDa
-200 kDa
-140 kDa
-110 kDa
-75 kDa
-55 kDa
-150 kDa
-100 kDa
-70 kDa
-50 kDa
-40 kDa
-35 kDa
-25 kDa
-20 kDa
-15 kDa
-10 kDa
-200 kDa
-140 kDa
-110 kDa
-75 kDa
-55 kDa
-42 kDa
(-200 kDa)
-140 kDa
-110 kDa
-75 kDa
-55 kDa
-42 kDa
-30 kDa
-23 kDa
-18 kDa
-10 kDa
-200 kDa
-140 kDa
-110 kDa
-75 kDa
-55 kDa
-42 kDa
-18 kDa
-10 kDa
-200 kDa
-140 kDa
-110 kDa
-75 kDa
-55 kDa
-42 kDa

Figure. 7B

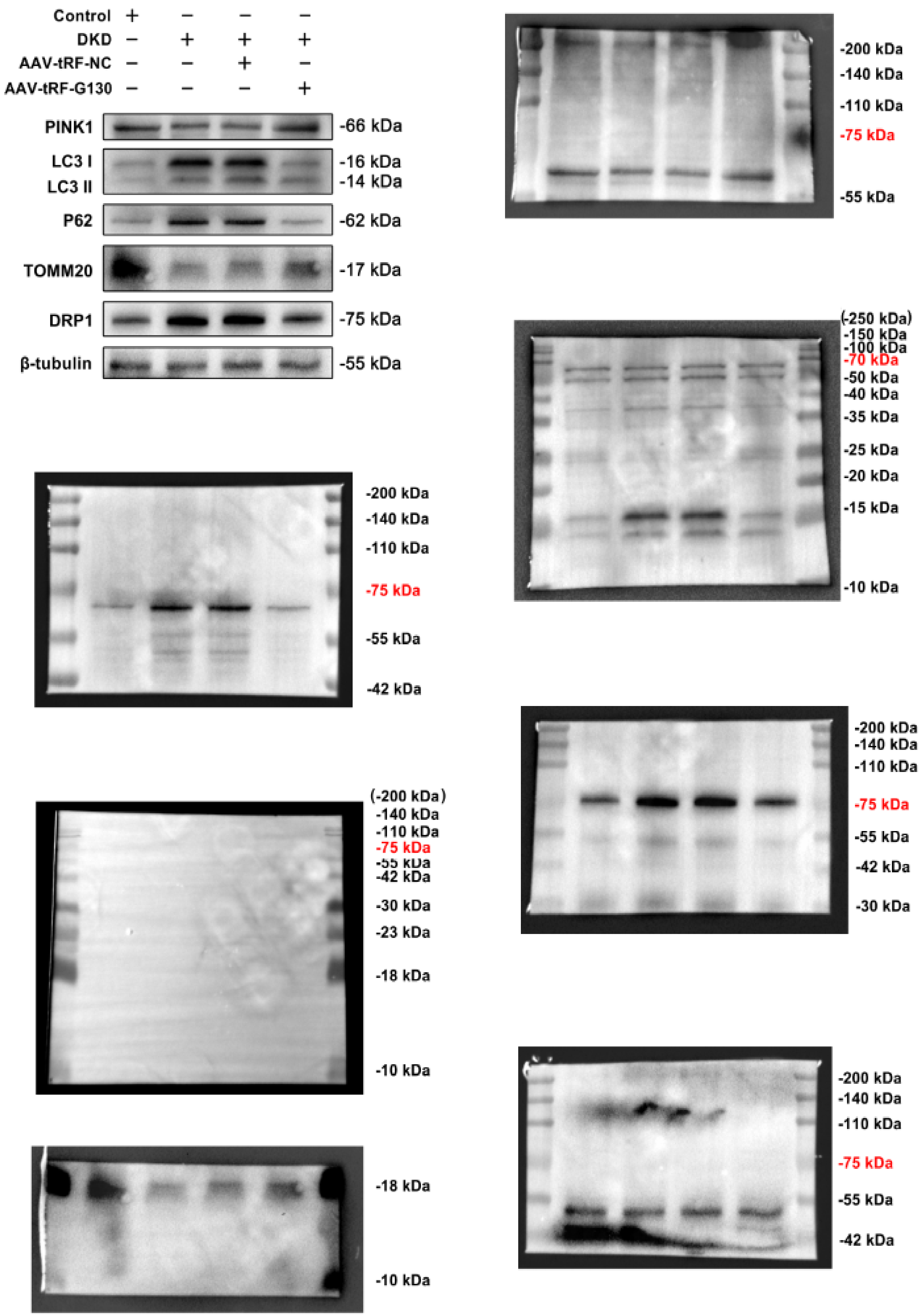

Control + − − −
DKD − + + +
AAV-tRF-NC − − + −
AAV-tRF-G130 − − − +
PINK1
-66 kDa
LC3 I
LC3 II
-16 kDa
-14 kDa
P62
-62 kDa
TOMM20
-17 kDa
DRP1
-75 kDa
β-tubulin
-55 kDa
-200 kDa
-140 kDa
-110 kDa
-75 kDa
-55 kDa
(-250 kDa)
-150 kDa
-100 kDa
-70 kDa
-50 kDa
-40 kDa
-35 kDa
-25 kDa
-20 kDa
-15 kDa
-10 kDa
-200 kDa
-140 kDa
-110 kDa
-75 kDa
-55 kDa
-42 kDa
-200 kDa
-140 kDa
-110 kDa
-75 kDa
-55 kDa
-42 kDa
-30 kDa
(-200 kDa)
-140 kDa
-110 kDa
-75 kDa
-42 kDa
-30 kDa
-23 kDa
-18 kDa
-10 kDa
-200 kDa
-140 kDa
-110 kDa
-75 kDa
-55 kDa
-42 kDa
-18 kDa
-10 kDa

Figure. 7D

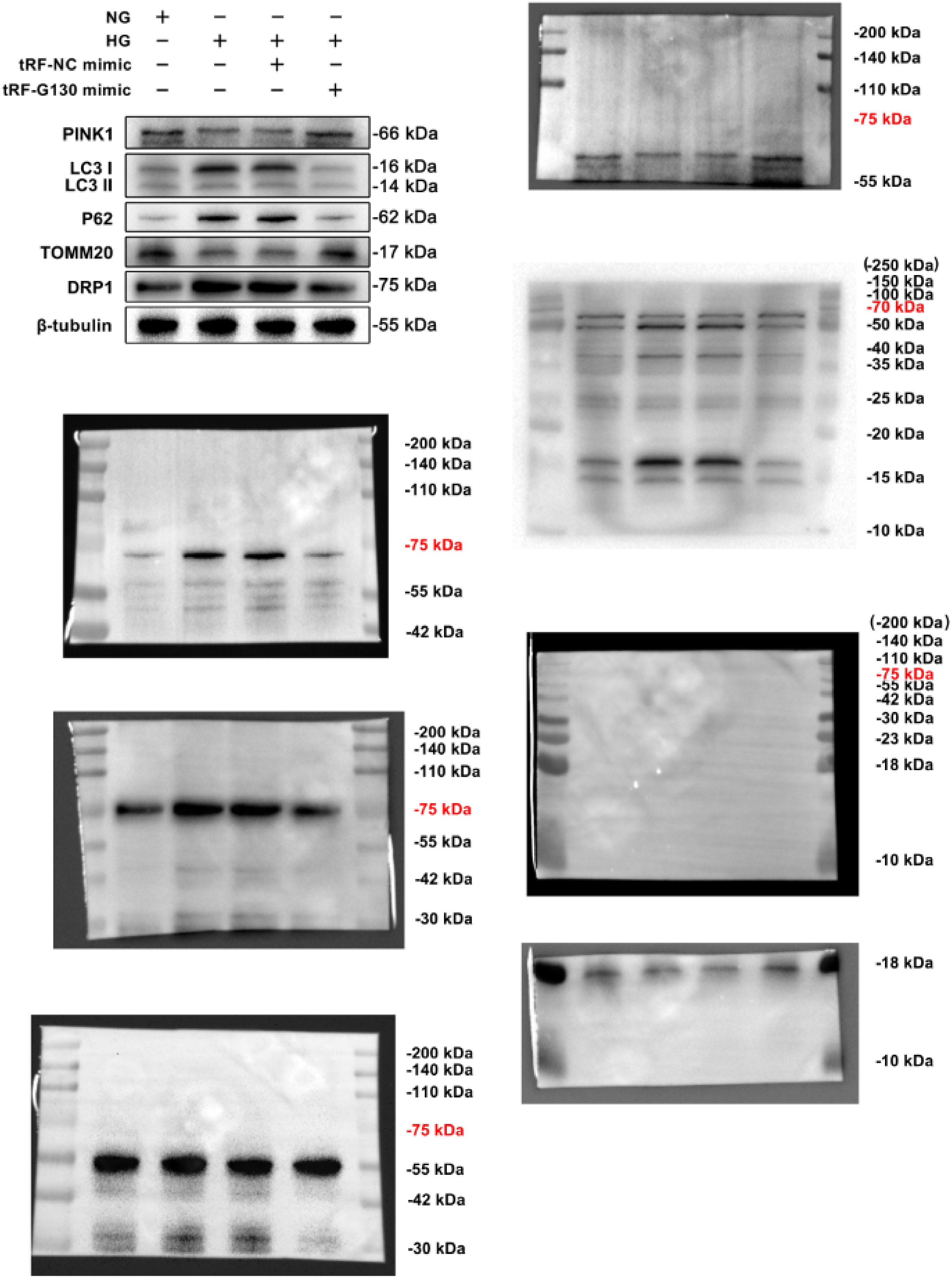

NG + − − −
HG − + + +
tRF-NC mimic − − + −
tRF-G130 mimic − − − +
PINK1 -66 kDa
LC3 I -16 kDa
LC3 II -14 kDa
P62 -62 kDa
TOMM20 -17 kDa
DRP1 -75 kDa
β-tubulin -55 kDa
-200 kDa
-140 kDa
-110 kDa
-75 kDa
-55 kDa
(-250 kDa)
-150 kDa
-100 kDa
-70 kDa
-50 kDa
-40 kDa
-35 kDa
-25 kDa
-20 kDa
-15 kDa
-10 kDa
-200 kDa
-140 kDa
-110 kDa
-75 kDa
-55 kDa
-42 kDa
(-200 kDa)
-140 kDa
-110 kDa
-75 kDa
-55 kDa
-42 kDa
-30 kDa
-23 kDa
-18 kDa
-10 kDa
-200 kDa
-140 kDa
-110 kDa
-75 kDa
-55 kDa
-42 kDa
-30 kDa
-18 kDa
-10 kDa
-200 kDa
-140 kDa
-110 kDa
-75 kDa
-55 kDa
-42 kDa
-30 kDa

Figure. 7F

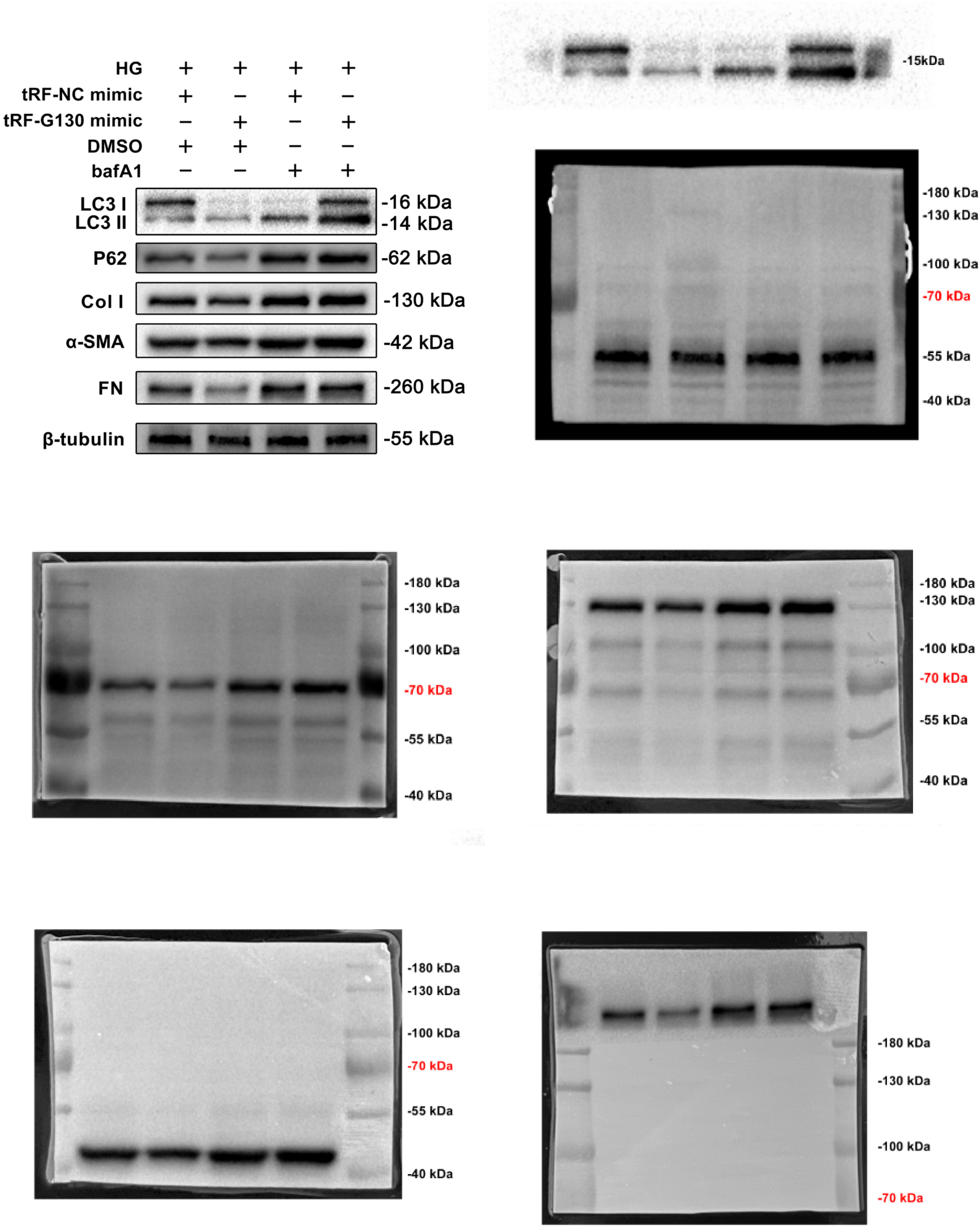
HG + + + +
tRF-NC mimic + − + −
tRF-G130 mimic − + − +
DMSO + + − −
bafA1 − − + +
LC3 I
LC3 II
-16 kDa
-14 kDa
P62
-62 kDa
Col I
-130 kDa
α-SMA
-42 kDa
FN
-260 kDa
β-tubulin
-55 kDa
-15kDa
-180 kDa
-130 kDa
-100 kDa
-70 kDa
-55 kDa
-40 kDa

F1A			
SCR-control1	9.573	UTR-control1	1.209
SCR-control2	16.981	UTR-control2	2.810
SCR-control3	18.707	UTR-control3	1.495
SCR-control4	16.213	UTR-control4	1.316
SCR-control5	16.938	UTR-control5	3.374
SCR-control6	10.218	UTR-control6	1.365
SCR-control7	20.349	UTR-control7	2.776
SCR-DKD1	62.854	UTR-DKD1	24.771
SCR-DKD2	65.978	UTR-DKD2	22.018
SCR-DKD3	57.815	UTR-DKD3	27.851
SCR-DKD4	61.452	UTR-DKD4	27.032
SCR-DKD5	54.800	UTR-DKD5	22.828
SCR-DKD6	64.959	UTR-DKD6	17.282
SCR-DKD7	62.269	UTR-DKD7	18.455

F1B					
masson-control1	6.39	SR-control1	3.68	PAS-control1	0.37
masson-control2	7.63	SR-control2	5.98	PAS-control2	0.56
masson-control3	4.12	SR-control3	1.99	PAS-control3	1.46
masson-control4	5.94	SR-control4	7.42	PAS-control4	0.78
masson-control5	3.33	SR-control5	6.55	PAS-control5	0.96
masson-control6	3.78	SR-control6	2.65	PAS-control6	0.13
masson-control7	4.84	SR-control7	5.12	PAS-control7	0.34
masson-DKD1	55.77	SR-DKD1	62.65	PAS-DKD1	4.51
masson-DKD2	54.33	SR-DKD2	60.08	PAS-DKD2	4.28
masson-DKD3	55.53	SR-DKD3	56.33	PAS-DKD3	3.58
masson-DKD4	44.71	SR-DKD4	59.77	PAS-DKD4	3.14
masson-DKD5	38.70	SR-DKD5	50.14	PAS-DKD5	3.72
masson-DKD6	53.16	SR-DKD6	55.72	PAS-DKD6	4.67
masson-DKD7	42.33	SR-DKD7	61.50	PAS-DKD7	5.10

F1C			
Col I-control1	4.57	FN-control1	2.68
Col I-control2	1.82	FN-control2	4.34
Col I-control3	1.17	FN-control3	1.25
Col I-control4	3.30	FN-control4	2.96
Col I-control5	3.89	FN-control5	4.69
Col I-control6	2.65	FN-control6	1.92
Col I-control7	2.99	FN-control7	3.93
Col I-DKD1	41.88	FN-DKD1	40.89
Col I-DKD2	38.61	FN-DKD2	40.40
Col I-DKD3	34.99	FN-DKD3	44.07
Col I-DKD4	35.36	FN-DKD4	41.46
Col I-DKD5	34.27	FN-DKD5	36.97
Col I-DKD6	30.84	FN-DKD6	50.34
Col I-DKD7	31.01	FN-DKD7	49.67

SF1A					
Col I-control1	1.09	a-SMA-control1	1.06	FN-control1	0.86
Col I-control2	0.81	a-SMA-control2	1.18	FN-control2	1.16
Col I-control3	0.90	a-SMA-control3	0.88	FN-control3	0.96
Col I-control4	0.94	a-SMA-control4	1.19	FN-control4	1.12
Col I-control5	0.95	a-SMA-control5	0.89	FN-control5	0.87
Col I-control6	1.02	a-SMA-control6	1.10	FN-control6	1.06
Col I-control7	1.29	a-SMA-control7	0.70	FN-control7	0.97
Col I-DKD1	1.57	a-SMA-DKD1	1.96	FN-DKD1	1.81
Col I-DKD2	2.11	a-SMA-DKD2	1.92	FN-DKD2	2.09
Col I-DKD3	2.13	a-SMA-DKD3	1.81	FN-DKD3	2.04
Col I-DKD4	1.77	a-SMA-DKD4	1.87	FN-DKD4	1.98
Col I-DKD5	1.84	a-SMA-DKD5	2.03	FN-DKD5	2.10
Col I-DKD6	1.91	a-SMA-DKD6	1.85	FN-DKD6	1.78
Col I-DKD7	1.59	a-SMA-DKD7	1.64	FN-DKD7	1.97

F1D					
Col I-control1	0.92	a-SMA-control1	1.19	FN-control1	1.16
Col I-control2	0.97	a-SMA-control2	0.98	FN-control2	0.82
Col I-control3	0.93	a-SMA-control3	0.84	FN-control3	1.10
Col I-control4	0.95	a-SMA-control4	0.91	FN-control4	1.13
Col I-control5	1.06	a-SMA-control5	1.04	FN-control5	1.18
Col I-control6	1.14	a-SMA-control6	1.18	FN-control6	0.85
Col I-control7	1.03	a-SMA-control7	0.86	FN-control7	0.76
Col I-DKD1	2.08	a-SMA-DKD1	1.74	FN-DKD1	1.88
Col I-DKD2	1.86	a-SMA-DKD2	2.32	FN-DKD2	2.27
Col I-DKD3	1.97	a-SMA-DKD3	1.95	FN-DKD3	1.94
Col I-DKD4	2.30	a-SMA-DKD4	1.92	FN-DKD4	2.11
Col I-DKD5	1.83	a-SMA-DKD5	1.84	FN-DKD5	2.08
Col I-DKD6	2.23	a-SMA-DKD6	2.28	FN-DKD6	2.24
Col I-DKD7	1.94	a-SMA-DKD7	1.90	FN-DKD7	2.39

F1E	
tRF-control1	0.92
tRF-control2	1.03
tRF-control3	1.05
tRF-control4	0.99
tRF-control5	0.98
tRF-control6	0.91
tRF-control7	1.12
tRF-DKD1	0.43
tRF-DKD2	0.57
tRF-DKD3	0.52
tRF-DKD4	0.67
tRF-DKD5	0.35
tRF-DKD6	0.44
tRF-DKD7	0.45

F1H					
Col I-NG1	0.99	a-SMA-NG1	0.92	FN-NG1	0.98
Col I-NG2	1.14	a-SMA-NG2	0.83	FN-NG2	1.20
Col I-NG3	0.87	a-SMA-NG3	1.25	FN-NG3	0.82
Col I-HG1	1.84	a-SMA-HG1	2.03	FN-HG1	2.26
Col I-HG2	1.87	a-SMA-HG2	1.79	FN-HG2	1.70
Col I-HG3	2.10	a-SMA-HG3	1.96	FN-HG3	2.01
Col I-mannitol1	0.88	a-SMA-mannitol1	0.98	FN-mannitol1	0.83
Col I-mannitol2	0.82	a-SMA-mannitol2	1.04	FN-mannitol2	1.01
Col I-mannitol3	1.09	a-SMA-mannitol3	1.16	FN-mannitol3	1.13

SF1B					
Col I-NG1	1.02	a-SMA-NG1	1.11	FN-NG1	0.88
Col I-NG2	1.07	a-SMA-NG2	1.01	FN-NG2	1.15
Col I-NG3	0.91	a-SMA-NG3	0.87	FN-NG3	0.97
Col I-HG1	2.12	a-SMA-HG1	1.93	FN-HG1	2.04
Col I-HG2	1.81	a-SMA-HG2	2.06	FN-HG2	2.30
Col I-HG3	2.26	a-SMA-HG3	1.91	FN-HG3	1.87
Col I-mannitol1	0.94	a-SMA-mannitol1	1.06	FN-mannitol1	0.94
Col I-mannitol2	0.89	a-SMA-mannitol2	0.90	FN-mannitol2	0.87
Col I-mannitol3	1.05	a-SMA-mannitol3	1.16	FN-mannitol3	1.03

F1I	
tRF-NG1	0.92
tRF-NG2	0.97
tRF-NG3	1.11
tRF-HG1	0.37
tRF-HG2	0.58
tRF-HG3	0.46
tRF-mannitol1	1.02
tRF-mannitol2	0.97
tRF-mannitol3	0.96

F2A		SF1D				F2B-SCR		F2B-UTR	
tRF-control1	1.05	AAV-NC-kidney1	0.89	AAV-NC-heart1	0.93	control1	11.155	control1	2.257
tRF-control2	1.01	AAV-NC-kidney2	1.10	AAV-NC-heart2	1.13	control2	14.042	control2	2.218
tRF-control3	1.08	AAV-NC-kidney3	0.81	AAV-NC-heart3	1.09	control3	21.944	control3	2.735
tRF-control4	0.94	AAV-NC-kidney4	1.18	AAV-NC-heart4	1.08	control4	17.780	control4	4.025
tRF-control5	1.01	AAV-NC-kidney5	0.94	AAV-NC-heart5	0.96	control5	15.125	control5	3.647
tRF-control6	0.89	AAV-NC-kidney6	1.03	AAV-NC-heart6	1.12	control6	10.077	control6	2.536
tRF-control7	1.02	AAV-NC-kidney7	1.05	AAV-NC-heart7	0.69	control7	15.611	control7	1.595
tRF-DKD1	0.41	AAV-tRF-kidney1	17.31	AAV-tRF-heart1	14.83	DKD1	56.121	DKD1	23.271
tRF-DKD2	0.56	AAV-tRF-kidney2	12.93	AAV-tRF-heart2	14.19	DKD2	69.233	DKD2	24.179
tRF-DKD3	0.54	AAV-tRF-kidney3	17.87	AAV-tRF-heart3	15.50	DKD3	62.258	DKD3	17.880
tRF-DKD4	0.44	AAV-tRF-kidney4	19.45	AAV-tRF-heart4	13.87	DKD4	62.110	DKD4	27.285
tRF-DKD5	0.58	AAV-tRF-kidney5	12.13	AAV-tRF-heart5	13.19	DKD5	69.430	DKD5	30.284
tRF-DKD6	0.49	AAV-tRF-kidney6	14.32	AAV-tRF-heart6	15.07	DKD6	72.382	DKD6	23.793
tRF-DKD7	0.57	AAV-tRF-kidney7	19.89	AAV-tRF-heart7	15.70	DKD7	79.866	DKD7	18.510
tRF-NC1	0.60	AAV-NC-brain1	1.13	AAV-NC-liver1	0.96	tRF-NC1	76.809	tRF-NC1	21.132
tRF-NC2	0.38	AAV-NC-brain2	0.96	AAV-NC-liver2	1.16	tRF-NC2	63.141	tRF-NC2	20.076
tRF-NC3	0.50	AAV-NC-brain3	0.85	AAV-NC-liver3	0.90	tRF-NC3	71.718	tRF-NC3	17.797
tRF-NC4	0.59	AAV-NC-brain4	0.94	AAV-NC-liver4	0.86	tRF-NC4	58.358	tRF-NC4	22.279
tRF-NC5	0.43	AAV-NC-brain5	0.91	AAV-NC-liver5	0.92	tRF-NC5	77.132	tRF-NC5	31.975
tRF-NC6	0.58	AAV-NC-brain6	1.17	AAV-NC-liver6	1.02	tRF-NC6	77.887	tRF-NC6	23.524
tRF-NC7	0.46	AAV-NC-brain7	1.04	AAV-NC-liver7	1.18	tRF-NC7	74.237	tRF-NC7	25.660
AAV-tRF1	5.43	AAV-tRF-brain1	6.47	AAV-tRF-liver1	21.66	AAV-tRF1	39.644	AAV-tRF1	12.091
AAV-tRF2	4.88	AAV-tRF-brain2	4.58	AAV-tRF-liver2	29.25	AAV-tRF2	41.015	AAV-tRF2	13.828
AAV-tRF3	4.18	AAV-tRF-brain3	3.58	AAV-tRF-liver3	24.43	AAV-tRF3	40.469	AAV-tRF3	8.636
AAV-tRF4	3.51	AAV-tRF-brain4	6.66	AAV-tRF-liver4	18.71	AAV-tRF4	54.643	AAV-tRF4	12.863
AAV-tRF5	4.79	AAV-tRF-brain5	5.64	AAV-tRF-liver5	28.26	AAV-tRF5	53.298	AAV-tRF5	11.200
AAV-tRF6	5.49	AAV-tRF-brain6	4.86	AAV-tRF-liver6	22.92	AAV-tRF6	49.487	AAV-tRF6	10.181
AAV-tRF7	4.13	AAV-tRF-brain7	4.48	AAV-tRF-liver7	29.59	AAV-tRF7	46.075	AAV-tRF7	14.352

F2Cmasson		F2CSR		F2CPAS		SF1E-Col I		SF1E-a-SMA		SF1E-FN	
control1	6.86	control1	2.31	control1	0.98	control1	0.83	control1	0.98	control1	0.89
control2	5.32	control2	8.81	control2	1.15	control2	1.13	control2	1.02	control2	1.08
control3	2.99	control3	6.98	control3	0.38	control3	0.82	control3	0.84	control3	0.88
control4	2.54	control4	3.43	control4	1.17	control4	1.20	control4	0.94	control4	0.99
control5	5.28	control5	6.83	control5	0.51	control5	0.94	control5	0.87	control5	1.17
control6	7.05	control6	2.58	control6	1.04	control6	0.82	control6	1.08	control6	0.81
control7	4.89	control7	3.65	control7	0.57	control7	1.26	control7	1.27	control7	1.18
DKD1	54.31	DKD1	40.10	DKD1	4.22	DKD1	2.11	DKD1	2.24	DKD1	1.59
DKD2	47.30	DKD2	51.43	DKD2	4.26	DKD2	2.23	DKD2	1.70	DKD2	1.54
DKD3	57.48	DKD3	58.48	DKD3	3.16	DKD3	2.17	DKD3	1.63	DKD3	1.86
DKD4	48.34	DKD4	50.33	DKD4	3.48	DKD4	1.50	DKD4	2.17	DKD4	1.57
DKD5	50.44	DKD5	40.74	DKD5	3.99	DKD5	1.89	DKD5	2.04	DKD5	1.98
DKD6	42.08	DKD6	44.37	DKD6	4.63	DKD6	2.14	DKD6	2.22	DKD6	2.23
DKD7	41.30	DKD7	50.19	DKD7	3.76	DKD7	1.96	DKD7	1.87	DKD7	2.05
tRF-NC1	42.97	tRF-NC1	42.11	tRF-NC1	3.54	tRF-NC1	1.95	tRF-NC1	1.99	tRF-NC1	1.88
tRF-NC2	50.60	tRF-NC2	59.45	tRF-NC2	3.11	tRF-NC2	1.75	tRF-NC2	1.91	tRF-NC2	1.52
tRF-NC3	49.87	tRF-NC3	50.66	tRF-NC3	4.52	tRF-NC3	1.84	tRF-NC3	1.95	tRF-NC3	2.15
tRF-NC4	47.64	tRF-NC4	40.53	tRF-NC4	3.18	tRF-NC4	1.96	tRF-NC4	1.99	tRF-NC4	2.17
tRF-NC5	42.86	tRF-NC5	51.21	tRF-NC5	4.54	tRF-NC5	1.82	tRF-NC5	2.06	tRF-NC5	1.71
tRF-NC6	40.31	tRF-NC6	55.60	tRF-NC6	4.95	tRF-NC6	1.79	tRF-NC6	1.58	tRF-NC6	1.98
tRF-NC7	57.36	tRF-NC7	56.21	tRF-NC7	4.24	tRF-NC7	2.24	tRF-NC7	1.79	tRF-NC7	2.02
AAV-tRF1	23.78	AAV-tRF1	19.27	AAV-tRF1	2.25	AAV-tRF1	1.17	AAV-tRF1	1.43	AAV-tRF1	1.25
AAV-tRF2	18.22	AAV-tRF2	21.02	AAV-tRF2	1.50	AAV-tRF2	1.19	AAV-tRF2	1.29	AAV-tRF2	1.38
AAV-tRF3	17.91	AAV-tRF3	17.14	AAV-tRF3	2.02	AAV-tRF3	1.21	AAV-tRF3	1.30	AAV-tRF3	1.27
AAV-tRF4	19.34	AAV-tRF4	18.43	AAV-tRF4	2.26	AAV-tRF4	1.27	AAV-tRF4	1.21	AAV-tRF4	1.29
AAV-tRF5	17.77	AAV-tRF5	15.05	AAV-tRF5	1.79	AAV-tRF5	1.31	AAV-tRF5	1.12	AAV-tRF5	1.18
AAV-tRF6	21.83	AAV-tRF6	15.35	AAV-tRF6	2.16	AAV-tRF6	1.37	AAV-tRF6	1.29	AAV-tRF6	1.27
AAV-tRF7	21.10	AAV-tRF7	12.65	AAV-tRF7	2.31	AAV-tRF7	1.28	AAV-tRF7	1.13	AAV-tRF7	1.22

F2D-Col I		F2D-a-SMA		F2D-FN		F2E-Col I		F2E-FN	
control1	1.02	control1	1.08	control1	1.13	control1	0.68	control1	0.82
control2	1.13	control2	0.80	control2	1.08	control2	0.15	control2	0.69
control3	1.19	control3	1.20	control3	0.85	control3	0.87	control3	0.39
control4	0.84	control4	0.91	control4	1.06	control4	0.62	control4	0.99
control5	1.09	control5	0.96	control5	0.95	control5	0.90	control5	0.10
control6	0.83	control6	0.89	control6	0.91	control6	0.83	control6	0.02
control7	0.90	control7	1.16	control7	1.02	control7	0.27	control7	0.65
DKD1	2.37	DKD1	1.78	DKD1	2.29	DKD1	24.01	DKD1	34.14
DKD2	2.17	DKD2	2.20	DKD2	1.92	DKD2	31.02	DKD2	35.97
DKD3	1.85	DKD3	2.12	DKD3	1.71	DKD3	25.85	DKD3	25.59
DKD4	1.67	DKD4	2.06	DKD4	2.15	DKD4	23.53	DKD4	35.60
DKD5	1.54	DKD5	2.39	DKD5	1.81	DKD5	31.34	DKD5	24.78
DKD6	1.50	DKD6	1.90	DKD6	2.33	DKD6	23.25	DKD6	29.51
DKD7	1.81	DKD7	2.24	DKD7	2.28	DKD7	24.05	DKD7	27.44
tRF-NC1	1.58	tRF-NC1	1.83	tRF-NC1	2.02	tRF-NC1	28.97	tRF-NC1	29.72
tRF-NC2	2.14	tRF-NC2	2.10	tRF-NC2	2.23	tRF-NC2	24.07	tRF-NC2	21.44
tRF-NC3	1.56	tRF-NC3	2.06	tRF-NC3	1.79	tRF-NC3	30.88	tRF-NC3	24.48
tRF-NC4	1.51	tRF-NC4	2.15	tRF-NC4	1.87	tRF-NC4	20.85	tRF-NC4	29.17
tRF-NC5	2.13	tRF-NC5	1.88	tRF-NC5	1.92	tRF-NC5	24.49	tRF-NC5	32.49
tRF-NC6	2.04	tRF-NC6	2.09	tRF-NC6	2.11	tRF-NC6	27.23	tRF-NC6	30.02
tRF-NC7	1.75	tRF-NC7	2.07	tRF-NC7	1.70	tRF-NC7	32.21	tRF-NC7	35.47
AAV-tRF1	1.34	AAV-tRF1	1.22	AAV-tRF1	1.01	AAV-tRF1	8.46	AAV-tRF1	12.23
AAV-tRF2	1.68	AAV-tRF2	1.34	AAV-tRF2	1.22	AAV-tRF2	12.02	AAV-tRF2	14.84
AAV-tRF3	1.71	AAV-tRF3	1.28	AAV-tRF3	1.05	AAV-tRF3	13.33	AAV-tRF3	10.52
AAV-tRF4	1.66	AAV-tRF4	1.44	AAV-tRF4	1.13	AAV-tRF4	14.56	AAV-tRF4	14.12
AAV-tRF5	1.44	AAV-tRF5	1.23	AAV-tRF5	1.25	AAV-tRF5	13.13	AAV-tRF5	9.80
AAV-tRF6	1.40	AAV-tRF6	1.41	AAV-tRF6	1.17	AAV-tRF6	12.93	AAV-tRF6	13.41
AAV-tRF7	1.31	AAV-tRF7	1.30	AAV-tRF7	1.04	AAV-tRF7	9.23	AAV-tRF7	11.75

F2F-tRF	
NG1	0.95
NG2	1.21
NG3	0.84
HG1	0.24
HG2	0.31
HG3	0.39
tRF-NC1	0.42
tRF-NC2	0.23
tRF-NC3	0.34
tRF-mimic1	669.59
tRF-mimic2	631.52
tRF-mimic3	712.96

SF1F-Col I		SF1F-a-SMA		SF1F-FN	
NG1	0.92	NG1	0.84	NG1	1.14
NG2	0.84	NG2	1.19	NG2	0.85
NG3	1.04	NG3	0.97	NG3	1.01
HG1	2.38	HG1	2.29	HG1	1.70
HG2	1.91	HG2	2.01	HG2	1.56
HG3	2.02	HG3	1.90	HG3	1.83
tRF-NC1	2.28	tRF-NC1	2.30	tRF-NC1	1.85
tRF-NC2	1.88	tRF-NC2	1.85	tRF-NC2	2.14
tRF-NC3	2.05	tRF-NC3	2.06	tRF-NC3	1.71
tRF-mimic1	1.47	tRF-mimic1	1.79	tRF-mimic1	1.30
tRF-mimic2	1.75	tRF-mimic2	1.47	tRF-mimic2	1.35
tRF-mimic3	1.55	tRF-mimic3	1.31	tRF-mimic3	1.22

F2G-Col I		F2G-a-SMA		F2G-FN	
NG1	0.87	NG1	1.13	NG1	1.15
NG2	0.99	NG2	0.97	NG2	0.90
NG3	1.14	NG3	0.90	NG3	0.95
HG1	1.87	HG1	1.87	HG1	1.67
HG2	2.12	HG2	1.96	HG2	1.94
HG3	1.82	HG3	1.74	HG3	1.77
tRF-NC1	2.05	tRF-NC1	2.03	tRF-NC1	1.79
tRF-NC2	1.71	tRF-NC2	1.94	tRF-NC2	1.96
tRF-NC3	1.76	tRF-NC3	2.01	tRF-NC3	2.08
tRF-mimic1	1.41	tRF-mimic1	1.35	tRF-mimic1	1.03
tRF-mimic2	1.51	tRF-mimic2	1.31	tRF-mimic2	0.94
tRF-mimic3	1.63	tRF-mimic3	1.40	tRF-mimic3	1.10

F3B	
IgG1	1.148
IgG2	0.949
IgG3	0.903
FBXO7-1	8.830
FBXO7-2	6.774
FBXO7-3	6.497

F3C-FBXO7	
control1	1.22
control2	0.90
control3	0.88
control4	0.96
control5	1.22
control6	1.16
control7	0.66
DKD1	2.79
DKD2	2.90
DKD3	2.92
DKD4	2.13
DKD5	1.97
DKD6	2.48
DKD7	2.92
tRF-NC1	2.69
tRF-NC2	2.76
tRF-NC3	2.88
tRF-NC4	2.56
tRF-NC5	2.62
tRF-NC6	2.39
tRF-NC7	2.55
AAV-tRF1	2.60
AAV-tRF2	2.92
AAV-tRF3	2.88
AAV-tRF4	2.48
AAV-tRF5	2.36
AAV-tRF6	2.10
AAV-tRF7	2.63

F3D-FBXO7	
NG1	1.12
NG2	0.89
NG3	0.99
HG1	2.41
HG2	1.88
HG3	1.92
tRF-NC1	2.13
tRF-NC2	1.76
tRF-NC3	2.38
tRF-mimic1	1.88
tRF-mimic2	2.23
tRF-mimic3	1.83

F3F-FBXO7	
NG1	0.98
NG2	0.91
NG3	1.11
HG1	2.23
HG2	1.94
HG3	1.70
tRF-NC1	2.12
tRF-NC2	1.68
tRF-NC3	2.22
tRF-mimic1	1.24
tRF-mimic2	1.12
tRF-mimic3	0.81

F3E-FBXO7	
control1	1.13
control2	0.93
control3	0.82
control4	0.89
control5	1.10
control6	1.02
control7	1.11
DKD1	2.63
DKD2	2.54
DKD3	2.77
DKD4	2.19
DKD5	2.52
DKD6	2.39
DKD7	2.51
tRF-NC1	2.46
tRF-NC2	2.53
tRF-NC3	2.04
tRF-NC4	2.97
tRF-NC5	2.02
tRF-NC6	1.99
tRF-NC7	2.63
AAV-tRF1	1.04
AAV-tRF2	1.46
AAV-tRF3	1.10
AAV-tRF4	1.37
AAV-tRF5	1.42
AAV-tRF6	1.24
AAV-tRF7	1.39

F3G-FBXO7	
NC-mimic-0-1	1.09
NC-mimic-0-2	0.95
NC-mimic-0-3	0.96
NC-mimic-2-1	1.11
NC-mimic-2-2	0.98
NC-mimic-2-3	0.91
NC-mimic-4-1	0.85
NC-mimic-4-2	0.79
NC-mimic-4-3	0.87
NC-mimic-6-1	0.61
NC-mimic-6-2	0.64
NC-mimic-6-3	0.58
tRF mimic-0-1	0.65
tRF mimic-0-2	0.73
tRF mimic-0-3	0.68
tRF mimic-2-1	0.35
tRF mimic-2-2	0.38
tRF mimic-2-3	0.42
tRF mimic-4-1	0.49
tRF mimic-4-2	0.51
tRF mimic-4-3	0.41
tRF mimic-6-1	0.24
tRF mimic-6-2	0.18
tRF mimic-6-3	0.31

F3H-FBXO7	
MG132-0-1	0.82
MG132-0-2	0.93
MG132-0-3	1.25
MG132-10-1	1.65
MG132-10-2	1.49
MG132-10-3	1.71
MG132-25-1	2.83
MG132-25-2	2.27
MG132-25-3	2.38

F3I-FBXO7	
HG1	0.88
HG2	1.02
HG3	1.10
tRF mimic1	0.37
tRF mimic2	0.66
tRF mimic3	0.51
MG132-1	0.87
MG132-2	0.98
MG132-3	1.04
BafA1-1	0.48
BafA1-2	0.57
BafA1-3	0.34

F4A-FBXO7	
control1	0.87
control2	1.14
control3	0.96
control4	1.09
control5	1.07
control6	1.05
control7	0.82
DKD1	2.63
DKD2	2.41
DKD3	2.79
DKD4	2.34
DKD5	2.18
DKD6	1.97
DKD7	1.84
AAV-NC1	2.40
AAV-NC2	2.56
AAV-NC3	2.43
AAV-NC4	1.98
AAV-NC5	2.15
AAV-NC6	2.01
AAV-NC7	2.10
FBXO7RNAi1	1.14
FBXO7RNAi2	0.95
FBXO7RNAi3	0.69
FBXO7RNAi4	1.23
FBXO7RNAi5	1.21
FBXO7RNAi6	1.44
FBXO7RNAi7	1.16

F4B-FBXO7	
control1	1.12
control2	1.14
control3	1.04
control4	0.89
control5	1.15
control6	0.94
control7	0.72
DKD1	2.54
DKD2	2.47
DKD3	2.50
DKD4	2.38
DKD5	1.96
DKD6	2.11
DKD7	1.90
AAV-NC1	2.69
AAV-NC2	2.66
AAV-NC3	2.73
AAV-NC4	1.84
AAV-NC5	1.93
AAV-NC6	2.29
AAV-NC7	2.11
FBXO7RNAi1	0.76
FBXO7RNAi2	0.83
FBXO7RNAi3	0.66
FBXO7RNAi4	0.74
FBXO7RNAi5	0.93
FBXO7RNAi6	0.41
FBXO7RNAi7	0.54

F4C-SCR-AAV-N	79.588
AAV-NC2	68.377
AAV-NC3	69.055
AAV-NC4	60.676
AAV-NC5	61.281
AAV-NC6	59.980
AAV-NC7	59.051
FBXO7RNAi1	20.705
FBXO7RNAi2	38.729
FBXO7RNAi3	37.281
FBXO7RNAi4	19.364
FBXO7RNAi5	22.085
FBXO7RNAi6	21.920
FBXO7RNAi7	19.015

F4C-UTR-AAV-N	16.502
AAV-NC2	19.453
AAV-NC3	20.102
AAV-NC4	19.890
AAV-NC5	29.065
AAV-NC6	26.569
AAV-NC7	23.907
FBXO7RNAi1	3.582
FBXO7RNAi2	2.492
FBXO7RNAi3	2.155
FBXO7RNAi4	1.905
FBXO7RNAi5	1.911
FBXO7RNAi6	2.170
FBXO7RNAi7	2.138

F4D-IHC	
control1	8.02
control2	8.85
control3	12.31
control4	8.45
control5	6.90
control6	11.73
control7	7.96
DKD1	75.50
DKD2	78.11
DKD3	73.38
DKD4	72.28
DKD5	71.07
DKD6	72.25
DKD7	70.92
AAV-NC1	79.48
AAV-NC2	81.17
AAV-NC3	80.92
AAV-NC4	77.82
AAV-NC5	73.23
AAV-NC6	71.18
AAV-NC7	77.09
FBXO7RNAi1	13.77
FBXO7RNAi2	10.91
FBXO7RNAi3	11.15
FBXO7RNAi4	12.36
FBXO7RNAi5	12.64
FBXO7RNAi6	11.00
FBXO7RNAi7	14.08

F4E-MAS-AAV-N	64.50
AAV-NC2	60.24
AAV-NC3	58.31
AAV-NC4	55.36
AAV-NC5	58.17
AAV-NC6	54.83
AAV-NC7	60.60
FBXO7RNAi1	27.90
FBXO7RNAi2	23.07
FBXO7RNAi3	26.71
FBXO7RNAi4	30.21
FBXO7RNAi5	22.87
FBXO7RNAi6	21.50
FBXO7RNAi7	20.17

F4E-SR-AAV-NC	56.30
AAV-NC2	55.26
AAV-NC3	59.20
AAV-NC4	50.28
AAV-NC5	44.04
AAV-NC6	49.55
AAV-NC7	58.73
FBXO7RNAi1	23.06
FBXO7RNAi2	28.57
FBXO7RNAi3	27.21
FBXO7RNAi4	30.89
FBXO7RNAi5	32.77
FBXO7RNAi6	33.53
FBXO7RNAi7	28.37

F4E-PAS-AAV-N	4.33
AAV-NC2	4.62
AAV-NC3	4.03
AAV-NC4	3.99
AAV-NC5	3.79
AAV-NC6	4.23
AAV-NC7	4.08
FBXO7RNAi1	2.02
FBXO7RNAi2	2.01
FBXO7RNAi3	1.90
FBXO7RNAi4	1.88
FBXO7RNAi5	1.89
FBXO7RNAi6	1.68
FBXO7RNAi7	2.03

F4F-Col I-NC1	22.10
AAV-NC2	26.26
AAV-NC3	30.02
AAV-NC4	25.47
AAV-NC5	34.11
AAV-NC6	26.08
AAV-NC7	25.16
FBXO7RNAi1	10.66
FBXO7RNAi2	7.69
FBXO7RNAi3	9.52
FBXO7RNAi4	9.29
FBXO7RNAi5	8.04
FBXO7RNAi6	14.24
FBXO7RNAi7	8.55

F4F-FN-NC1	30.49
AAV-NC2	31.43
AAV-NC3	22.88
AAV-NC4	33.60
AAV-NC5	31.56
AAV-NC6	28.05
AAV-NC7	25.54
FBXO7RNAi1	7.77
FBXO7RNAi2	7.38
FBXO7RNAi3	6.35
FBXO7RNAi4	8.21
FBXO7RNAi5	9.34
FBXO7RNAi6	9.51
FBXO7RNAi7	8.20

SF2D-Col I	
control1	1.11
control2	1.00
control3	1.18
control4	0.80
control5	0.93
control6	0.85
control7	1.13
DKD1	2.20
DKD2	2.38
DKD3	1.58
DKD4	2.36
DKD5	1.76
DKD6	1.60
DKD7	1.77
AAV-NC1	1.98
AAV-NC2	1.69
AAV-NC3	1.70
AAV-NC4	2.05
AAV-NC5	2.18
AAV-NC6	2.24
AAV-NC7	2.15
FBXO7RNAi1	1.12
FBXO7RNAi2	1.42
FBXO7RNAi3	1.28
FBXO7RNAi4	1.08
FBXO7RNAi5	1.62
FBXO7RNAi6	1.50
FBXO7RNAi7	1.03

SF2D-a-SMA	
control1	0.84
control2	1.11
control3	1.08
control4	0.87
control5	1.09
control6	0.89
control7	1.12
DKD1	1.85
DKD2	2.38
DKD3	2.32
DKD4	1.92
DKD5	2.39
DKD6	1.68
DKD7	2.44
AAV-NC1	1.92
AAV-NC2	2.40
AAV-NC3	1.80
AAV-NC4	1.56
AAV-NC5	1.85
AAV-NC6	1.69
AAV-NC7	2.27
FBXO7RNAi1	1.41
FBXO7RNAi2	1.61
FBXO7RNAi3	1.19
FBXO7RNAi4	0.72
FBXO7RNAi5	1.23
FBXO7RNAi6	0.91
FBXO7RNAi7	0.81

SF2D-FN	
control1	0.95
control2	1.11
control3	0.93
control4	0.89
control5	1.02
control6	1.07
control7	1.03
DKD1	1.98
DKD2	2.08
DKD3	1.82
DKD4	1.53
DKD5	2.04
DKD6	2.21
DKD7	2.43
AAV-NC1	1.93
AAV-NC2	2.16
AAV-NC3	1.98
AAV-NC4	1.76
AAV-NC5	1.85
AAV-NC6	1.68
AAV-NC7	2.22
FBXO7RNAi1	1.30
FBXO7RNAi2	1.22
FBXO7RNAi3	0.90
FBXO7RNAi4	0.84
FBXO7RNAi5	1.78
FBXO7RNAi6	1.23
FBXO7RNAi7	1.35

F4G-Col I	
control1	1.01
control2	0.99
control3	1.12
control4	0.83
control5	1.01
control6	1.34
control7	0.70
DKD1	1.86
DKD2	1.98
DKD3	1.99
DKD4	1.89
DKD5	1.51
DKD6	1.86
DKD7	1.52
AAV-NC1	1.51
AAV-NC2	2.39
AAV-NC3	2.26
AAV-NC4	1.59
AAV-NC5	2.43
AAV-NC6	1.69
AAV-NC7	2.42
FBXO7RNAi1	1.01
FBXO7RNAi2	0.93
FBXO7RNAi3	0.89
FBXO7RNAi4	1.09
FBXO7RNAi5	1.13
FBXO7RNAi6	1.10
FBXO7RNAi7	1.30

F4G-a-SMA	
control1	0.89
control2	0.89
control3	1.02
control4	1.10
control5	1.24
control6	0.99
control7	0.87
DKD1	2.25
DKD2	1.73
DKD3	1.96
DKD4	1.57
DKD5	2.10
DKD6	2.22
DKD7	1.91
AAV-NC1	1.95
AAV-NC2	2.22
AAV-NC3	1.99
AAV-NC4	1.92
AAV-NC5	1.79
AAV-NC6	1.83
AAV-NC7	2.15
FBXO7RNAi1	1.23
FBXO7RNAi2	1.01
FBXO7RNAi3	0.97
FBXO7RNAi4	1.29
FBXO7RNAi5	0.96
FBXO7RNAi6	0.91
FBXO7RNAi7	1.12

F4G-FN	
control1	1.17
control2	0.74
control3	0.97
control4	0.94
control5	1.13
control6	1.03
control7	1.02
DKD1	1.86
DKD2	2.05
DKD3	1.61
DKD4	2.20
DKD5	1.59
DKD6	1.90
DKD7	2.08
AAV-NC1	1.65
AAV-NC2	2.04
AAV-NC3	2.11
AAV-NC4	1.65
AAV-NC5	1.77
AAV-NC6	2.36
AAV-NC7	2.10
FBXO7RNAi1	1.26
FBXO7RNAi2	1.00
FBXO7RNAi3	0.98
FBXO7RNAi4	1.27
FBXO7RNAi5	1.01
FBXO7RNAi6	1.23
FBXO7RNAi7	1.36

SF2E-FBXO7		SF2E-Col I		SF2E-a-SMA		SF2E-FN	
NG1	0.85	NG1	0.99	NG1	0.86	NG1	1.06
NG2	0.99	NG2	1.13	NG2	1.16	NG2	0.89
NG3	1.16	NG3	0.88	NG3	0.98	NG3	1.05
HG1	1.84	HG1	2.18	HG1	1.76	HG1	1.93
HG2	1.99	HG2	2.02	HG2	1.88	HG2	1.70
HG3	1.78	HG3	1.68	HG3	2.01	HG3	2.08
HG+NC1	1.56	HG+NC1	2.06	HG+NC1	2.04	HG+NC1	1.63
HG+NC2	2.02	HG+NC2	1.58	HG+NC2	1.98	HG+NC2	1.53
HG+NC3	1.69	HG+NC3	1.98	HG+NC3	2.18	HG+NC3	2.13
HG+siRNA1	1.37	HG+siRNA1	1.26	HG+siRNA1	1.13	HG+siRNA1	1.32
HG+siRNA2	1.21	HG+siRNA2	1.03	HG+siRNA2	1.16	HG+siRNA2	1.05
HG+siRNA3	1.28	HG+siRNA3	1.32	HG+siRNA3	1.25	HG+siRNA3	1.21

SF2F-FBXO7		SF2F-Col I		SF2F-a-SMA		SF2F-FN	
NG1	1.03	NG1	1.10	NG1	1.18	NG1	0.92
NG2	0.84	NG2	0.81	NG2	0.96	NG2	0.96
NG3	1.13	NG3	1.09	NG3	0.86	NG3	1.12
HG1	1.92	HG1	1.66	HG1	1.71	HG1	1.98
HG2	1.94	HG2	2.21	HG2	1.95	HG2	1.95
HG3	1.87	HG3	2.32	HG3	1.82	HG3	2.04
HG+NC1	1.74	HG+NC1	1.80	HG+NC1	1.96	HG+NC1	1.70
HG+NC2	1.65	HG+NC2	2.32	HG+NC2	1.90	HG+NC2	1.75
HG+NC3	1.96	HG+NC3	2.21	HG+NC3	2.00	HG+NC3	2.07
HG+siRNA1	0.22	HG+siRNA1	1.14	HG+siRNA1	1.02	HG+siRNA1	1.28
HG+siRNA2	0.33	HG+siRNA2	1.27	HG+siRNA2	1.19	HG+siRNA2	1.37
HG+siRNA3	0.35	HG+siRNA3	1.32	HG+siRNA3	1.21	HG+siRNA3	1.19

F4H-FBXO7		F4H-Col I		F4H-a-SMA		F4H-FN	
HG+NC1	0.97	HG+NC1	0.87	HG+NC1	1.13	HG+NC1	0.94
HG+NC2	1.04	HG+NC2	0.89	HG+NC2	1.08	HG+NC2	0.89
HG+NC3	0.99	HG+NC3	1.24	HG+NC3	0.79	HG+NC3	1.17
HG+tRFmimic1	0.32	HG+tRFmimic1	0.41	HG+tRFmimic1	0.51	HG+tRFmimic1	0.46
HG+tRFmimic2	0.40	HG+tRFmimic2	0.52	HG+tRFmimic2	0.59	HG+tRFmimic2	0.41
HG+tRFmimic3	0.39	HG+tRFmimic3	0.43	HG+tRFmimic3	0.55	HG+tRFmimic3	0.58
HG+FBXO71	1.62	HG+FBXO71	1.70	HG+FBXO71	1.69	HG+FBXO71	1.62
HG+FBXO72	1.45	HG+FBXO72	1.61	HG+FBXO72	1.61	HG+FBXO72	1.47
HG+FBXO73	1.58	HG+FBXO73	1.63	HG+FBXO73	1.57	HG+FBXO73	1.83
HG+tRF+FBXO71	1.46	HG+tRF+FBXO71	1.58	HG+tRF+FBXO71	1.51	HG+tRF+FBXO71	1.50
HG+tRF+FBXO72	1.44	HG+tRF+FBXO72	1.62	HG+tRF+FBXO72	1.61	HG+tRF+FBXO72	1.72
HG+tRF+FBXO73	1.61	HG+tRF+FBXO73	1.60	HG+tRF+FBXO73	1.79	HG+tRF+FBXO73	1.54

F4I-FBXO7		F4I-Col I		F4I-a-SMA		F4I-FN	
tRFmimic1	0.81	tRFmimic1	0.87	tRFmimic1	0.87	tRFmimic1	1.07
tRFmimic2	1.14	tRFmimic2	1.17	tRFmimic2	1.06	tRFmimic2	0.99
tRFmimic3	1.05	tRFmimic3	0.96	tRFmimic3	1.07	tRFmimic3	0.94
FBXO7siRNA1	1.05	FBXO7siRNA1	1.15	FBXO7siRNA1	0.85	FBXO7siRNA1	1.07
FBXO7siRNA2	0.92	FBXO7siRNA2	0.96	FBXO7siRNA2	0.88	FBXO7siRNA2	1.18
FBXO7siRNA3	1.17	FBXO7siRNA3	1.17	FBXO7siRNA3	1.01	FBXO7siRNA3	0.95
tRF mimic+FBXO7si1	0.97	tRF mimic+FBXO7si1	0.94	tRF mimic+FBXO7si1	0.83	tRF mimic+FBXO7si1	1.10
tRF mimic+FBXO7si2	0.84	tRF mimic+FBXO7si2	1.12	tRF mimic+FBXO7si2	0.84	tRF mimic+FBXO7si2	1.04
tRF mimic+FBXO7si3	1.02	tRF mimic+FBXO7si3	1.09	tRF mimic+FBXO7si3	1.17	tRF mimic+FBXO7si3	0.91

F5F-PINK1		SF3A-Col I		SF3A-a-SMA		SF3A-FN	
OE-NC1	1.01	FBXO7+NC-1	1.15	FBXO7+NC-1	1.02	FBXO7+NC-1	0.86
OE-NC2	0.97	FBXO7+NC-2	0.99	FBXO7+NC-2	0.91	FBXO7+NC-2	1.15
OE-NC3	1.02	FBXO7+NC-3	0.86	FBXO7+NC-3	1.07	FBXO7+NC-3	0.99
OE-FBXO7-1	1.18	FBXO7+PINK1-1	0.53	FBXO7+PINK1-1	0.30	FBXO7+PINK1-1	0.43
OE-FBXO7-2	1.02	FBXO7+PINK1-2	0.44	FBXO7+PINK1-2	0.64	FBXO7+PINK1-2	0.52
OE-FBXO7-3	0.81	FBXO7+PINK1-3	0.63	FBXO7+PINK1-3	0.41	FBXO7+PINK1-3	0.33
F5G-PINK1		SF3A-Col I		SF3A-a-SMA		SF3A-FN	
OE-NC1	1.04	FBXO7si+NC-1	0.95	FBXO7si+NC-1	1.08	FBXO7si+NC-1	0.81
OE-NC2	0.88	FBXO7si+NC-2	1.13	FBXO7si+NC-2	0.83	FBXO7si+NC-2	1.06
OE-NC3	1.08	FBXO7si+NC-3	0.98	FBXO7si+NC-3	1.09	FBXO7si+NC-3	1.13
OE-FBXO7-1	0.43	FBXO7si+PINKsi-1	2.11	FBXO7si+PINKsi-1	1.99	FBXO7si+PINKsi-1	1.87
OE-FBXO7-2	0.49	FBXO7si+PINKsi-2	1.95	FBXO7si+PINKsi-2	1.82	FBXO7si+PINKsi-2	2.11
OE-FBXO7-3	0.37	FBXO7si+PINKsi-3	2.02	FBXO7si+PINKsi-3	1.67	FBXO7si+PINKsi-3	2.03

SF3B-Col I		SF3B-B-SMB		SF3B-FN	
FBXO7+NC-1	1.13	FBXO7+NC-1	1.10	FBXO7+NC-1	1.22
FBXO7+NC-2	0.98	FBXO7+NC-2	1.02	FBXO7+NC-2	1.00
FBXO7+NC-3	0.89	FBXO7+NC-3	0.88	FBXO7+NC-3	0.78
FBXO7+PINK1-1	0.38	FBXO7+PINK1-1	0.66	FBXO7+PINK1-1	0.65
FBXO7+PINK1-2	0.58	FBXO7+PINK1-2	0.54	FBXO7+PINK1-2	0.43
FBXO7+PINK1-3	0.60	FBXO7+PINK1-3	0.38	FBXO7+PINK1-3	0.55
SF3B-Col I		SF3B-B-SMB		SF3B-FN	
FBXO7si+NC-1	0.83	FBXO7si+NC-1	0.95	FBXO7si+NC-1	0.92
FBXO7si+NC-2	1.04	FBXO7si+NC-2	1.17	FBXO7si+NC-2	1.17
FBXO7si+NC-3	1.13	FBXO7si+NC-3	0.88	FBXO7si+NC-3	0.91
FBXO7si+PINKsi-1	1.76	FBXO7si+PINKsi-1	2.01	FBXO7si+PINKsi-1	1.60
FBXO7si+PINKsi-2	1.90	FBXO7si+PINKsi-2	1.89	FBXO7si+PINKsi-2	1.99
FBXO7si+PINKsi-3	1.67	FBXO7si+PINKsi-3	1.80	FBXO7si+PINKsi-3	1.77

F6-SF3C-PINK1		F6-SF3C-LC3B		F6-SF3C-P62		F6-SF3C-TOMM20		F6-SF3C-DRP1		F6D-JC1	
NG1	1.20	NG1	1.09	NG1	1.16	NG1	0.93	NG1	0.81	NG1	0.89
NG2	0.81	NG2	1.01	NG2	1.08	NG2	0.87	NG2	1.02	NG2	1.07
NG3	0.99	NG3	0.90	NG3	0.76	NG3	1.00	NG3	1.17	NG3	1.04
HG1	0.38	HG1	0.31	HG1	2.28	HG1	0.56	HG1	2.26	HG1	0.53
HG2	0.49	HG2	0.56	HG2	1.93	HG2	0.49	HG2	2.03	HG2	0.57
HG3	0.23	HG3	0.55	HG3	2.11	HG3	0.38	HG3	1.82	HG3	0.42
HG+NC1	0.51	HG+NC1	0.27	HG+NC1	2.12	HG+NC1	0.37	HG+NC1	1.97	HG+NC1	0.61
HG+NC2	0.31	HG+NC2	0.35	HG+NC2	2.23	HG+NC2	0.47	HG+NC2	2.30	HG+NC2	0.37
HG+NC3	0.36	HG+NC3	0.49	HG+NC3	2.21	HG+NC3	0.31	HG+NC3	1.93	HG+NC3	0.45
HG+siRNA1	0.51	HG+siRNA1	0.98	HG+siRNA1	1.57	HG+siRNA1	0.78	HG+siRNA1	1.38	HG+siRNA1	1.21
HG+siRNA2	0.27	HG+siRNA2	1.19	HG+siRNA2	1.09	HG+siRNA2	0.90	HG+siRNA2	1.56	HG+siRNA2	1.04
HG+siRNA3	0.53	HG+siRNA3	0.92	HG+siRNA3	1.28	HG+siRNA3	0.69	HG+siRNA3	1.39	HG+siRNA3	0.96

F6C-PINK1		F6C-LC3B		F6C-P62		F6C-TOMM20		F6C-DRP1		F6D-MITOSOX	
NG1	1.19	NG1	0.90	NG1	1.10	NG1	1.05	NG1	1.05	NG1	0.88
NG2	0.85	NG2	0.88	NG2	1.08	NG2	1.19	NG2	1.11	NG2	1.17
NG3	0.96	NG3	1.22	NG3	0.82	NG3	0.76	NG3	0.84	NG3	0.95
HG1	0.38	HG1	0.53	HG1	1.83	HG1	0.35	HG1	1.95	HG1	2.86
HG2	0.47	HG2	0.58	HG2	1.74	HG2	0.59	HG2	1.84	HG2	2.67
HG3	0.36	HG3	0.28	HG3	2.36	HG3	0.49	HG3	2.13	HG3	3.01
HG+NC1	0.39	HG+NC1	0.66	HG+NC1	2.02	HG+NC1	0.47	HG+NC1	1.97	HG+NC1	2.91
HG+NC2	0.27	HG+NC2	0.36	HG+NC2	1.91	HG+NC2	0.48	HG+NC2	2.17	HG+NC2	3.13
HG+NC3	0.48	HG+NC3	0.48	HG+NC3	1.89	HG+NC3	0.37	HG+NC3	1.90	HG+NC3	3.22
HG+siRNA1	0.89	HG+siRNA1	1.03	HG+siRNA1	1.28	HG+siRNA1	0.78	HG+siRNA1	1.24	HG+siRNA1	1.19
HG+siRNA2	1.02	HG+siRNA2	0.96	HG+siRNA2	1.34	HG+siRNA2	0.69	HG+siRNA2	1.56	HG+siRNA2	1.06
HG+siRNA3	0.72	HG+siRNA3	1.24	HG+siRNA3	1.17	HG+siRNA3	0.91	HG+siRNA3	1.19	HG+siRNA3	1.24

F6-SF3D-PINK1		F6-SF3D-LC3B		F6-SF3D-P62		F6-SF3D-TOMM20		F6-SF3D-DRP1	
control1	0.93	control1	0.97	control1	1.12	control1	1.13	control1	1.07
control2	0.84	control2	1.19	control2	0.81	control2	0.91	control2	1.12
control3	1.07	control3	0.93	control3	0.94	control3	1.01	control3	1.05
control4	0.82	control4	0.97	control4	0.90	control4	0.91	control4	0.85
control5	1.02	control5	1.06	control5	0.88	control5	0.95	control5	0.98
control6	1.01	control6	1.19	control6	1.05	control6	0.81	control6	0.88
control7	1.31	control7	0.69	control7	1.30	control7	1.28	control7	1.05
DKD1	0.31	DKD1	0.67	DKD1	1.32	DKD1	0.40	DKD1	2.05
DKD2	0.48	DKD2	0.62	DKD2	1.91	DKD2	0.33	DKD2	1.77
DKD3	0.64	DKD3	0.35	DKD3	1.54	DKD3	0.49	DKD3	2.17
DKD4	0.54	DKD4	0.36	DKD4	1.81	DKD4	0.35	DKD4	1.80
DKD5	0.59	DKD5	0.25	DKD5	1.52	DKD5	0.29	DKD5	2.20
DKD6	0.61	DKD6	0.43	DKD6	1.86	DKD6	0.24	DKD6	1.82
DKD7	0.34	DKD7	0.61	DKD7	1.68	DKD7	0.49	DKD7	1.40
AAV-NC1	0.27	AAV-NC1	0.38	AAV-NC1	1.91	AAV-NC1	0.36	AAV-NC1	1.66
AAV-NC2	0.30	AAV-NC2	0.34	AAV-NC2	2.12	AAV-NC2	0.27	AAV-NC2	1.78
AAV-NC3	0.54	AAV-NC3	0.65	AAV-NC3	2.17	AAV-NC3	0.56	AAV-NC3	2.28
AAV-NC4	0.58	AAV-NC4	0.55	AAV-NC4	1.59	AAV-NC4	0.26	AAV-NC4	1.40
AAV-NC5	0.49	AAV-NC5	0.63	AAV-NC5	1.38	AAV-NC5	0.51	AAV-NC5	2.09
AAV-NC6	0.25	AAV-NC6	0.46	AAV-NC6	1.43	AAV-NC6	0.36	AAV-NC6	2.16
AAV-NC7	0.66	AAV-NC7	0.61	AAV-NC7	1.67	AAV-NC7	0.44	AAV-NC7	1.90
FBXO7RNAi1	0.49	FBXO7RNAi1	1.08	FBXO7RNAi1	1.26	FBXO7RNAi1	0.78	FBXO7RNAi1	1.55
FBXO7RNAi2	0.61	FBXO7RNAi2	0.89	FBXO7RNAi2	1.36	FBXO7RNAi2	0.97	FBXO7RNAi2	1.36
FBXO7RNAi3	0.59	FBXO7RNAi3	1.23	FBXO7RNAi3	1.13	FBXO7RNAi3	0.84	FBXO7RNAi3	1.44
FBXO7RNAi4	0.23	FBXO7RNAi4	1.07	FBXO7RNAi4	1.28	FBXO7RNAi4	0.89	FBXO7RNAi4	1.45
FBXO7RNAi5	0.34	FBXO7RNAi5	0.82	FBXO7RNAi5	1.37	FBXO7RNAi5	1.02	FBXO7RNAi5	1.55
FBXO7RNAi6	0.41	FBXO7RNAi6	0.90	FBXO7RNAi6	1.45	FBXO7RNAi6	0.98	FBXO7RNAi6	1.49
FBXO7RNAi7	0.53	FBXO7RNAi7	1.10	FBXO7RNAi7	1.23	FBXO7RNAi7	0.73	FBXO7RNAi7	1.57

F6G-PINK1		F6G-LC3B		F6G-P62		F6G-TOMM20		F6G-DRP1	
control1	1.03	control1	0.92	control1	0.82	control1	1.08	control1	0.92
control2	0.87	control2	0.83	control2	1.18	control2	1.18	control2	0.89
control3	1.04	control3	1.10	control3	1.10	control3	0.91	control3	1.03
control4	0.92	control4	0.87	control4	0.92	control4	1.11	control4	1.10
control5	0.97	control5	1.16	control5	0.99	control5	0.83	control5	1.02
control6	0.91	control6	1.03	control6	1.08	control6	0.97	control6	1.08
control7	1.26	control7	1.09	control7	0.91	control7	0.92	control7	0.96
DKD1	0.41	DKD1	0.58	DKD1	2.27	DKD1	0.43	DKD1	1.99
DKD2	0.44	DKD2	0.45	DKD2	1.94	DKD2	0.59	DKD2	1.91
DKD3	0.36	DKD3	0.40	DKD3	1.90	DKD3	0.57	DKD3	2.10
DKD4	0.23	DKD4	0.39	DKD4	2.21	DKD4	0.35	DKD4	1.78
DKD5	0.39	DKD5	0.50	DKD5	2.18	DKD5	0.47	DKD5	2.18
DKD6	0.49	DKD6	0.48	DKD6	2.27	DKD6	0.66	DKD6	1.88
DKD7	0.47	DKD7	0.33	DKD7	1.74	DKD7	0.28	DKD7	2.26
AAV-NC1	0.26	AAV-NC1	0.52	AAV-NC1	2.20	AAV-NC1	0.62	AAV-NC1	1.83
AAV-NC2	0.48	AAV-NC2	0.41	AAV-NC2	1.71	AAV-NC2	0.36	AAV-NC2	2.09
AAV-NC3	0.38	AAV-NC3	0.40	AAV-NC3	2.25	AAV-NC3	0.58	AAV-NC3	2.25
AAV-NC4	0.22	AAV-NC4	0.47	AAV-NC4	2.15	AAV-NC4	0.51	AAV-NC4	2.23
AAV-NC5	0.39	AAV-NC5	0.60	AAV-NC5	1.63	AAV-NC5	0.48	AAV-NC5	2.14
AAV-NC6	0.49	AAV-NC6	0.25	AAV-NC6	1.93	AAV-NC6	0.37	AAV-NC6	2.02
AAV-NC7	0.40	AAV-NC7	0.32	AAV-NC7	1.88	AAV-NC7	0.42	AAV-NC7	1.87
FBXO7RNAi1	0.75	FBXO7RNAi1	0.72	FBXO7RNAi1	1.30	FBXO7RNAi1	0.67	FBXO7RNAi1	1.37
FBXO7RNAi2	0.67	FBXO7RNAi2	0.87	FBXO7RNAi2	1.55	FBXO7RNAi2	0.86	FBXO7RNAi2	1.43
FBXO7RNAi3	0.81	FBXO7RNAi3	1.07	FBXO7RNAi3	1.63	FBXO7RNAi3	0.59	FBXO7RNAi3	1.63
FBXO7RNAi4	1.09	FBXO7RNAi4	0.83	FBXO7RNAi4	1.52	FBXO7RNAi4	0.65	FBXO7RNAi4	1.76
FBXO7RNAi5	0.66	FBXO7RNAi5	0.78	FBXO7RNAi5	1.39	FBXO7RNAi5	0.84	FBXO7RNAi5	1.80
FBXO7RNAi6	0.91	FBXO7RNAi6	0.82	FBXO7RNAi6	1.45	FBXO7RNAi6	0.89	FBXO7RNAi6	1.75
FBXO7RNAi7	0.52	FBXO7RNAi7	0.61	FBXO7RNAi7	1.36	FBXO7RNAi7	0.57	FBXO7RNAi7	1.39

F6F-LC3B		F6F-P62	
control1	94.60	control1	5.97
control2	89.18	control2	5.51
control3	86.47	control3	7.23
control4	91.11	control4	7.79
control5	90.88	control5	9.92
control6	83.40	control6	8.62
control7	88.16	control7	10.92
DKD1	32.47	DKD1	54.74
DKD2	34.27	DKD2	64.51
DKD3	22.59	DKD3	57.31
DKD4	22.72	DKD4	72.99
DKD5	30.71	DKD5	60.80
DKD6	33.80	DKD6	58.85
DKD7	32.59	DKD7	72.50
AAV-NC1	32.13	AAV-NC1	58.44
AAV-NC2	25.44	AAV-NC2	72.74
AAV-NC3	31.16	AAV-NC3	61.55
AAV-NC4	30.63	AAV-NC4	68.68
AAV-NC5	34.05	AAV-NC5	55.61
AAV-NC6	28.33	AAV-NC6	60.38
AAV-NC7	28.40	AAV-NC7	69.78
FBXO7RNAi1	83.38	FBXO7RNAi1	21.19
FBXO7RNAi2	75.05	FBXO7RNAi2	34.82
FBXO7RNAi3	82.35	FBXO7RNAi3	33.27
FBXO7RNAi4	83.05	FBXO7RNAi4	29.30
FBXO7RNAi5	77.12	FBXO7RNAi5	27.83
FBXO7RNAi6	72.49	FBXO7RNAi6	26.73
FBXO7RNAi7	78.07	FBXO7RNAi7	34.95

F7A-LC3B		F7A-P62		F7-SF3E-PINK1		F7-SF3E-LC3B		F7-SF3E-P62		F7-SF3E-TOMM20		F7-SF3E-DRP1	
control1	89.98	control1	8.41	control1	0.82	control1	0.99	control1	1.02	control1	0.96	control1	1.10
control2	94.89	control2	7.72	control2	0.89	control2	0.97	control2	1.14	control2	1.15	control2	1.01
control3	95.75	control3	9.65	control3	0.83	control3	0.94	control3	0.94	control3	1.05	control3	1.07
control4	94.33	control4	10.10	control4	1.18	control4	1.00	control4	0.90	control4	0.97	control4	1.04
control5	89.87	control5	5.14	control5	1.04	control5	1.18	control5	1.16	control5	0.92	control5	0.81
control6	92.22	control6	8.35	control6	1.17	control6	0.86	control6	1.10	control6	0.97	control6	0.97
control7	93.57	control7	9.63	control7	1.07	control7	1.06	control7	0.74	control7	0.98	control7	1.00
DKD1	25.82	DKD1	64.29	DKD1	0.28	DKD1	0.26	DKD1	1.82	DKD1	0.53	DKD1	1.76
DKD2	38.40	DKD2	61.43	DKD2	0.57	DKD2	0.33	DKD2	1.75	DKD2	0.35	DKD2	2.08
DKD3	36.91	DKD3	65.91	DKD3	0.38	DKD3	0.36	DKD3	2.35	DKD3	0.33	DKD3	1.99
DKD4	32.45	DKD4	72.44	DKD4	0.52	DKD4	0.46	DKD4	2.58	DKD4	0.36	DKD4	1.90
DKD5	33.19	DKD5	69.83	DKD5	0.50	DKD5	0.25	DKD5	1.83	DKD5	0.38	DKD5	1.53
DKD6	25.84	DKD6	60.37	DKD6	0.38	DKD6	0.45	DKD6	2.33	DKD6	0.43	DKD6	1.84
DKD7	35.47	DKD7	67.07	DKD7	0.37	DKD7	0.60	DKD7	1.78	DKD7	0.44	DKD7	1.51
tRF-NC1	27.84	tRF-NC1	64.82	tRF-NC1	0.31	tRF-NC1	0.41	tRF-NC1	2.05	tRF-NC1	0.29	tRF-NC1	1.44
tRF-NC2	32.97	tRF-NC2	70.03	tRF-NC2	0.25	tRF-NC2	0.31	tRF-NC2	1.82	tRF-NC2	0.31	tRF-NC2	2.16
tRF-NC3	29.98	tRF-NC3	67.86	tRF-NC3	0.63	tRF-NC3	0.37	tRF-NC3	1.89	tRF-NC3	0.56	tRF-NC3	1.54
tRF-NC4	27.81	tRF-NC4	67.20	tRF-NC4	0.57	tRF-NC4	0.30	tRF-NC4	1.95	tRF-NC4	0.54	tRF-NC4	1.51
tRF-NC5	36.18	tRF-NC5	60.53	tRF-NC5	0.47	tRF-NC5	0.47	tRF-NC5	1.90	tRF-NC5	0.40	tRF-NC5	1.81
tRF-NC6	31.40	tRF-NC6	67.17	tRF-NC6	0.68	tRF-NC6	0.33	tRF-NC6	2.06	tRF-NC6	0.44	tRF-NC6	1.95
tRF-NC7	28.13	tRF-NC7	57.65	tRF-NC7	0.27	tRF-NC7	0.40	tRF-NC7	2.50	tRF-NC7	0.62	tRF-NC7	2.17
AAV-tRF1	85.52	AAV-tRF1	32.07	AAV-tRF1	0.48	AAV-tRF1	0.92	AAV-tRF1	1.68	AAV-tRF1	0.54	AAV-tRF1	1.20
AAV-tRF2	85.98	AAV-tRF2	26.76	AAV-tRF2	0.60	AAV-tRF2	0.51	AAV-tRF2	1.74	AAV-tRF2	0.76	AAV-tRF2	1.39
AAV-tRF3	84.10	AAV-tRF3	30.87	AAV-tRF3	0.47	AAV-tRF3	1.02	AAV-tRF3	1.34	AAV-tRF3	0.83	AAV-tRF3	1.74
AAV-tRF4	80.68	AAV-tRF4	32.45	AAV-tRF4	0.50	AAV-tRF4	0.87	AAV-tRF4	1.49	AAV-tRF4	0.85	AAV-tRF4	1.54
AAV-tRF5	56.64	AAV-tRF5	33.55	AAV-tRF5	0.27	AAV-tRF5	0.61	AAV-tRF5	1.21	AAV-tRF5	1.03	AAV-tRF5	1.63
AAV-tRF6	78.36	AAV-tRF6	23.67	AAV-tRF6	0.38	AAV-tRF6	0.47	AAV-tRF6	1.34	AAV-tRF6	0.50	AAV-tRF6	1.73
AAV-tRF7	79.84	AAV-tRF7	33.81	AAV-tRF7	0.51	AAV-tRF7	0.89	AAV-tRF7	1.24	AAV-tRF7	0.96	AAV-tRF7	1.49

F7B-PINK1		F7B-LC3		F7B-P62		F7B-TOMM20		F7B-DRP1	
control1	1.05	control1	0.98	control1	1.18	control1	1.05	control1	1.02
control2	0.94	control2	1.00	control2	1.15	control2	1.13	control2	0.85
control3	1.01	control3	0.87	control3	0.80	control3	1.12	control3	0.89
control4	0.91	control4	0.82	control4	1.16	control4	1.19	control4	0.93
control5	0.82	control5	0.85	control5	0.97	control5	0.93	control5	1.02
control6	1.10	control6	1.16	control6	0.93	control6	0.94	control6	1.07
control7	1.17	control7	1.32	control7	0.81	control7	0.64	control7	1.22
DKD1	0.36	DKD1	0.48	DKD1	1.77	DKD1	0.32	DKD1	1.62
DKD2	0.35	DKD2	0.68	DKD2	1.84	DKD2	0.35	DKD2	1.76
DKD3	0.32	DKD3	0.28	DKD3	1.49	DKD3	0.31	DKD3	1.40
DKD4	0.63	DKD4	0.26	DKD4	1.96	DKD4	0.63	DKD4	2.31
DKD5	0.33	DKD5	0.38	DKD5	1.80	DKD5	0.47	DKD5	2.29
DKD6	0.43	DKD6	0.66	DKD6	1.61	DKD6	0.40	DKD6	1.54
DKD7	0.36	DKD7	0.51	DKD7	2.13	DKD7	0.31	DKD7	2.38
tRF-NC1	0.50	tRF-NC1	0.27	tRF-NC1	1.45	tRF-NC1	0.48	tRF-NC1	1.60
tRF-NC2	0.30	tRF-NC2	0.45	tRF-NC2	1.95	tRF-NC2	0.37	tRF-NC2	2.23
tRF-NC3	0.28	tRF-NC3	0.43	tRF-NC3	1.90	tRF-NC3	0.35	tRF-NC3	2.05
tRF-NC4	0.48	tRF-NC4	0.31	tRF-NC4	2.04	tRF-NC4	0.44	tRF-NC4	1.98
tRF-NC5	0.27	tRF-NC5	0.41	tRF-NC5	1.88	tRF-NC5	0.33	tRF-NC5	1.49
tRF-NC6	0.52	tRF-NC6	0.27	tRF-NC6	2.11	tRF-NC6	0.39	tRF-NC6	1.89
tRF-NC7	0.42	tRF-NC7	0.49	tRF-NC7	1.83	tRF-NC7	0.60	tRF-NC7	1.94
AAV-tRF1	1.02	AAV-tRF1	1.15	AAV-tRF1	1.34	AAV-tRF1	0.63	AAV-tRF1	1.33
AAV-tRF2	0.76	AAV-tRF2	0.52	AAV-tRF2	1.26	AAV-tRF2	0.67	AAV-tRF2	1.68
AAV-tRF3	0.60	AAV-tRF3	1.06	AAV-tRF3	1.48	AAV-tRF3	0.75	AAV-tRF3	1.40
AAV-tRF4	0.56	AAV-tRF4	0.59	AAV-tRF4	1.38	AAV-tRF4	0.56	AAV-tRF4	1.60
AAV-tRF5	0.86	AAV-tRF5	0.89	AAV-tRF5	1.33	AAV-tRF5	0.70	AAV-tRF5	1.58
AAV-tRF6	0.73	AAV-tRF6	0.48	AAV-tRF6	1.29	AAV-tRF6	0.77	AAV-tRF6	1.31
AAV-tRF7	0.55	AAV-tRF7	0.64	AAV-tRF7	1.23	AAV-tRF7	0.81	AAV-tRF7	1.20

F7-SF3F-PINK1		F7-SF3F-LC3B		F7-SF3F-P62		F7-SF3F-TOMM20		F7-SF3F-DRP1	
NG1	1.14	NG1	0.86	NG1	0.88	NG1	1.13	NG1	1.20
NG2	0.89	NG2	0.94	NG2	0.95	NG2	1.02	NG2	0.91
NG3	0.97	NG3	1.20	NG3	1.17	NG3	0.85	NG3	0.89
HG1	0.44	HG1	0.39	HG1	1.74	HG1	0.23	HG1	1.68
HG2	0.3	HG2	0.54	HG2	2.11	HG2	0.39	HG2	1.84
HG3	0.37	HG3	0.25	HG3	2.16	HG3	0.46	HG3	2.12
tRF-NC1	0.52	tRF-NC1	0.55	tRF-NC1	1.70	tRF-NC1	0.30	tRF-NC1	1.61
tRF-NC2	0.25	tRF-NC2	0.23	tRF-NC2	2.31	tRF-NC2	0.43	tRF-NC2	2.00
tRF-NC3	0.38	tRF-NC3	0.44	tRF-NC3	2.22	tRF-NC3	0.61	tRF-NC3	1.85
tRF-mimic1	0.44	tRF-mimic1	1.04	tRF-mimic1	1.22	tRF-mimic1	0.82	tRF-mimic1	1.33
tRF-mimic2	0.53	tRF-mimic2	0.91	tRF-mimic2	1.34	tRF-mimic2	0.73	tRF-mimic2	1.35
tRF-mimic3	0.32	tRF-mimic3	0.81	tRF-mimic3	1.56	tRF-mimic3	0.69	tRF-mimic3	1.50

F7D-PINK1		F7D-LC3		F7D-P62		F7D-TOMM20		F7D-DRP1	
NG1	0.83	NG1	1.01	NG1	0.80	NG1	0.99	NG1	1.07
NG2	1.10	NG2	0.91	NG2	0.92	NG2	0.81	NG2	0.86
NG3	1.07	NG3	1.08	NG3	1.28	NG3	1.20	NG3	1.07
HG1	0.54	HG1	0.49	HG1	1.56	HG1	0.55	HG1	2.21
HG2	0.42	HG2	0.59	HG2	1.59	HG2	0.33	HG2	1.99
HG3	0.62	HG3	0.31	HG3	2.20	HG3	0.51	HG3	1.70
tRF-NC1	0.34	tRF-NC1	0.63	tRF-NC1	1.97	tRF-NC1	0.56	tRF-NC1	2.06
tRF-NC2	0.55	tRF-NC2	0.66	tRF-NC2	2.18	tRF-NC2	0.32	tRF-NC2	1.79
tRF-NC3	0.42	tRF-NC3	0.65	tRF-NC3	2.15	tRF-NC3	0.65	tRF-NC3	2.13
tRF-mimic1	0.80	tRF-mimic1	0.87	tRF-mimic1	1.28	tRF-mimic1	0.90	tRF-mimic1	1.72
tRF-mimic2	0.68	tRF-mimic2	0.97	tRF-mimic2	1.62	tRF-mimic2	1.04	tRF-mimic2	1.40
tRF-mimic3	1.06	tRF-mimic3	0.76	tRF-mimic3	1.39	tRF-mimic3	1.11	tRF-mimic3	1.28

F7E-JC1		F7E-MITOSOX	
NG1	0.92	NG1	1.06
NG2	0.95	NG2	0.84
NG3	1.13	NG3	1.10
HG1	0.58	HG1	2.88
HG2	0.46	HG2	3.24
HG3	0.6	HG3	3.11
tRF-NC1	0.47	tRF-NC1	2.96
tRF-NC2	0.61	tRF-NC2	2.79
tRF-NC3	0.65	tRF-NC3	3.19
tRF-mimic1	1.12	tRF-mimic1	1.17
tRF-mimic2	0.88	tRF-mimic2	1.04
tRF-mimic3	0.91	tRF-mimic3	0.90

F7F-LC3		F7F-P62		F7F-Col I		F7F-a-SMA		F7F-FN	
HG+NC+DMSO1	1.08	HG+NC+DMSO1	0.95	HG+NC+DMSO1	0.98	HG+NC+DMSO1	0.86	HG+NC+DMSO1	0.95
HG+NC+DMSO2	0.80	HG+NC+DMSO2	1.03	HG+NC+DMSO2	1.16	HG+NC+DMSO2	1.05	HG+NC+DMSO2	0.99
HG+NC+DMSO3	1.12	HG+NC+DMSO3	1.02	HG+NC+DMSO3	0.86	HG+NC+DMSO3	1.09	HG+NC+DMSO3	1.05
HG+tRF+DMSO1	2.49	HG+tRF+DMSO1	0.53	HG+tRF+DMSO1	0.45	HG+tRF+DMSO1	0.61	HG+tRF+DMSO1	0.60
HG+tRF+DMSO2	2.68	HG+tRF+DMSO2	0.43	HG+tRF+DMSO2	0.60	HG+tRF+DMSO2	0.39	HG+tRF+DMSO2	0.44
HG+tRF+DMSO3	2.05	HG+tRF+DMSO3	0.58	HG+tRF+DMSO3	0.55	HG+tRF+DMSO3	0.58	HG+tRF+DMSO3	0.56
HG+NC+BafA1-1	2.34	HG+NC+BafA1-1	2.07	HG+NC+BafA1-1	2.83	HG+NC+BafA1-1	2.82	HG+NC+BafA1-1	2.63
HG+NC+BafA1-2	2.39	HG+NC+BafA1-2	2.48	HG+NC+BafA1-2	2.79	HG+NC+BafA1-2	2.27	HG+NC+BafA1-2	2.70
HG+NC+BafA1-3	2.18	HG+NC+BafA1-3	2.91	HG+NC+BafA1-3	2.56	HG+NC+BafA1-3	2.42	HG+NC+BafA1-3	2.59
HG+tRF+BafA1	2.77	HG+tRF+BafA1	2.97	HG+tRF+BafA1	2.98	HG+tRF+BafA1	2.88	HG+tRF+BafA1	2.81
HG+tRF+BafA2	2.93	HG+tRF+BafA2	2.76	HG+tRF+BafA2	2.69	HG+tRF+BafA2	2.35	HG+tRF+BafA2	2.66
HG+tRF+BafA3	2.98	HG+tRF+BafA3	2.85	HG+tRF+BafA3	2.40	HG+tRF+BafA3	2.87	HG+tRF+BafA3	2.56
