# Supplementary material for: Tubular tRF‑1:30‑Gln‑CTG‑4 attenuates tubulointerstitial fibrosis by mitigating FBXO7-mediated mitophagy defect in diabetic kidney disease
Source: Cell Death Dis. 2026 Jul 18;17(1):886. doi: 10.1038/s41419-026-09112-2 (PMC13661522; doi:10.1038/s41419-026-09112-2)
Supplement: Supplementary file 2 [file 41419_2026_9112_MOESM1_ESM.docx]

**Fig S1. tRF‑1:30‑Gln‑CTG‑4 overexpression alleviates mRNA expression of extracellular matrix *in vivo* and *in vitro***. (A) Relative mRNA expression of Col I, α-SMA and FN in *db/db* mice. (B) Relative mRNA expression of Col I, α-SMA and FN in HG-treated HK2 cells. (C) AAV-tRF‑1:30‑Gln‑CTG‑4 was injected via the tail vein (blue, DAPI; red, AAV- tRF‑1:30‑Gln‑CTG‑4). Scale bars, 50 μm. (D) Relative mRNA expression of tRF‑1:30‑Gln‑CTG‑4 in different organs of *db/db* mice with tRF‑1:30‑Gln‑CTG‑4 overexpression. (E) Relative mRNA expression of Col I, α-SMA and FN in *db/db* mice with tRF‑1:30‑Gln‑CTG‑4 overexpression. (F) Relative mRNA expression of Col I, α-SMA and FN in HG-treated HK-2 cells with tRF‑1:30‑Gln‑CTG‑4 mimic treatment. ****p* < 0.001; ^#^*p* < 0.05; ^###^*p* < 0.001; NS, no significant. tRF‑1:30‑Gln‑CTG‑4 was abbreviated to tRF-G130.

**Fig S2. Knockdown of FBXO7 mitigates extracellular matrix expression *in vivo* and *in vitro*.** (A) Western blot analysis of FBXO7. (B) AAV-FBXO7 RNAi was injected via the tail vein (blue, DAPI; green, AAV). Scale bars, 50 μm. (C) The co-localization of proximal tubular cell markers LTL and FBXO7 in renal tissue of *db/db* mice. (D) Relative mRNA expression of Col I, α-SMA and FN in *db/db* mice with FBXO7 knockdown. (E) Relative mRNA expression of Col I, α-SMA and FN in HG-treated HK-2 cells with FBXO7 knockdown. (F) Western blot analysis of FBXO7, Col I, α-SMA and FN expression after FBXO7 knockdown in HG-treated HK-2 cells. ***p* < 0.01; ****p* < 0.001; ^#^*p* < 0.05; ^##^*p* < 0.01; ^###^*p* < 0.001. tRF‑1:30‑Gln‑CTG‑4 was abbreviated to tRF-G130.

**Fig S3. The regulatory effects of tRF‑1:30‑Gln‑CTG‑4 and FBXO7-PINK1 on extracellular matrix deposition and mitophagy** (A) Relative mRNA expression of Col I, α-SMA and FN in HG-treated HK-2 cells with both FBXO7 and PINK1 overexpression or knockdown. (B) Relative protein expression of Col I, α-SMA and FN in HG-treated HK-2 cells with FBXO7 and PINK1 overexpression or knockdown. (C and D) Relative mRNA expression of PINK1, LC3B, P62, TOMM20 and DRP1 in HG-treated HK-2 cells or in *db/db* mice with FBXO7 knockdown. (E and F) Relative mRNA expression of PINK1, LC3B, P62, TOMM20 and DRP1 in *db/db* mice and in HG-treated HK-2 cells with tRF‑1:30‑Gln‑CTG‑4 overexpression. **p* < 0.05; ***p* < 0.01; ****p* < 0.001; ^##^*p* < 0.01; ^###^*p* < 0.001; NS, no significant. tRF‑1:30‑Gln‑CTG‑4 was abbreviated to tRF-G130.

**Supplementary Table I. Primers for mRNAs.**

| **Primer** | **Forward (5′-3′)** | **Reverse (5′-3′)** |
| --- | --- | --- |
| Mus α-SMA | CAGCAAACAGGAATACGACGAA | AACCACGAGTAACAAATCAAAGC |
| Mus Col I | GTCAGACCTGTGTGTTCCCTACTCA | TCTCTCCAAACCAGACGTGCTTC |
| Mus FN | GCAAGAAGGACAACCGAGGAAA | GGACATCAGTGAAGGAGCCAGA |
| Mus PINK1 | CATCGCCTATGAAATCTTTGGG | AATTTCAGGTTCTTCAGGGCTA |
| Mus P62 | GCCAGAGGAACAGATGGA | GGGAGATGTGGGTATAGGG |
| Mus TOMM20 | GCTGCAAGTGTTACAGCAGA | GTCGGAAGCTTGGTCAGAAG |
| Mus DRP1 | AACCAACAACAGGCAACTGGAGAG | AACCTCACAATCTCGCTGTTCTCG |
| Mus β-actin | GGCTGTATTCCCCTCCATCG | CCAGTTGGTAACAATGCCATGT |
| Homo α-SMA | ATGGTGATGACCTGCCCGTCT | ACTGAGCGTGGCTACTCCTTCG |
| Homo Col I | GGTAGGTGATGTTCTGGGAGGC | CAAGAGGCATGTCTGGTTCGG |
| Homo FN | AACCTTGCTCCTGACAGCTC | TTGGTGGGCTGACATTCTCC |
| Homo PINK1 | TGACCTTTGCCCCTAACACGAG | GTAACTGAACGTGCTGACCCAT |
| Homo P62 | GATTCGCCGCTTCAGCTTCT | CCCGTCCTCATCGCGGTA |
| Homo TOMM20 | ACAGGCTTCGAGAACGAAGAA | TGTCAGATGGTCTACGCCCT |
| Homo DRP1 | TCACCCGGAGACCTCTCATT | TCTGCTTCCACCCCATTTTCT |
| Homo β-actin | CCAAAGTTCACAATGTGGCCG | TGGACTTGGGAGAGGACTGG |

α-SMA, α-smooth muscle actin; Col I, Collagen I; FN, Fibronectin; Mus, mus musculus; Homo, homo sapiens.

**Supplementary Table II. Primer for** **tRF-1:30-Gln-CTG-4.**

| **Gene** | **Forward primer sequence (5′-3′)** |
| --- | --- |
| tRF-1:30-Gln-CTG-4 | GGTTCCATGGTGTAATGGTGAGCACTCTGG |
